# Lysine polyphosphate modifications contribute to virulence factors in *Pseudomonas aeruginosa*

**DOI:** 10.1128/mbio.00855-25

**Published:** 2025-04-17

**Authors:** Kirsten Lehotsky, Nolan Neville, Isabella Martins, Keith Poole, Zongchao Jia

**Affiliations:** 1 Department of Biomedical and Molecular Sciences, Queen’s University, Kingston, Ontario, Canada; Columbia University, New York, New York, USA

**Keywords:** lysine polyphosphate modification, *Pseudomonas aeruginosa* virulence, biofilm formation, EngA and SrmB, lysine-rich motif

## Abstract

Inorganic polyphosphate (polyP) is a universally conserved polymer involved in various biological processes, but its role as a direct protein regulator remains largely unexplored. Lysine polyphosphate modification (KPM), a strong but non-covalent interaction between polyP and lysine-rich protein sequences, has not been functionally characterized until now. In this study, we present the first investigation into KPM’s biological significance using *Pseudomonas aeruginosa*, a critical priority pathogen known for its antibiotic resistance and virulence. We identified two essential bacterial proteins, EngA and SrmB, as novel KPM targets. Through site-specific lysine deletions, we demonstrated that disrupting lysine-polyP interactions severely impairs biofilm formation and significantly reduces the production of key virulence factors, including pyoverdine and pyocyanin. These findings establish a direct functional link between polyP and bacterial pathogenicity mediated by KPM. Our results highlight KPM as a previously unrecognized regulatory mechanism critical for controlling bacterial virulence factors. This work uncovers the first functional role of KPM and its importance in regulating virulence phenotypes in a major human pathogen.

**IMPORTANCE:**

Polyphosphate is commonly known for its roles in metabolism and stress response. How inorganic polyphosphate (polyP) facilitates bacterial virulence has remained largely elusive. This study reveals that lysine polyphosphate modification (KPM), a chemical interaction between polyP and lysine-rich proteins, is essential for bacterial survival and pathogenicity in *P. aeruginosa*, a harmful microbe responsible for difficult-to-treat infections. We discovered that disrupting KPM in key proteins impairs the bacteria’s ability to form protective biofilms and produce harmful toxins. This previously unknown biological process links polyP to protein function in controlling bacterial virulence factors. Our findings open new possibilities for developing anti-virulence therapies aimed at reducing bacterial infections without promoting antibiotic resistance.

## OBSERVATION

Inorganic polyphosphate (polyP) is a linear polymer of inorganic phosphate units linked by high-energy phosphoanhydride bonds. In bacteria, polyP accumulates through the action of polyphosphate kinases (PPK1 and PPK2s) (1, 2). *Pseudomonas aeruginosa* adapts to hostile environments by forming biofilms, while additional virulence factors like pyoverdine and pyocyanin contribute to iron scavenging and toxin production, respectively (3–5). PolyP plays a regulatory role in controlling these virulence factors (6). Inactivation of *ppk* genes in *P. aeruginosa* reduces motility, toxin secretion, and biofilm formation (7). While PPK inhibitors such as gallein have shown promise in infection models, the precise mechanisms linking polyP to virulence remain poorly understood (8).

Our previous research identified polyP-dependent modifications targeting histidine- and lysine-rich regions in proteins, termed histidine polyphosphate modification (HPM) and lysine polyphosphate modification (KPM), respectively (9, 10). While the functional impact of HPM has been studied (9), the physiological significance of KPM remains completely unexplored (11). Notably, KPM target proteins in this study are novel (containing three or more consecutive lysine residues) and not previously reported PASK proteins (a 20-amino-acid region containing 75% poly-acidic residues D, E, S, or K residues, with at least one lysine) (12).

**Fig 1:**
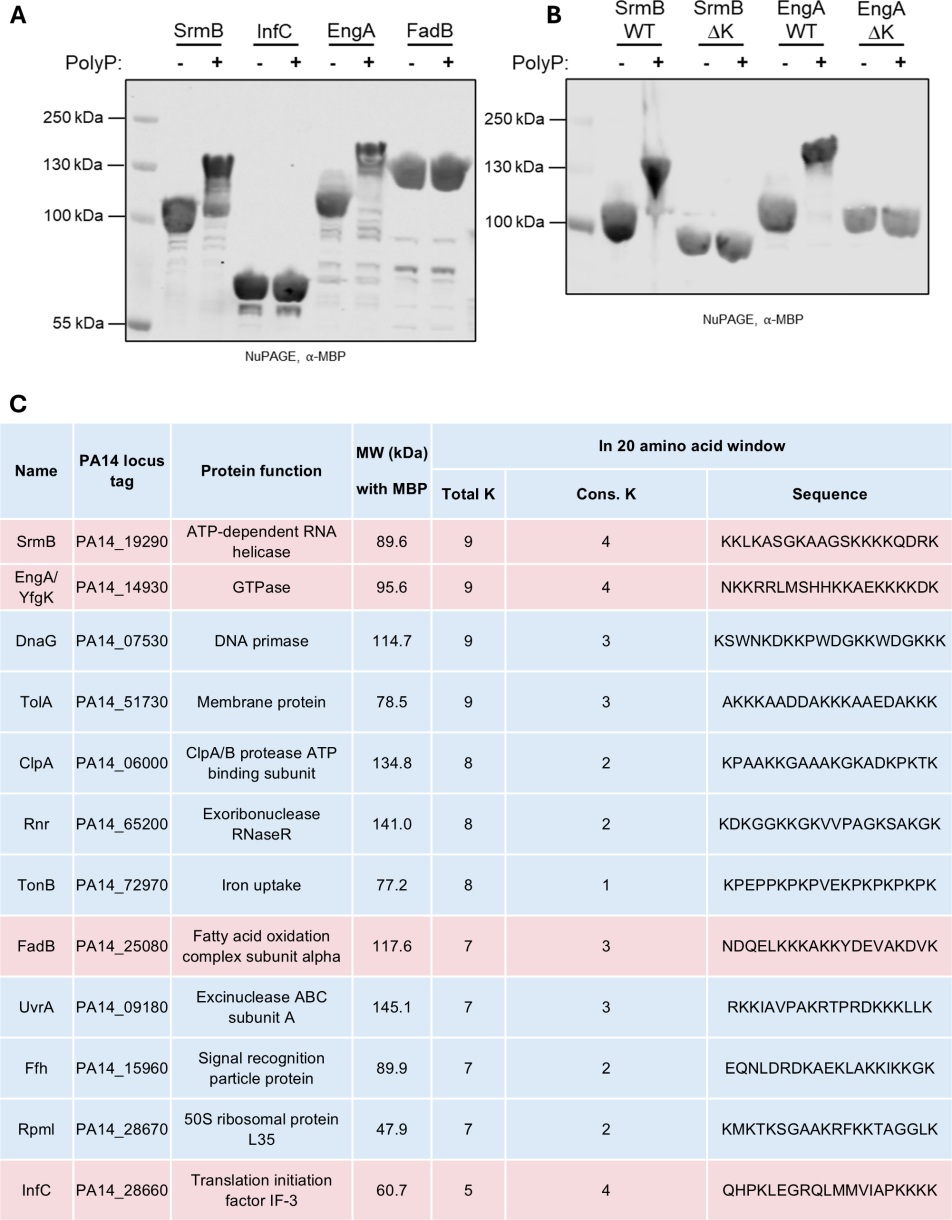
Screening of selected KPM targets in *P. aeruginosa*. (**A**) NuPAGE analysis of poly-lysine *P. aeruginosa* targets recombinantly expressed in BL21 RIPL *E. coli* cells in a custom MBP-fusion tag vector, HT25 (N-MBP-Protein). (**B**) NuPAGE analysis of C-terminal truncations of SrmB and EngA MBP fusions lacking their respective lysine-rich regions following *in vitro* polyP treatment as in **A**. SrmB ∆K consists of residues 1-386 (residues 387-446 deleted), and EngA ∆K consists of residues 1-472 (residues 473-493 deleted). (**C**) List of top 12 protein candidates that were identified and ranked based on total number of lysine residues in a sliding 20-residue window; red indicates the poly-lysine bacterial proteins that were screened for polyP binding in this study. For a full list, see Table S4.

Poly-lysine proteins were cloned and recombinantly expressed in *E. coli* BL21(DE3) cells using a custom pET-16-based vector HT25 (N-MBP-insert-C). PolyP-dependent electrophoretic mobility shifts were observed on NuPAGE gels and visualized via western blot with an anti-MBP antibody. We selected four *P. aeruginosa* proteins containing a Lys-rich sequence for polyP binding screening using the basic local alignment search tool to identify proteins with consecutive lysine residues within the UCBPP-PA14 proteome (Fig. 1C). Two of the four proteins, EngA and SrmB, were revealed to be targets of KPM (Fig. 1A), while InfC and FadB did not shift. After detecting NuPAGE shifts of EngA and SrmB, we next verified that the polyP modification was lysine-dependent through deletion experiments. Lysine deletion mutants (∆K) of *P. aeruginosa* EngA (residues 473-493 removed) and SrmB (residues 387-446 removed) abolished polyP-mediated NuPAGE shifts (Fig. 1B).

EngA and SrmB are known to play important roles in bacterial physiology. SrmB is an RNA helicase that has been implicated in ribosome assembly (13). EngA, also known as YfgK, is a double Era-like (Der) GTPase that also plays a role in ribosome maturation (14, 15). Interestingly, point mutations in *E. coli* EngA are suppressed by overexpression of RelA, an enzyme that synthesizes the (p)ppGpp alarmone (16). (p)ppGpp has been linked to polyP dynamics in *E. coli* (17–19)—albeit with caveats (20, 21)—raising a potential functional link between EngA and polyP.

We have previously demonstrated that robust, non-covalent polyP modifications depend on either lysine- or histidine-rich sequence, with KPM or HPM being positively correlated with longer stretches of lysine or histidine residues, respectively (11). Among the four lysine-rich proteins from *P. aeruginosa* that we tested, the positive hits EngA and SrmB each contain nine lysines within a 20-amino-acid region, including four consecutive lysines. In contrast, the negative hits InfC and FadB have five and seven lysines, with four and three consecutive lysines, respectively. Although our data set is limited, these findings suggest that the total number of lysines within the target region may play a more critical role in polyP interaction than the presence of consecutive lysine residues alone. We have used a custom Python script to rank the UCBPP-PA14 proteome (downloaded from the *Pseudomonas* Genome Data Bank) by lysine count within a 20-residue sliding window to help future KPM screening studies in *P. aeruginosa* (Fig. 1C; Table S4).

**Fig 2:**
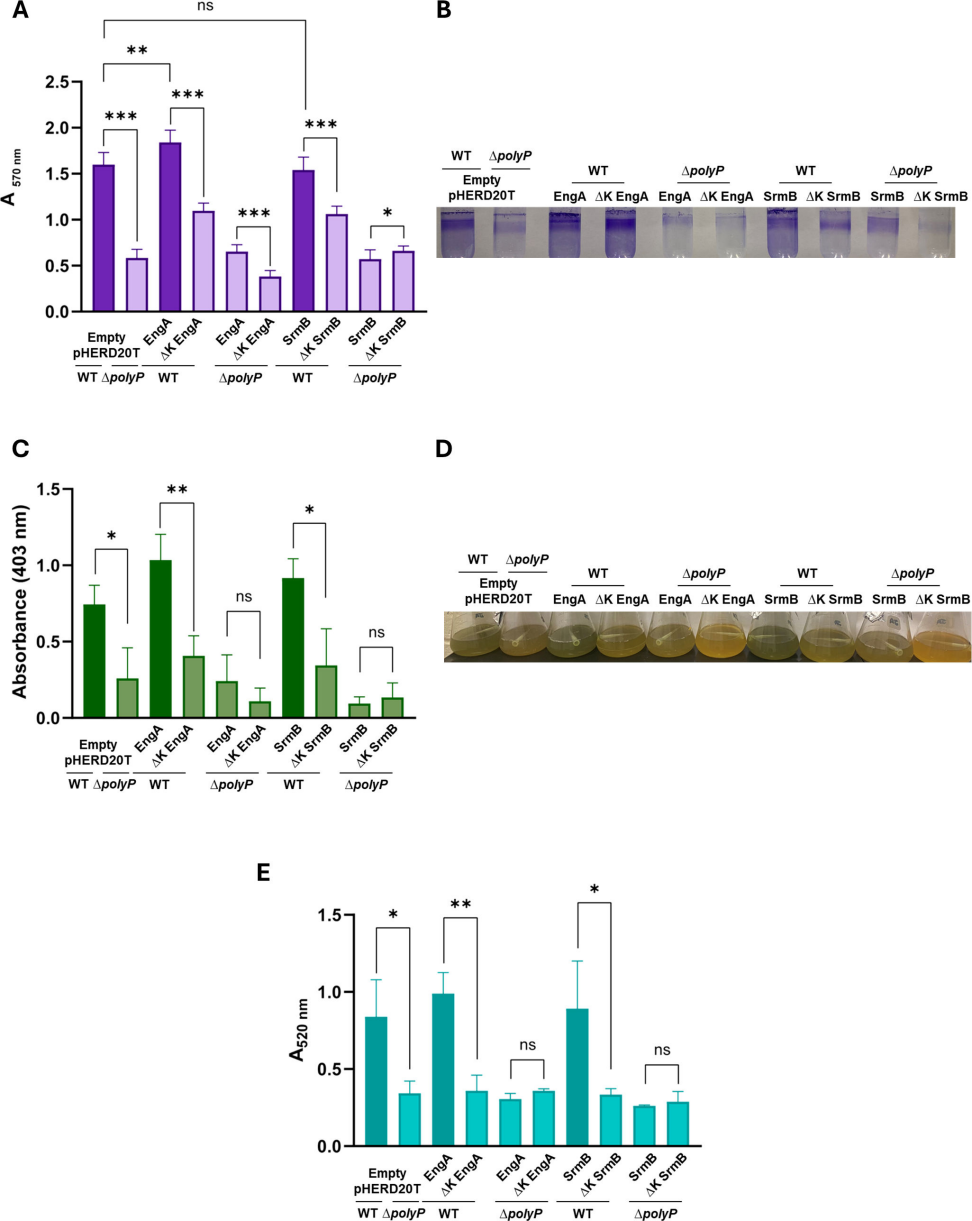
KPM modulates *P. aeruginosa* virulence factors. (**A**) Effect of recombinant overexpression of WT EngA and SrmB compared to ∆K mutants on biofilm formation. Biofilm formation was observed in WT PA14 and ∆*polyP* PA14 strains and quantified via crystal violet staining. (**B**) Representative image of biofilm ring formation. (**C**) Effect of recombinant overexpression of WT EngA and SrmB compared to ∆K mutants on pyoverdine production. Pyoverdine production was observed in WT PA14 and ∆All PA14 strains and quantified via absorbance at 403 nm. (**D**) Representative image of pyoverdine production. (**E**) Effect of recombinant overexpression of WT EngA and SrmB compared to ∆K mutants on pyocyanin production. Pyocyanin production was observed in WT PA14 and ∆*polyP* PA14 strains and quantified via absorbance at 520 nm following chloroform extraction and HCl treatment. Proteins were expressed using the pHERD20T arabinose-inducible expression vector (1% L-arabinose wt/vol). ns, *P* >0.05; ^*^, *P <*0.05; ^**^, *P* <0.01; ^***^, *P* <0.001 (unpaired *t*-test, *n* = 3 biological replicates). Error bars are ±standard deviation.

We evaluated biofilm formation, production of pyoverdine, which is an important siderophore involved in nutrient acquisition, as well as pyocyanin, a compound that is toxic to the host cell (Fig. 2). Biofilm formation was quantified using crystal violet staining and absorbance measurements at 570 nm, while pyoverdine was quantified directly by measuring absorbance at 403 nm. Pyocyanin was extracted from *P. aeruginosa* cultures using a chloroform and HCl treatment and then measured at 520 nm using a spectrophotometer.

We next performed these virulence-associated phenotype assays following the overexpression of wild-type (WT) and ∆K mutants of EngA and SrmB. All proteins were expressed in both WT and ∆*polyP* (deletion mutant of *ppk1* and *ppk2*s) PA14 cells using an arabinose-inducible vector (pHERD20T). As expected, empty pHERD20T confirmed that virulence factors were attenuated in ∆*polyP* compared to WT PA14 cells. WT PA14 cells overexpressing wild-type EngA or SrmB displayed virulent properties (biofilm, pyoverdine, and pyocyanin) similar to the empty control WT PA14 cells. However, interestingly, bacterial phenotype levels produced by wild-type PA14 cells overexpressing ∆K EngA or ∆K SrmB were equivalent to those observed in the control ∆*polyP* cells. Deletion of the lysine residues prevents KPM from occurring on either of these proteins with lysine-rich sequence stretches (Fig. 1B). Prevention of KPM through ∆K mutants resulted in reduced virulence phenotypes; as such, polyP has a pro-virulence factor effect via polyP modifications occurring on EngA and SrmB. In these experiments, the chromosomal WT copy of these genes is still present; therefore, we cannot exclude the possibility that WT proteins are still able to bind polyP, confounding virulence levels. Future knockout studies (i.e., ∆*engA* and ∆*srmB*) will provide more information as to whether these endogenous proteins are directly involved in virulence-associated pathways. Our efforts to achieve ∆*engA* and ∆*srmB* backgrounds were unsuccessful, suggesting that they are essential gene candidates in *P. aeruginosa*.

Expressing both the WT and mutant proteins within ∆*polyP* PA14 cells verified that a similar defect is observed regardless of the lysine-rich sequence being present or not. Altogether, this implies that deletion of lysine residues within EngA or SrmB is only capable of reducing virulence factors if in the presence of polyP (i.e., conditions conducive to forming polyP modifications). PolyP binding of EngA and SrmB is lysine dependent and not an artifact of protein misfolding as confirmed by circular dichroism (CD) spectroscopy. CD spectra of recombinantly purified WT EngA and WT SrmB were nearly equivalent to their ∆K mutant counterparts, suggesting that the lysine deletions did not interfere with folding properties or secondary structural elements (Fig. S1 and S2). This observation is further supported by the results from our SYPRO-Orange thermal shift assay (see Suppl. Material). Although the most logical and plausible explanation involves interactions between polyP and lysine-rich regions, we cannot exclude the possibility that the observed reduction in virulence factors in ∆K mutants may result from lysine deletions potentially incapacitating these essential proteins, independent of polyP. Functional studies conducted *in vitro* on both EngA and SrmB are required to test protein function sans lysine; however, either outcome will expand the list of antibacterial protein targets.

PolyP is a well-established regulator of bacterial virulence phenotypes. This was evidenced by the successful use of small-molecule inhibitors targeting PPK enzymes, which reduced bacterial polyP production and emerged as promising antivirulence agents (9). However, the mechanistic link between polyP and several virulence factors, including biofilm formation and toxin production (pyoverdine and pyocyanin), remains unclear. With the discovery of lysine-mediated polyP binding targets in *P. aeruginosa*, we propose that KPM of bacterial targets could represent a previously unrecognized biochemical link between polyP and virulence phenotypes.

Since EngA and SrmB function as GTPase and helicase proteins, respectively, it is reasonable to speculate that polyP might interfere with their activities. Indeed, overexpression of either EngA or SrmB in polyP-producing WT *P. aeruginosa* cells increased virulence phenotypes, specifically biofilm formation as well as pyoverdine and pyocyanin production, compared to ΔK mutants, which are unable to undergo KPM (Fig. 2). These results suggest that polyP may regulate virulence phenotypes through upstream functional effects mediated by these poly-lysine proteins.

SrmB has been shown *in vitro* to be reliant on ATPase activation for helicase unwinding via long oligoribonucleotides (22). Due to the large and negatively charged nature of polyP, it is plausible that KPM could also stimulate the ATPase activity of SrmB. Moreover, it has been proposed that the oligomeric state of SrmB impacts helicase activity (23). PolyP modifications could potentially affect the oligomerization state of SrmB, critical for substrate binding, ribosome biogenesis, and downstream translational machinery involved in generating virulent responses. Likewise, KPM of EngA could cause conformational changes between its two GTPase domains (GD1 and GD2) and its KH-domain (containing the lysine-rich region) (24). We suggest that KPM could alter the stabilization of these interfaces and, thus, potentially modulate the GTP/GDP turnover rate as well as EngA-ribosome interactions, both impacting the biosynthesis of downstream virulent factors such as enzymes involved in phenazine production (e.g., pyocyanin) (25).

In summary, our work not only demonstrates KPM’s functional significance for the first time but also posits a previously unknown mechanism linking polyP to bacterial virulence-associated factors. Similar to HPM, KPM’s physiological relevance hinges on critical factors such as adequate polyP levels and favourable cellular conditions (11). Our findings strongly suggest that KPM has important functional implications, as evidenced by its role in modulating virulence phenotypes. Since enhanced virulence traits contribute to increased pathogenicity, targeting KPM interactions—specifically disrupting EngA-polyP or SrmB-polyP binding—could serve as a promising new strategy for combating bacterial infections. This insight opens new avenues for developing anti-virulence therapies by leveraging the regulatory potential of polyP modifications.

## AFTER EPUB

*[This article was published on 17 April 2025 with errors in the figures. The figures were corrected in the current version, posted on 22 April 2025.]*

## SUPPLEMENTAL MATERIAL

Supplemental material mbio.00855-25-s0001.docx Supplemental methods, figures, and tables.

ASM does not own the copyrights to Supplemental Material that may be linked to, or accessed through, an article. The authors have granted ASM a non-exclusive, world-wide license to publish the Supplemental Material files. Please contact the corresponding author directly for reuse.
