# Erratum for Lehotsky et al., “Lysine polyphosphate modifications contribute to virulence factors in *Pseudomonas aeruginosa*”

**DOI:** 10.1128/mbio.02800-25

**Published:** 2025-09-23

**Authors:** Kirsten Lehotsky, Nolan Neville, Isabella Martins, Keith Poole, Zongchao Jia

## ERRATUM

Volume 16, no. 5, e00855-25, 2025, https://doi.org/10.1128/mbio.00855-25. Page 6, Acknowledgments, second sentence: “M. Fisher” should read “M. S. Fishman.”

