# Supplementary material for: Lysine polyphosphate modifications contribute to virulence factors in Pseudomonas aeruginosa
Source: mBio. 2025 Apr 17;16(5):e00855-25. doi: 10.1128/mbio.00855-25 (PMC12077175; doi:10.1128/mbio.00855-25)
Supplement: Supplemental material — Supplemental methods, figures, and tables. [file mbio.00855-25-s0001.docx]

**SUPPLEMENTAL MATERIAL**

**Lysine polyphosphate modifications contribute to virulence factors in *Pseudomonas aeruginosa***

Kirsten Lehotsky, Nolan Neville, Isabella Martins, Keith Poole, Zongchao Jia^*^

From the Department of Biomedical and Molecular Sciences, Queen’s University, Kingston, K7L 3N6, Canada

*Correspondence should be addressed to: [jia@queensu.ca](mailto:jia@queensu.ca)

**Mutagenesis and cloning**

Genes encoding the *P. aeruginosa* proteins were amplified from *P. aeruginosa* UCBPP-PA14 genomic DNA. All genes were PCR amplified using Q5 DNA Polymerase (New England Biolabs, Cat# M0491L) and cloned via restriction digest into our custom pET16-based vector HT25. Cloning into HT25 yields an N-terminal MBP tag followed by the protein of interest. Proteins that conferred considerable electrophoretic shifts on NuPAGE gels were selected for mutagenesis studies to confirm that shifting was lysine-dependent. Mutations were introduced via PCR based site directed mutagenesis methods, with custom mutagenic primers (**Table S1**) made to flank the region to be deleted/mutated as outlined by *Watson et al.* (1). Plasmid template DNA was removed by DpnI restriction enzyme digestion, incubated overnight at 37ºC, then transformed directly into Top10 or DH5α cells (Agilent). Successful mutagenesis was verified by Plasmidsaurus whole plasmid sequencing. All plasmids are listed in detail in **Table S2**.

**Protein expression and purification**

As previously described (2), proteins were recombinantly expressed and purified from BL21(DE3) RIPL *E. coli* (Agilent) cell transformants using amylose affinity chromatography and were further purified via size exclusion chromatography (SEC) (**Figure S1**).

**NuPAGE analysis for polyphosphate modification detection**

Protein overexpression cell lysates were treated with polyP (concentration indicated and stated in terms of Pi monomers using the sodium polyphosphate formula weight of 102 g mol^-1^, regardless of chain length) (10.2 mg polyP_700_ was dissolved in 1 mL of MilliQ water for a 100 mM stock) (Kerafast, Cat#EUI003 and Cat#EUI005). Samples were incubated with polyP for 30 min at room temperature (20-25 °C) prior to denaturing with 2X Laemmli Buffer at a 1:1 ratio and boiled at 95 °C for 5 min. Protein samples were then loaded onto a precast 4-12% Bis-Tris NuPAGE gels (ThermoFisher Scientific, Cat#NP0336BOX) and run at 200 V for 45 min in ice-cold 1X NuPAGE running buffer (ThermoFisher Scientific, Cat#NP0001). Alternatively, a homemade equivalent (50 mM Tris-HCl, 50 mM MOPS, 1 mM EDTA, 5 mM sodium bisulfite and 0.1% SDS) running buffer can be used.

**Western blotting**

Protein samples were resolved on a NuPAGE gel then transferred to a PVDF membrane using a wet-transfer system for 2 h at 80 V. Total protein and transfer efficiency were assessed using Ponceau S Staining Solution (ThermoFisher Scientific, Cat#A40000278). Membranes were washed in 1X Tris-buffered saline (TBS) (20 mM Tris-HCl, 150 mM NaCl, pH 7.4) then blocked in 5% milk powder in TBS, 0.05% Tween-20 (TBST) for 1 h rocking at 4 °C. Mouse anti-MBP (1/1,000) (Cell Signaling Technology, Cat#2396S; RRID:AB_2140060) and goat anti-mouse DyLight680 (1/10,000) (Invitrogen, Cat#35518; RRID:AB_614942) were used as primary and secondary antibodies, respectively.

***P. aeruginosa* growth conditions and transformation**

*P. aeruginosa* UCBPP-PA14 and a quadruple knockout strain, *∆ppk1∆ppk2A∆ppk2B∆ppk2C* (referred to as *∆polyP*) were grown in lysogeny broth (LB) in a shaker at 100 rcf at 37 °C. WT or *∆polyP* PA14 cells from overnight culture (1350 µL per transformation) were pelleted via centrifugation at 16,000 rcf for 2 min, then the supernatant was discarded. Cells were washed in 300 µL of sterile 0.3 M sucrose at room temperature and re-pelleted twice, decanting the supernatant each time. The cells were resuspended a third time in 80 µL of 0.3 M sucrose prior to adding 60 ng of plasmid DNA. The sample was then transferred to a pre-chilled electroporation cuvette and electroporated at a peak discharge voltage of 2500 V with the electroporation system set to prokaryote mode with a 5 ms decay. The electroporated sample was mixed with 1 mL of fresh LB and incubated for 2 h in a shaker (100 rcf) at 37 °C. The cell mixture was serially diluted from 1/10 to 1/100,000 with LB and then plated onto appropriate selection LB agar plates (300 µg/mL carbenicillin for pHERD20T).

**Biofilm formation assay**

Biofilm quantification was performed as previously described (3). Biofilms were formed in WT and *∆polyP* PA14 strains recombinantly expressing wild-type or ∆K mutants of EngA and SrmB using the pHERD20T expression vector. *P. aeruginosa* cultures (8 mL) were grown overnight for 16 h shaking at 37 °C in 2% w/vol LB (Lennox; Bioshop) supplemented with the appropriate selection antibiotic (200 µg/mL carbenicillin for pHERD20T). Protein expression was induced by L-arabinose (1% wt/vol). Overnight culture was then diluted 100-fold in M63 minimal media (**Table S3**), plated in a 96-well tissue culture plate (U-bottom, Sarstedt) and grown statically for 24 h at 37 °C. Wells were rinsed twice with sterile water to remove planktonic cells prior to staining the adhered biofilms with a 0.1% wt/vol Crystal Violet solution for 10 min. Unbound dye was removed with three sterile water washes, then the remaining dyed biofilm rings were redissolved in 30% acetic acid and quantified by measuring absorbance at 570 nm.

**Pyoverdine and pyocyanin quantification**

*P. aeruginosa* cultures (50 mL) expressing the poly-lysine proteins (as previously described) were grown in a shaker at 100 rcf for 16 h at 37 °C. Cells were pelleted via centrifugation at 39,000 rcf for 30 min at 4 °C. The supernatant was then clarified with a 0.2 µm filter. The relative production of pyoverdine was quantified by measuring the absorbance of clarified culture at 403 nm (4). Pyocyanin was chloroform-extracted by resuspending 30% v/v chloroform in clarified culture, discarding the pyocyanin-free aqueous layer. The blue pyocyanin-containing organic layer was then mixed with 0.2 M HCl at a 1:1 ratio to re-extract pyocyanin into the aqueous phase, yielding a red product that was quantified spectrophotometrically at 520 nm (4). Spectrophotometric readings were blanked with cell-free culture medium.

**Circular Dichroism spectroscopy**

Recombinantly purified protein samples (1.5 mg/mL) from SEC were dialyzed in lysis buffer (50 mM Tris, 250 mM NaCl, 5 mM BME, 5% glycerol, pH 7.0) and used for circular dichroism spectroscopy (CD) studies (**Figure S2**). CD was conducted using a Chirascan V100 spectrophotometer device (Applied Photophysics Ltd.). A disc cuvette (Hellma Usa Inc., round cylindrical cuvette, 1 mm pathlength) was used to collect spectra data between 190 and 260 nm.

**SYPRO-Orange thermal shift assay**

Invitrogen™ SYPRO™ Protein Gel Stain (Cat. S6650) was purchased from Thermo Fisher Scientific. Triplicate 25 µL reaction mixtures were prepared by combining 2.5 µL of a 50X SYPRO™ Orange stain with 22.5 µL of a 0.5 mg/mL protein sample in a buffer containing 50 mM Tris, 150 mM NaCl, 5 mM β-mercaptoethanol, and 5% glycerol (pH 8.0 for WT SrmB and pH 7.4 for all others). These mixtures were then dispensed into a 96-well qPCR microplate, and triplicate negative controls (lacking protein) were also prepared. Fluorescence was measured at 488 nm using the Bio-Rad CFX Connect™ Real Time System with a Hard-Shell® PCR 96-well, tin-wall plate. The melt curve was generated by increasing the temperature from 10 °C to 95 °C in increments of 0.5 °C with a 10-second hold at each step, and the melt peak was determined by taking the derivative of the melt curve.

The measured melting temperatures (Tm) were 49.9 ± 0.1 °C for wild-type EngA and 47.5 ± 0.2 °C for the ΔK EngA variant, and 48.1 ± 0.1 °C for wild-type SrmB and 50.0 ± 0.2 °C for the ΔK SrmB variant (n = 3, with errors representing standard deviations). These results indicate no significant difference between the corresponding WT and the mutant, and that the Lys deletion does not significantly affect the overall protein structure.

**References**

1. Watson, J. F., and García-Nafría, J. (2019) In vivo DNA assembly using common laboratory bacteria: A re-emerging tool to simplify molecular cloning. *J. Biol. Chem*. **294**, 15271

2. Lehotsky, K., Neville, N., and Jia, Z. (2024) Protocol for detecting histidine polyphosphate modification of human proteins via MBP-tagged expression in E. coli. *STAR Protoc*. **5**, 102947

3. O’Toole, G. A. (2011) Microtiter dish biofilm formation assay. *J Vis Exp*. 10.3791/2437

4. Yeom, J., and Park, W. (2012) Pleiotropic effects of the mioC mutation on the physiology of Pseudomonas aeruginosa PAO1. *FEMS Microbiol Lett*. **335**, 47–57

**
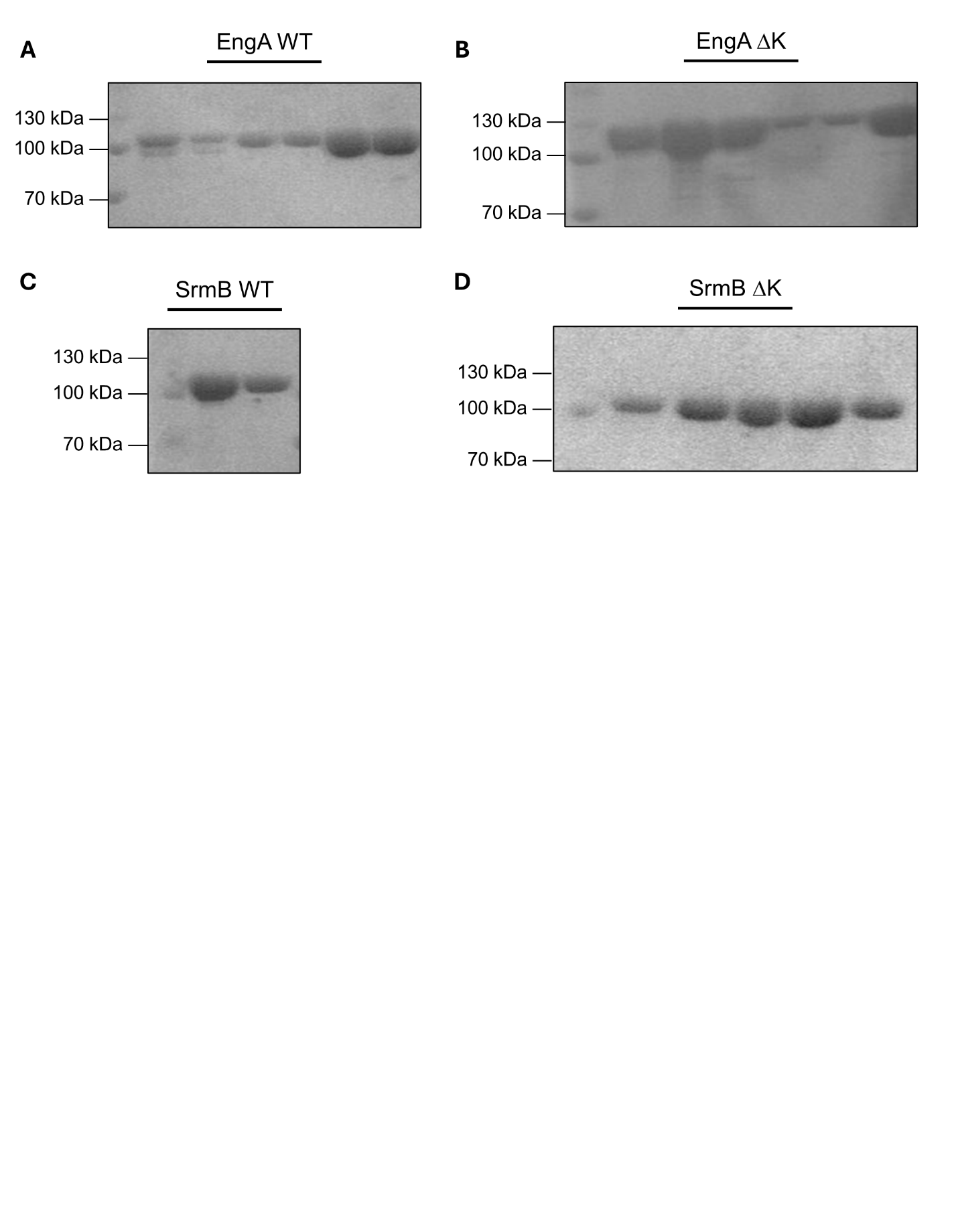
**

**Figure S1. Recombinant purification of EngA and SrmB, as well as their Lys deletion mutants, from BL21(DE3) RIPL *E. coli* cells.** Purified WT EngA **(A)** , ∆K EngA **(B)**, WT SrmB **(C)**, and ∆K SrmB **(D)** protein samples were resolved on SDS PAGE and stained with Coomassie. Proteins were recombinantly purified via amylose affinity chromatography and subsequent purification via SEC. SDS PAGE gel images depict elution fractions from SEC.

**
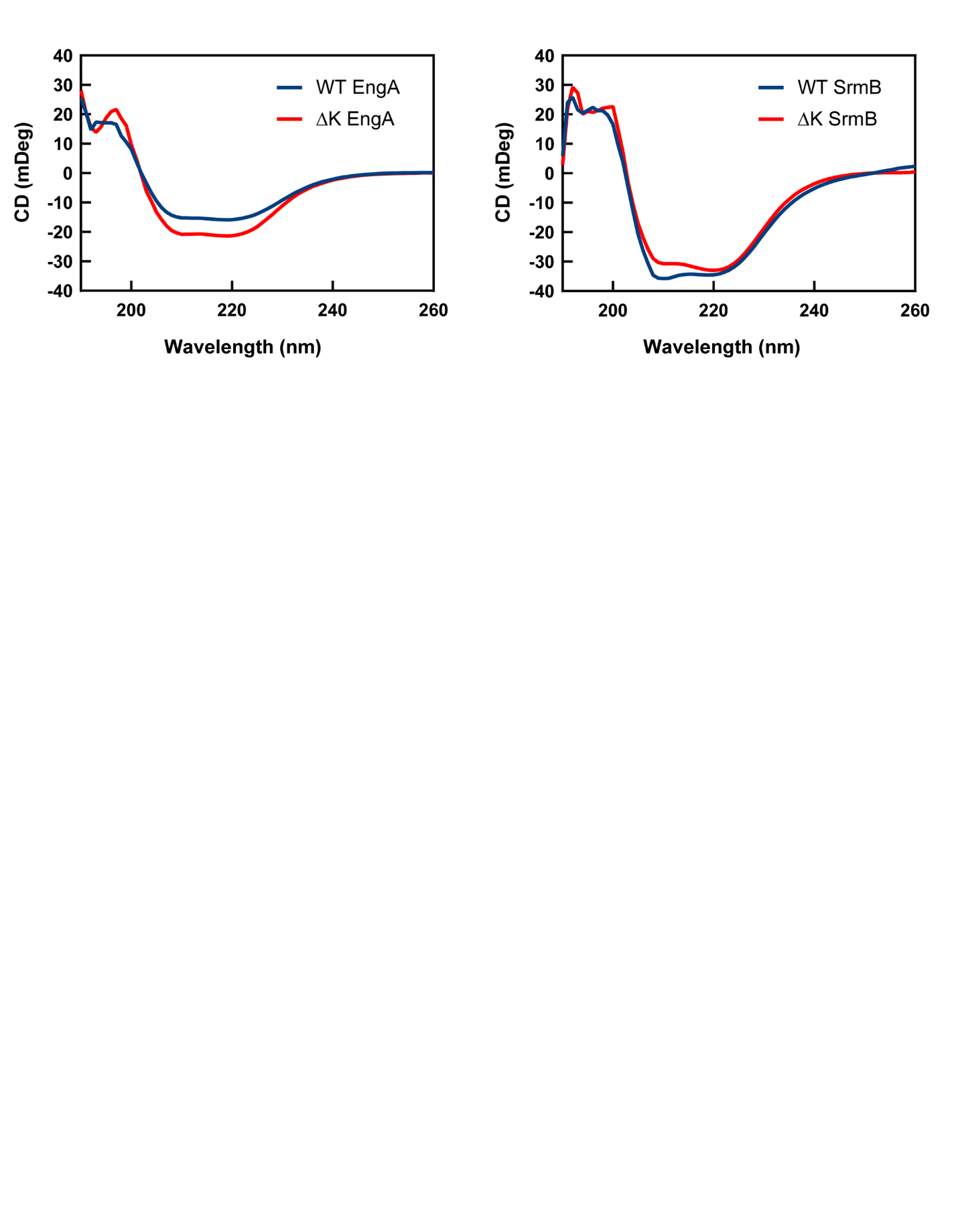
**

**Figure S2. Lysine deletions of EngA and SrmB do not compromise secondary structural elements.** CD spectra of recombinantly purified WT EngA and WT SrmB compared to ∆K EngA and ∆K SrmB, respectively.

**Table S1. Oligonucleotide primers used in this study.**

| **Name** | **Description** | **Sequence (5’ to 3’)** |
| --- | --- | --- |
| EngA_F | Cloning *P. aeruginosa* EngA into HT25 | atatggatccATGGTTCCCGTTATTGCCCTGG |
| EngA_R |  | atatgcggccgcTCAGCGGCGCTTGTCCTTC |
| InfC_F | Cloning *P. aeruginosa* InfC into HT25 | atatggatccATGAGACAGGATAAGCGAGCTCAAC |
| InfC_R |  | atatctcgagTTACTTTTTCTTCTTGGGAGCGATG |
| SrmB_F | Cloning *P. aeruginosa* SrmB into HT25 | atatggatccATGCCGAGGTCTCCCTTGTTCAG |
| SrmB_R |  | atatgaattcTCACTCCGCCGTCGGCTTC |
| FadB_F | Cloning *P. aeruginosa* FadB into HT25 | atatggatccATGATTTACCAAGGTAAAGCCATCAC |
| FadB_R |  | atatctcgagTTAACCGAAGAATTTCTGACCGTT |
| EngA_F | Cloning *P. aeruginosa* EngA into pHERD20T | atatCCATGGGAGACTATAAGGACGACGACGACAAGATGGTTCCCGTTATTGCCCTGG |
| EngA_R |  | atatAAGCTTCTAGCGGCGCTTGTCCTTCTTCTTC |
| SrmB_F | Cloning *P. aeruginosa* SrmB into HT25 | atatGAATTCGGACTATAAGGACGACGACGACAAGATGCCGAGGTCTCCCTTGTTCAG |
| SrmB_R |  | atatAAGCTTCTACTCCGCCGTCGGCTTCTTG |
| EngA_TruncF | Truncates *P. aeruginosa* EngA to remove residues 473 to 493. | atatCCATGGGAGACTATAAGGACGACGACGACAAGATGGTTCCCGTTATTGCCCTGG |
| EngA_TruncR |  | atatAAGCTTCTAGTTGACCTGGCGCGCGGTCAG |
| SrmB_TruncF | Truncates *P. aeruginosa* SrmB to remove residues 387 to 446. | atatGAATTCGGACTATAAGGACGACGACGACAAGATGCCGAGGTCTCCCTTGTTCAG |
| SrmB_TruncR |  | atatAAGCTTCTACGGCCCCTGGTAGGCGGC |

**Table S2. Plasmids used in this study.**

| **Storage Name** | **Insert Gene** | **Tag** | **Backbone vector** | **Description** | **Source** |
| --- | --- | --- | --- | --- | --- |
| NN316 | SrmB | N-MBP-insert-C | pET16b | PA14_19290 | This study cloned off *P. aeruginosa* UCBPP-PA14 genomic DNA |
| NN315 | InfC | N-MBP-insert-C | pET16b | PA14_28660 |  |
| NN314 | EngA/YfgK | N-MBP-insert-C | pET16b | PA14_14930 |  |
| NN317 | FadB | N-MBP-insert-C | pET16b | PA14_25080 |  |
| NN320 | SrmB (1-386) ∆K | N-MBP-insert-C | pET16b | PA14_19290 with residues 387-446 deleted |  |
| NN321 | EngA (1-472) ∆K | N-MBP-insert-C | pET16b | PA14_14930 with residues 473-493 deleted |  |
| NN322 | EngA | N-FLAG-insert-C | pHERD20T | PA14_14930 |  |
| NN323 | EngA (1-472) ∆K | N-FLAG-insert-C | pHERD20T | PA14_14930 with residues 473-493 deleted |  |
| NN324 | SrmB | N-FLAG-insert-C | pHERD20T | PA14_19290 |  |
| NN325 | SrmB (1-386) ∆K | N-FLAG-insert-C | pHERD20T | PA14_19290 with residues 387-446 deleted |  |
| NN188 | N/A | N-FLAG-insert-C | pHERD20T | Empty vector for expressing FLAG alone | Dr. Florian Schubot. |

**Table S3. M63 biofilm medium**

| **Reagent** | **Final Concentration** | **Amount** |
| --- | --- | --- |
| Ammonium sulfate | 15 mM | 0.5 g |
| Potassium phosphate, monobasic | 100 mM | 3.4 g |
| Ferrous sulfate, heptahydrate (1 mM) | 1.8 µM | 450 µL |
| Total | N/A | 240 mL |
| **Adjust pH to 7.0 with KOH. Autoclave. Let cool, then add:** | | |
| Magnesium sulfate | 1 mM | 0.25 mL |
| Arginine (10%) | 0.4% | 10 mL |
| **Total** | **N/A** | **250 mL** |

**Table S4. A list of *Pseudomonas aeruginosa* PA14 proteins containing at least three lysine residues within a 20-amino acid window (only the 20-aa sequences are shown).**

>PA14_07530 | Max Lysine: 9

KSWNKDKKPWDGKKWDGKKK

>PA14_12760 | Max Lysine: 9

KKPKKPKKPKAAESAGKPGK

>PA14_14930 | Max Lysine: 9

NKKRRLMSHHKKAEKKKKDK

>PA14_19290 | Max Lysine: 9

KKLKASGKAAGSKKKKQDRK

>PA14_51730 | Max Lysine: 9

AKKKAADDAKKKAAEDAKKK

>PA14_06000 | Max Lysine: 8

KPAAKKGAAAKGKADKPKTK

>PA14_65200 | Max Lysine: 8

KDKGGKKGKVVPAGKSAKGK

>PA14_72970 | Max Lysine: 8

KPEPPKPKPVEKPKPKPKPK

>PA14_01200 | Max Lysine: 7

AGKKTKAEKCADDGCKKARK

>PA14_09180 | Max Lysine: 7

RKKIAVPAKRTPRDKKKLLK

>PA14_15960 | Max Lysine: 7

EQNLDRDKAEKLAKKIKKGK

>PA14_25080 | Max Lysine: 7

NDQELKKKAKKYDEVAKDVK

>PA14_28660 | Max Lysine: 7

YGKHLFEKKKQAAVAKKNQK

>PA14_28670 | Max Lysine: 7

KMKTKSGAAKRFKKTAGGLK

>PA14_40840 | Max Lysine: 7

KASLKALRKEESKAAKQAKK

>PA14_54370 | Max Lysine: 7

DVSRKRKLLEKQKAGKKRMK

>PA14_58920 | Max Lysine: 7

NKQIEHAKEKRDRYEKKAKK

>PA14_60550 | Max Lysine: 7

KDKPAEKETVAAKDKAAAKK

>PA14_63060 | Max Lysine: 7

KKHLVKCEIALAKGKKDFDK

>PA14_66790 | Max Lysine: 7

SGMSKGKKKTRKLKVAEALK

>PA14_66875 | Max Lysine: 7

AKPAAKPAAKPAAKKPAAKK

>PA14_70590 | Max Lysine: 7

IWFQGLVKYLKPKKKAPEKK

>PA14_01020 | Max Lysine: 6

KIRVLNISKPELHKTLKKFK

>PA14_01290 | Max Lysine: 6

AKRKEEAAKVKELTSKEWTK

>PA14_01560 | Max Lysine: 6

IKDLKVKGSSLVVKVGTKVK

>PA14_02490 | Max Lysine: 6

ENAVKPPPPKPVPKPKPKPK

>PA14_03920 | Max Lysine: 6

KQIKAGVYQKLDKSKLPNWK

>PA14_04220 | Max Lysine: 6

KLCSPKALALRSKVWTKLKK

>PA14_04900 | Max Lysine: 6

DKKAPQGGEKKGLFGWWRKK

>PA14_05480 | Max Lysine: 6

VKQIKTIKTKSKVSSIVQAK

>PA14_07040 | Max Lysine: 6

EKDLRKAMGKGDPEKIEKRK

>PA14_07480 | Max Lysine: 6

GKLLIKPSQKNAKAFYGKVK

>PA14_07520 | Max Lysine: 6

VSEQLDKAKKALKKHGRGSK

>PA14_07560 | Max Lysine: 6

KPTAERKRKAAAAVKRHAKK

>PA14_08240 | Max Lysine: 6

KPKVDDKAGKGQAKAGDDEK

>PA14_08870 | Max Lysine: 6

VKGKTKRTARGLGKRNDWKK

>PA14_09000 | Max Lysine: 6

KGKGVRYADEVVRRKEAKKK

>PA14_11590 | Max Lysine: 6

QLPKASPVKKSKPAKPSSPK

>PA14_14040 | Max Lysine: 6

KAEKGEKPAKPAAKEGRDAK

>PA14_14220 | Max Lysine: 6

RNNKQSKQYISFIKGKGGKK

>PA14_14470 | Max Lysine: 6

YKSQKAEPLKLKKLTLLADK

>PA14_15350 | Max Lysine: 6

EAKKMIAAGKSPAKEKARDK

>PA14_17170 | Max Lysine: 6

DMLKKLKPKLDQAVEETIKK

>PA14_19410 | Max Lysine: 6

KFWFHLSKKQLKERLKALEK

>PA14_20520 | Max Lysine: 6

GKNAHAKALKGLLKVIKRTK

>PA14_21880 | Max Lysine: 6

WRKKVKDEVLRLKITGKDDK

>PA14_22220 | Max Lysine: 6

EPKCPKKSQEEFVKWKQRQK

>PA14_24020 | Max Lysine: 6

KKPTGNPQPKNWNKDGYLKK

>PA14_41210 | Max Lysine: 6

KPIKIAAAKIPGFKAGKALK

>PA14_43850 | Max Lysine: 6

KKAQEEVAKSKEGLIERLKK

>PA14_44670 | Max Lysine: 6

EPVEPRKGKSKGRKEKEREK

>PA14_46520 | Max Lysine: 6

KGNVAYDKSKVVYKKGAGDK

>PA14_46550 | Max Lysine: 6

SGAHCAEKAKEIENLKKKGK

>PA14_49860 | Max Lysine: 6

KGLNAKKETRRKPLKTAQEK

>PA14_50420 | Max Lysine: 6

EKTQASKKANSDIQKDSKTK

>PA14_51810 | Max Lysine: 6

HSKWANIKHRKERQDAKKGK

>PA14_52140 | Max Lysine: 6

LKAGLVNEKQAKQATKQKQK

>PA14_53620 | Max Lysine: 6

MKKEPKGAKNRIVIAKDEKN

>PA14_54890 | Max Lysine: 6

TVEWFARKFYKSGQKMKRKK

>PA14_56990 | Max Lysine: 6

DQAKLAGKKPKALERYLRKK

>PA14_57130 | Max Lysine: 6

GIDKDLAKKIVGLIKERKLK

>PA14_58190 | Max Lysine: 6

MKASKGKANPQQVNELLKKK

>PA14_58760 | Max Lysine: 6

NPLKVRKKGITLFGAGKKIK

>PA14_59220 | Max Lysine: 6

TKEKKEINKKVSDQVDGLLK

>PA14_59380 | Max Lysine: 6

REVQKPKRARKPKPAKATAK

>PA14_62760 | Max Lysine: 6

KVGGSKTVSVEVRKKKTYVK

>PA14_62970 | Max Lysine: 6

KGQKMAPPQISAEVLKKMKK

>PA14_65080 | Max Lysine: 6

QPWSKGQQNKAAAFAKGKKK

>PA14_65660 | Max Lysine: 6

KAGKKVERKKITQGPALPGK

>PA14_67630 | Max Lysine: 6

QRADLLKKLKQLRDVTKKGK

>PA14_68810 | Max Lysine: 6

EKNKLDLKKLDDKCWPAALK

>PA14_69370 | Max Lysine: 6

EKLLAKLEKQRGKAQEKLHK

>PA14_70180 | Max Lysine: 6

HFYTTDKNKRTKPEKIEIKK

>PA14_00060 | Max Lysine: 5

SQPKLALKQLAKQGDECLKK

>PA14_00080 | Max Lysine: 5

KLEELIKKIGPPKWPWAVDK

>PA14_00120 | Max Lysine: 5

KPIIFYRPPKLKAVDELLKK

>PA14_00510 | Max Lysine: 5

KLELLAANDSNYYLYDKKKK

>PA14_00800 | Max Lysine: 5

KPANPKVQSVQFFPNPKLKK

>PA14_00820 | Max Lysine: 5

ASTTKGGSKPKTGSNPKVPK

>PA14_01140 | Max Lysine: 5

APKGAPKTQVPKGFEKVYGK

>PA14_01350 | Max Lysine: 5

RYDAKANGKKLLKDVQLLLK

>PA14_01970 | Max Lysine: 5

PVIAVHILPKTLKHKSEQKK

>PA14_03700 | Max Lysine: 5

AKKQYGSDEKAKDYVQALYK

>PA14_03930 | Max Lysine: 5

KQIQAGAFQKLDKSKLPNWK

>PA14_04930 | Max Lysine: 5

KVATTKAQRKLFFNLRSQKK

>PA14_06030 | Max Lysine: 5

PFMKRYSKAFLEKHPELKGK

>PA14_06500 | Max Lysine: 5

KHPSAKDMPYVLEMVKGVKK

>PA14_06750 | Max Lysine: 5

KTLKLKAVVKDPRLITPTGK

>PA14_06980 | Max Lysine: 5

EKWKLGQKKVDDGVLLLVAK

>PA14_07290 | Max Lysine: 5

RVQGGKLKAKKEIRVASLFK

>PA14_07870 | Max Lysine: 5

ADFWDIKKFPGKRGLRKGAK

>PA14_08360 | Max Lysine: 5

RAKRKEPAVIAEIKKASPSK

>PA14_08540 | Max Lysine: 5

SSKDAKRFTHLKVGQEVKLK

>PA14_08730 | Max Lysine: 5

MAKLTKRQKAIAEKVVAGKQ

>PA14_08760 | Max Lysine: 5

KKRKLQQGDDLAPGVLKIVK

>PA14_08780 | Max Lysine: 5

KIFGPVKDYECLCGKYKRLK

>PA14_08790 | Max Lysine: 5

TSGVKDRKQGRSKYGAKRPK

>PA14_08860 | Max Lysine: 5

KAQKTRSEVSGGGKKPWRQK

>PA14_08880 | Max Lysine: 5

KGKKTRSNKRTDNMIVRRRK

>PA14_08890 | Max Lysine: 5

KKGPFIDLHLLKKVEVAVEK

>PA14_08920 | Max Lysine: 5

VKRGGKIWIRVFPDKPVTKK

>PA14_08950 | Max Lysine: 5

IGDIIKVTVKEAIPRGKVKK

>PA14_08960 | Max Lysine: 5

DDEVIVIAGKDKGKRGKVLK

>PA14_08980 | Max Lysine: 5

KESMKNRELKRQLTVAKYAK

>PA14_08990 | Max Lysine: 5

KTVVSMPSSKLKAAVAKVLK

>PA14_09090 | Max Lysine: 5

MAKPAARPRKKVKKTVVDGI

>PA14_09970 | Max Lysine: 5

AKYKHDSNKANEGKPFDAAK

>PA14_11690 | Max Lysine: 5

EQIKHFFENYKDLEKGKWVK

>PA14_12100 | Max Lysine: 5

KKGTELTKGLVWKGSEHEVK

>PA14_12180 | Max Lysine: 5

MAKKSKRPNKAKSLVAQPLF

>PA14_13010 | Max Lysine: 5

LKKLSAALTSPEVKAFIEKK

>PA14_13140 | Max Lysine: 5

GKKEIYIPYNSYKLDDPKIK

>PA14_13410 | Max Lysine: 5

SGKYEKGMKMRHVRLGKDVK

>PA14_13580 | Max Lysine: 5

KARCKEKARELMSMVKLEPK

>PA14_13800 | Max Lysine: 5

ISGCPYKKIYFNWKSGKSEK

>PA14_15400 | Max Lysine: 5

KKRRQKARKALAELAAVGWK

>PA14_15720 | Max Lysine: 5

ARKMAEKEGLNPNKWLDVKK

>PA14_15840 | Max Lysine: 5

GKVDKVLVLKSERKLLLLNK

>PA14_16000 | Max Lysine: 5

KAKLYYLRALSGKAARIKEK

>PA14_16860 | Max Lysine: 5

GATKKKESIFDLFKVVGALK

>PA14_17260 | Max Lysine: 5

GKKKPEEMAKQRGGFIEGCK

>PA14_17710 | Max Lysine: 5

ICKSNPRFKCVQGRPKPKIK

>PA14_18480 | Max Lysine: 5

KKQFESKRVGLLSKLGTFHK

>PA14_18590 | Max Lysine: 5

TEFKDLKNGQYKIISFYAKK

>PA14_18740 | Max Lysine: 5

KLMDYAEKHGIPIERHGKKK

>PA14_18920 | Max Lysine: 5

LKRLKIEASMARVALKKAEK

>PA14_19810 | Max Lysine: 5

FKKGETFSKIFKGIHDLELK

>PA14_20230 | Max Lysine: 5

TYHDQDKCPPLVNKRKKRAK

>PA14_20320 | Max Lysine: 5

QKNKLRDFFFKYGANAEQKK

>PA14_20870 | Max Lysine: 5

YRVVKEKKLNTLVIHATGKK

>PA14_21400 | Max Lysine: 5

LKLPFGRPAKAEARKPEAKK

>PA14_21410 | Max Lysine: 5

RVEQDLTQALKQSLSKKKAK

>PA14_21470 | Max Lysine: 5

IPKKWNPNLKAVVEWEKDPK

>PA14_21490 | Max Lysine: 5

NGEKKRRAALEPKLKELEEK

>PA14_21570 | Max Lysine: 5

AEKAKEGSCGEAKCGADKGK

>PA14_21610 | Max Lysine: 5

EYLARKLKADDNAYKDIKAK

>PA14_21790 | Max Lysine: 5

ALKEKVDEIEAQQMRKVYKK

>PA14_21820 | Max Lysine: 5

DDLDSKAVAKGIEDALGKKK

>PA14_22090 | Max Lysine: 5

TTRKPSTQKQRTCKAKGCSK

>PA14_22160 | Max Lysine: 5

REALKGFLEKEMSKLLGKMK

>PA14_22260 | Max Lysine: 5

KQAFPSAKEYAKAVAIGAKK

>PA14_22270 | Max Lysine: 5

DVDGKRFVYEGANRKKKVYK

>PA14_22330 | Max Lysine: 5

WIPHWMFAKWKLKFLDDPKK

>PA14_22740 | Max Lysine: 5

KLPAAEQKALKDELKASHEK

>PA14_22980 | Max Lysine: 5

EKRAAETLKKLVEAKGHTWK

>PA14_23260 | Max Lysine: 5

YQLNKARLIEKIAELVKEKK

>PA14_23330 | Max Lysine: 5

LSVKSKDVDDEKDAMKELRK

>PA14_23410 | Max Lysine: 5

KEHASTSKIKLKFICPEHRK

>PA14_23420 | Max Lysine: 5

YLDKARQQPEKVKMVLEKIK

>PA14_23830 | Max Lysine: 5

VSGEGKASKGADKGGKGDSK

>PA14_23860 | Max Lysine: 5

DRLKFRDSKKYKDRLAAAQK

>PA14_24445 | Max Lysine: 5

EPMKAKARELLTWLGKAKLK

>PA14_24590 | Max Lysine: 5

KHREILKKLRKRELSPPPEK

>PA14_24990 | Max Lysine: 5

KHLNDGQKALLKKSQIAWIK

>PA14_25490 | Max Lysine: 5

LLGQVWKQIKGKQLSAQKLK

>PA14_25600 | Max Lysine: 5

EWKSETPKANDDKSWKLLEK

>PA14_25840 | Max Lysine: 5

KPDYACLKKASEAPKIDYPK

>PA14_25860 | Max Lysine: 5

AGIKVKSVAELVEKLKNEAK

>PA14_26550 | Max Lysine: 5

SKVLTQKSKDQLGELDKSLK

>PA14_26620 | Max Lysine: 5

KKVAPAAEQVAEPKPPAKPK

>PA14_26640 | Max Lysine: 5

KYKKTDLKRDAYDPARVSDK

>PA14_27210 | Max Lysine: 5

QKAEFNKSGRNAAVVKMKLK

>PA14_27830 | Max Lysine: 5

AKARAAHQEKLAKQAEATKK

>PA14_28000 | Max Lysine: 5

LKTPGYKYQQSKCRAIDPKK

>PA14_28680 | Max Lysine: 5

RVKRGVIARRRHKKILKLAK

>PA14_28710 | Max Lysine: 5

KIKKAKLRGVESFGMLCSAK

>PA14_28770 | Max Lysine: 5

PSAHQSQKRLRDKQELLKKK

>PA14_28810 | Max Lysine: 5

MRRAMTIEVKKEDKRKTFLK

>PA14_29050 | Max Lysine: 5

SKASKGDTSMGKKGAPRVEK

>PA14_29090 | Max Lysine: 5

KQIQAGALKKLDKSQLPNWK

>PA14_29130 | Max Lysine: 5

RKVPGLKKKPSTSELVDWLK

>PA14_29590 | Max Lysine: 5

LLEKLKSDSSLKQELEFKDK

>PA14_30050 | Max Lysine: 5

GFIGQQKLISIKKHLKTTNK

>PA14_30150 | Max Lysine: 5

EKPEVRAIAEKHGLATAKKK

>PA14_30180 | Max Lysine: 5

KAGDVRIEFVGKDGKVEVKK

>PA14_30660 | Max Lysine: 5

RMFDADAKLLYVGKAKSLKK

>PA14_31770 | Max Lysine: 5

AKNLKLVDKVGELAAAKGVK

>PA14_32790 | Max Lysine: 5

NDSSYYLYDKKSKGSFGSKK

>PA14_32830 | Max Lysine: 5

CKKKFTGNGKSFLVGALCSK

>PA14_33120 | Max Lysine: 5

LKKPKQSLLRSVLTLGLGKK

>PA14_33310 | Max Lysine: 5

VFPSQELILDKGDKKGQKSK

>PA14_33680 | Max Lysine: 5

SKPTNPAITYAYKGIKAKTK

>PA14_35700 | Max Lysine: 5

KSKGLEYGRLRSGLRKLKQK

>PA14_35720 | Max Lysine: 5

QQLAKGEEEGEKVRAGKAKK

>PA14_36070 | Max Lysine: 5

MGKAVNKPVFKLLGGRTKEK

>PA14_36520 | Max Lysine: 5

KQLGQAMQQHKKELKARLEK

>PA14_36760 | Max Lysine: 5

EYKDSFSDKIMKLVEEKAAK

>PA14_37790 | Max Lysine: 5

KPQRILAKLKKQSDYYNFHK

>PA14_38080 | Max Lysine: 5

DAYRIEQSADKKTLKVSLKK

>PA14_38860 | Max Lysine: 5

LDASVVALNKNTGKVVWKKK

>PA14_39470 | Max Lysine: 5

IPHKVFREFIKKYECQDKAK

>PA14_40390 | Max Lysine: 5

NKGKDNAAAKALVDYLKGAK

>PA14_40490 | Max Lysine: 5

GAVVLAGVLLVKRLLPKKKK

>PA14_40670 | Max Lysine: 5

EAEKGDKPEAKGKILMATVK

>PA14_40690 | Max Lysine: 5

MKFLTPEQMEKPRKRRLRKK

>PA14_41060 | Max Lysine: 5

EWLPNWKKRGWKTASKQPVK

>PA14_41190 | Max Lysine: 5

KVGDEQAKAKIDEIKARLAK

>PA14_41220 | Max Lysine: 5

EVKERILEYLAVQKRVKKLK

>PA14_41570 | Max Lysine: 5

DVKFDFDKSKVKENSYADIK

>PA14_41575 | Max Lysine: 5

EVMLKVLYGLKNFEGKSKFK

>PA14_41640 | Max Lysine: 5

KCFEDNSLVKEQVDKDGKGK

>PA14_41820 | Max Lysine: 5

KLGKGKVETRPIKGTRPRGK

>PA14_41950 | Max Lysine: 5

QQLKKLNMTAHRNTKLKVRK

>PA14_42450 | Max Lysine: 5

QQKKLKLTEIEQARKQNLQK

>PA14_42660 | Max Lysine: 5

SLDSVKPDLKKINPVEGAKK

>PA14_42900 | Max Lysine: 5

DKAAEKGGKLGKAAAAAAGK

>PA14_43100 | Max Lysine: 5

PARKGWKQNNALFKLEALKK

>PA14_43950 | Max Lysine: 5

AGGRGKAGGVKLVKSKEDAK

>PA14_44000 | Max Lysine: 5

VNMKPIMDLRSKYKDLFEKK

>PA14_44070 | Max Lysine: 5

DVSNIDKFVEKAKDKNDPFK

>PA14_44290 | Max Lysine: 5

AGLLAKKAVEKGLQRKPWVK

>PA14_44420 | Max Lysine: 5

RIKLDKAPMSGNKLLRKTAK

>PA14_44480 | Max Lysine: 5

QELRPMVKMIRELKKKIEEK

>PA14_44920 | Max Lysine: 5

LEKEWRTQKHKSKGNMFLRK

>PA14_45110 | Max Lysine: 5

VLKNGGDENKAKEFVGKLFK

>PA14_45720 | Max Lysine: 5

KQEVRDEYKDSEGKPEVKSK

>PA14_45810 | Max Lysine: 5

KKEATPADAGAPSGGKSKLK

>PA14_45830 | Max Lysine: 5

KPKAAAPKSQQKTPEPSNDK

>PA14_45940 | Max Lysine: 5

DKKLLGEMHKLRAQVFKERK

>PA14_46460 | Max Lysine: 5

AAPFGKPQAMPPSKRKADKK

>PA14_46910 | Max Lysine: 5

TRLLSKKTANIKDFDDLKGK

>PA14_48880 | Max Lysine: 5

WLADVEPIKGKRFRKRFKTK

>PA14_48930 | Max Lysine: 5

DAIATWEQKVEQAKKELKDK

>PA14_49250 | Max Lysine: 5

LEKKAKNADKANTWSDIDFK

>PA14_49270 | Max Lysine: 5

HKWTDKIARKMQASKEVWGK

>PA14_49460 | Max Lysine: 5

ASYGKLKLHKVVQAKDLWRK

>PA14_49480 | Max Lysine: 5

ITKLTLHRFKKYRDKEISIK

>PA14_49710 | Max Lysine: 5

DEAVKSIYAKYLPKLKAPQK

>PA14_49930 | Max Lysine: 5

RLFGVSKGTFKKAIGGLYKK

>PA14_50950 | Max Lysine: 5

LKAVGRNDPCPCGSEKKFKK

>PA14_51290 | Max Lysine: 5

KDEAVCQLFDVIKLKKLYGK

>PA14_51530 | Max Lysine: 5

NKKLKLFQHISSEIGASLKK

>PA14_51640 | Max Lysine: 5

KTSKNLNIWTIKVSGPRKTK

>PA14_51690 | Max Lysine: 5

KEKLYYDAAFDLIKSKDFDK

>PA14_52420 | Max Lysine: 5

KVLKAMKRPAFEDKTLARIK

>PA14_52660 | Max Lysine: 5

KEAAKQGLAKMKALMEMGFK

>PA14_52790 | Max Lysine: 5

FVMKEGASLNDPKADLKGKK

>PA14_53030 | Max Lysine: 5

QKRRLDGKAKRGAVKAGRGK

>PA14_53180 | Max Lysine: 5

HKESYCDEQPFYRTKVKLKK

>PA14_53200 | Max Lysine: 5

KLMADMDKELGVAAPAQKKK

>PA14_53590 | Max Lysine: 5

FKDKFQPAKPSTALKGIARK

>PA14_54210 | Max Lysine: 5

WGVYGKDKLDLKHARKVLDK

>PA14_54390 | Max Lysine: 5

LKIEAKNLPTLKLGDSNKLK

>PA14_54870 | Max Lysine: 5

IAELAKAVGMNQCKLKKCFK

>PA14_55770 | Max Lysine: 5

AKKVGKLSDLPAREKADLEK

>PA14_56070 | Max Lysine: 5

AIKELQERLKSLEQDDKLKK

>PA14_56130 | Max Lysine: 5

RFGDKTEKKDSRKEDEERLK

>PA14_57070 | Max Lysine: 5

LDVKLDSKRNDSDKDEWKLK

>PA14_57220 | Max Lysine: 5

PLLKKLFGSKNERDVKRMAK

>PA14_57580 | Max Lysine: 5

EVERKKVGLRKARKRPQYSK

>PA14_57670 | Max Lysine: 5

LRSLREPLKSTESGKKKAAK

>PA14_57940 | Max Lysine: 5

AIIKKLVAAENAKKPLSDSK

>PA14_58350 | Max Lysine: 5

KDAQIRFKGNKDYWKPDEVK

>PA14_58390 | Max Lysine: 5

KDAQIRFKGNKDYWKPEDVK

>PA14_58550 | Max Lysine: 5

FDSLYKIRLQGKRLKKRNRK

>PA14_58960 | Max Lysine: 5

MDKQKVLAKVEKLMALANAK

>PA14_59050 | Max Lysine: 5

RNNKQSKQYISFIKGKGGRK

>PA14_59180 | Max Lysine: 5

KKPLRFGAKGKLLNVPGWRK

>PA14_59400 | Max Lysine: 5

GAQAEKLVRRCKEAVEAKKK

>PA14_59530 | Max Lysine: 5

SKSGRILVVKGDTYKDKVRK

>PA14_59640 | Max Lysine: 5

PLIVPAPKRPIPAKPKPKPK

>PA14_60070 | Max Lysine: 5

KPFELEDDDEAEPSGKKKSK

>PA14_60130 | Max Lysine: 5

KTSKNLNIWTIKVSGPRKTK

>PA14_60310 | Max Lysine: 5

DNDKAALLLIDLKKGTLIKK

>PA14_60530 | Max Lysine: 5

EGKEKLNWLGKMDSGSLKNK

>PA14_60700 | Max Lysine: 5

DLYLKGDDKALDAQQKKGLK

>PA14_61080 | Max Lysine: 5

PKGKGALLFKKLAEERLPGK

>PA14_61850 | Max Lysine: 5

DKSIKYDSKTLAVLQQDTKK

>PA14_62160 | Max Lysine: 5

PKDMGDPTQKFEYSYPKKVK

>PA14_62170 | Max Lysine: 5

KPADPEQKAIDDKVKQQVAK

>PA14_62650 | Max Lysine: 5

KVAEGTADKVAKQETSKAPK

>PA14_62680 | Max Lysine: 5

MNSDVIKGKWKQLTGKIKER

>PA14_62860 | Max Lysine: 5

KDKIMMGAERKTMVMSEKEK

>PA14_63800 | Max Lysine: 5

LPWDKMDKEFLAKPRKWDSK

>PA14_64460 | Max Lysine: 5

KMKSCNADASAKSLKGDERK

>PA14_64490 | Max Lysine: 5

KSKYSDKQQRKAEHIEESYK

>PA14_64900 | Max Lysine: 5

AGDYIVKVLKAKKVAVIHDK

>PA14_65590 | Max Lysine: 5

PATARKPVAKPKAKEEEGPK

>PA14_65605 | Max Lysine: 5

LLKEQKRLQTLLGSEAKLKK

>PA14_66170 | Max Lysine: 5

KEKGKGYYFSPKLIEWLGPK

>PA14_66310 | Max Lysine: 5

KASMEIPSPKAGVVKSIKAK

>PA14_66480 | Max Lysine: 5

ELADLQPKLESKHKINKLRK

>PA14_66560 | Max Lysine: 5

FQFIEVGRKDPKKKLLRQRK

>PA14_66570 | Max Lysine: 5

EVFKYYRKGISKGLMKILSK

>PA14_66640 | Max Lysine: 5

KLRMSILAKTYRYNDKGLKK

>PA14_66760 | Max Lysine: 5

AKKKPAPKRGASRYQAPAAK

>PA14_66880 | Max Lysine: 5

GSDYFKELVKAGEALEKKGK

>PA14_67090 | Max Lysine: 5

NPVLKGWRLTLRIKVKDPKK

>PA14_67130 | Max Lysine: 5

AGKVVERLKSTLGKPELKVK

>PA14_67790 | Max Lysine: 5

LKKLLDGIQQEKSGVQKQLK

>PA14_67840 | Max Lysine: 5

SKIAVKNLKDAARYKLGAYK

>PA14_67975 | Max Lysine: 5

EKLEEKLGDKVSPELKDALK

>PA14_68070 | Max Lysine: 5

KLQFVAPKSVDFKTDKDSLK

>PA14_68460 | Max Lysine: 5

NNLGKDNDSGHRGKKHRKHK

>PA14_68710 | Max Lysine: 5

ETDKLAGELIKKYPGMKLTK

>PA14_69190 | Max Lysine: 5

IDKLKQTKTNDEFFDSMKRK

>PA14_69220 | Max Lysine: 5

LKRKVLKLGDVEKLDLEGIK

>PA14_69230 | Max Lysine: 5

GMGKTLRMKKLLHAPFTLKK

>PA14_69280 | Max Lysine: 5

ADACPKAAKELVCKFALKRK

>PA14_69660 | Max Lysine: 5

GQKGPLYLPDDEKAKKEHSK

>PA14_69770 | Max Lysine: 5

KKAKAAVVEEELPSVEAKQK

>PA14_70270 | Max Lysine: 5

YGGRPVMWKTGHSLIKKKMK

>PA14_70740 | Max Lysine: 5

KPLYTFIQDKKPGDKNGDGK

>PA14_70770 | Max Lysine: 5

LRIIVKKMRGPKIVLAKVVK

>PA14_71310 | Max Lysine: 5

RPRPKRLKVIKAKTGAERMK

>PA14_71510 | Max Lysine: 5

LHKLAGKTAAKAGIVLREKK

>PA14_72450 | Max Lysine: 5

KFLSTYNSFAIKGQMEKAKK

>PA14_72490 | Max Lysine: 5

RALKPLLEDPKKLKVGQHAK

>PA14_72640 | Max Lysine: 5

LDKAGVIKLKDNKSITATPK

>PA14_72960 | Max Lysine: 5

SKKKEEPKESLLRTLLRHPK

>PA14_73220 | Max Lysine: 5

FGRARQKNLENWKRPEKIKK

>PA14_73250 | Max Lysine: 5

KSTQKITNAMEKVAVSKMRK

>PA14_73410 | Max Lysine: 5

FTFHRGLKADCSDKEKAQKK

>PA14_00010 | Max Lysine: 4

KTHLMHAVGNHLLKKNPNAK

>PA14_00050 | Max Lysine: 4

TAIISVKVPDPKFSSQTKDK

>PA14_00090 | Max Lysine: 4

LGFAKKCGVDLQQIDKSGPK

>PA14_00180 | Max Lysine: 4

HAAIGETVGCADKLKKGWAK

>PA14_00200 | Max Lysine: 4

NGKLFVDYLSTLKRDRIRKK

>PA14_00430 | Max Lysine: 4

PKIDGLEVIARLKSLKLDTK

>PA14_00480 | Max Lysine: 4

SAFAAKPCEELKAEIDAKIK

>PA14_00690 | Max Lysine: 4

PLKGDQLKLEGHALKVVDLK

>PA14_00710 | Max Lysine: 4

GVDMKKTASAVWQGGLKDGK

>PA14_00910 | Max Lysine: 4

KGDRLVAAQVKKAADGTVDK

>PA14_01010 | Max Lysine: 4

MKAIKDPALLQALASAPKPK

>PA14_01030 | Max Lysine: 4

MAVDMFIKIGDVKGESKDKT

>PA14_01100 | Max Lysine: 4

KAISRLQLGRIKKRVEATHK

>PA14_01160 | Max Lysine: 4

DGSGAINIKADKNVVVKGRK

>PA14_01320 | Max Lysine: 4

SFTVTFAHHALKKNKRGPLK

>PA14_01540 | Max Lysine: 4

EKGWLVTVEVKPREVKDWAK

>PA14_01620 | Max Lysine: 4

TSIKMARAYWRLKGQPQKTK

>PA14_01710 | Max Lysine: 4

PAKWKEGEKTLAPSLDLVGK

>PA14_01720 | Max Lysine: 4

YKAKGVCFCPHCDGPLFKGK

>PA14_01730 | Max Lysine: 4

YEKAKHDLQIELLKVQSWVK

>PA14_02380 | Max Lysine: 4

MPAKKHFARPRPYEVTPKVK

>PA14_02460 | Max Lysine: 4

FGGFLVTRRMLEMFKKKAPK

>PA14_02470 | Max Lysine: 4

KNDPKSPIAGMPILEAYKAK

>PA14_02750 | Max Lysine: 4

MRLKLLRKKLGVTLETLAEK

>PA14_02810 | Max Lysine: 4

SKSTLERKESGTFAELFKHK

>PA14_03160 | Max Lysine: 4

RSWHGVVTKIAKQGKGAVVK

>PA14_03170 | Max Lysine: 4

RDIKLKLIQPGKPTQNAFIK

>PA14_03180 | Max Lysine: 4

TVADAKRLKDLELENSRLKK

>PA14_03190 | Max Lysine: 4

PMDPKKYPTSADDKDMAYEK

>PA14_03220 | Max Lysine: 4

PPYALPDHKILSTIKSKEYK

>PA14_03270 | Max Lysine: 4

QGQLKTLGEKLGDQKTELAK

>PA14_03360 | Max Lysine: 4

KSGPSYTEVVRDVASKLKVK

>PA14_03390 | Max Lysine: 4

SRLKVPQFKDFAQLWFDEKK

>PA14_03430 | Max Lysine: 4

AKAVTKVEEHIADAVSKGAK

>PA14_03510 | Max Lysine: 4

MKPAKLLVWELLKEGDPILK

>PA14_03610 | Max Lysine: 4

LKKRGVNTQGLVTAFEKLAK

>PA14_03650 | Max Lysine: 4

FGLRMKPKGERPGESAIKAK

>PA14_03730 | Max Lysine: 4

NSAKLSVKKWEFDLGWAWIK

>PA14_03900 | Max Lysine: 4

LREEKIIEKVKAETAPYLQK

>PA14_03940 | Max Lysine: 4

SGAYKKALEGNQQPKEVLVK

>PA14_04010 | Max Lysine: 4

DRLELRDKYVKRIAKNVSPK

>PA14_04090 | Max Lysine: 4

YRKNNPKFGEALNAALEKIK

>PA14_04110 | Max Lysine: 4

MSKTSLDKSKIKFLLLEGVH

>PA14_04140 | Max Lysine: 4

GMKVVTGKGDLLELNKDLIK

>PA14_04150 | Max Lysine: 4

TLREVQAKLKEKGLPWELAK

>PA14_04160 | Max Lysine: 4

ELSTKSLAEFPLGNIGKKNK

>PA14_04300 | Max Lysine: 4

MAAKFHLKKAKDGQFHFNLH

>PA14_04480 | Max Lysine: 4

VTTKKCHLKSIVHELLWFLK

>PA14_04580 | Max Lysine: 4

ADLKHFKAMTLGKPVIMGRK

>PA14_04630 | Max Lysine: 4

DKKGWKPAAPRARRVSTALK

>PA14_04640 | Max Lysine: 4

RFDVVIADPPAFIKRKKDLK

>PA14_04750 | Max Lysine: 4

DANVESKDQLMEKYRKITGK

>PA14_04810 | Max Lysine: 4

IMPSLHGIHYAAKRVKKWMK

>PA14_04870 | Max Lysine: 4

LKDVAKQAPVHRKLDIQEWK

>PA14_04890 | Max Lysine: 4

KGKTLEQVEAGLWKQLDDLK

>PA14_04910 | Max Lysine: 4

KPEIAKRVASALERVNLKEK

>PA14_05060 | Max Lysine: 4

EKGKPVEASVTGSAKDLTGK

>PA14_05110 | Max Lysine: 4

YQRELLQLKKKGDQFRLQLK

>PA14_05180 | Max Lysine: 4

LKGLVAKGLISRENAREKAK

>PA14_05190 | Max Lysine: 4

IPTLEELKLPEILKKLALTK

>PA14_05260 | Max Lysine: 4

SEGEFFKLYGLTEKRLKLAK

>PA14_05300 | Max Lysine: 4

ISKTLEITLATFKSDKAPEK

>PA14_05320 | Max Lysine: 4

FKSTPVIMLSSKDGLFDKAK

>PA14_05360 | Max Lysine: 4

IAKNATEAAAGKGEAFKLLK

>PA14_05530 | Max Lysine: 4

KTVPAKNVASAQKADAAPAK

>PA14_05560 | Max Lysine: 4

MLDMGFIHDVKKVLAKLPPK

>PA14_05620 | Max Lysine: 4

KNGTLKVPAINVNDSVTKSK

>PA14_05660 | Max Lysine: 4

SLYEWAKMKEAKIRQWVEEK

>PA14_05870 | Max Lysine: 4

LLALRLLVKNRALYKAPKGK

>PA14_05950 | Max Lysine: 4

LAHQALKRLSEGKIKGRSFK

>PA14_06040 | Max Lysine: 4

TKEADGSVFEHKLARIGKIK

>PA14_06290 | Max Lysine: 4

NDLAPKNKALLAKRDELQAK

>PA14_06600 | Max Lysine: 4

RKVLMTQGEALKGFTKIVHK

>PA14_06650 | Max Lysine: 4

EEVKRLPMNKPSGKYNVGNK

>PA14_06940 | Max Lysine: 4

ALHEKKAPSPCMSDAMSKNK

>PA14_06950 | Max Lysine: 4

LEPRKKAEEALRKSEERFAK

>PA14_07190 | Max Lysine: 4

VKAGDLVLFENVRFNKGEKK

>PA14_07230 | Max Lysine: 4

KKTKVDALAIAIGTSHGAYK

>PA14_07280 | Max Lysine: 4

AKHQVPYHEKKLGQLFCDNK

>PA14_07355 | Max Lysine: 4

TEFVPAVKKAFGALGKQHPK

>PA14_07430 | Max Lysine: 4

KDFKVSVVGVDKTATLNGLK

>PA14_07620 | Max Lysine: 4

SVKPLVEKGLKGAELGEALK

>PA14_07650 | Max Lysine: 4

KFAMKSFKDESFILQFLSPK

>PA14_07680 | Max Lysine: 4

GPVGGGKSSLAEKLKQLMEK

>PA14_07850 | Max Lysine: 4

ADFWDVKKFPGKRGLRWGAK

>PA14_07890 | Max Lysine: 4

SLMAKTARKLPFKEEPASYK

>PA14_08050 | Max Lysine: 4

IYATQDWVKEKVAADFKGRK

>PA14_08090 | Max Lysine: 4

GLLKEVDPGDWKAGEKAEFK

>PA14_08120 | Max Lysine: 4

AQRKRGQTPRRKRGKGGGVK

>PA14_08300 | Max Lysine: 4

MNKTITGHKGGSKKPRQPVE

>PA14_08370 | Max Lysine: 4

KLKHLDKLLAHCHRRRYTAK

>PA14_08380 | Max Lysine: 4

EPKVFTKIHVHFVVKGRGLK

>PA14_08390 | Max Lysine: 4

MKSKLKLHGFNNLTKTLSFN

>PA14_08460 | Max Lysine: 4

GYDLKRLTDKADMNYGEKLK

>PA14_08490 | Max Lysine: 4

DDRKLDGRLKVRISGKLARK

>PA14_08680 | Max Lysine: 4

KPGTIKPHTKFECEVYVLSK

>PA14_08720 | Max Lysine: 4

MAKKIQAYIKLQVKAGQANP

>PA14_08740 | Max Lysine: 4

MAIKLEDKKAIVAEVNEAAK

>PA14_08750 | Max Lysine: 4

GDKKVNVIKVVRELTGLGLK

>PA14_08810 | Max Lysine: 4

KYGSQILAKFMNHVMESGKK

>PA14_08820 | Max Lysine: 4

VAVEPKTKADQEKMGIALGK

>PA14_08830 | Max Lysine: 4

KPGTIKPHTKFECEVYVLSK

>PA14_08900 | Max Lysine: 4

QIRGKKVGEALNLLAFSSKK

>PA14_08910 | Max Lysine: 4

GIVIGKKGEDVEKLRQDLTK

>PA14_08940 | Max Lysine: 4

LIERRVKHPIYGKYVKRSTK

>PA14_08970 | Max Lysine: 4

MARLKEIYRKEIAPKLKEEL

>PA14_09010 | Max Lysine: 4

MSVKKETRLRRARKARLKMR

>PA14_09020 | Max Lysine: 4

TFKGLKNMQAPEAVAAKRGK

>PA14_09030 | Max Lysine: 4

KVTLVKSLNGRLANHKACVK

>PA14_09070 | Max Lysine: 4

MKVRASVKKLCRNCKIIRRD

>PA14_09080 | Max Lysine: 4

GQRTKTNARTRKGPRKPIRK

>PA14_09115 | Max Lysine: 4

KGLAIKLHGRDEVTLTLAKK

>PA14_09820 | Max Lysine: 4

YLGGMPMLMITGQKPIKKSK

>PA14_10230 | Max Lysine: 4

CIGNKHTAKLAIDAIRKAGK

>PA14_10260 | Max Lysine: 4

KKTGVCQGKGGSMHIADLEK

>PA14_10480 | Max Lysine: 4

FGEEKLAQLLELLNELKKIK

>PA14_10540 | Max Lysine: 4

RIKLDQGPASAAKLLRKTAK

>PA14_10550 | Max Lysine: 4

YNRYGRRDNKYKARIKILVK

>PA14_11060 | Max Lysine: 4

VEAAPQKATIDKNTATLKYK

>PA14_11070 | Max Lysine: 4

DSAAGKLQWKFLESGGAGKK

>PA14_11110 | Max Lysine: 4

PPPPIPKKCKVEVGREINVK

>PA14_11120 | Max Lysine: 4

RSVQTISTQKVSAMKKLGIK

>PA14_11450 | Max Lysine: 4

QDSGNPAAKPPKGKTAARRK

>PA14_11550 | Max Lysine: 4

RNMRDMKGPQFLHVVTKKGK

>PA14_12030 | Max Lysine: 4

RQVKSLADNVLTKAKENGVK

>PA14_12130 | Max Lysine: 4

STVVEKSGEAKPGKVEVGVK

>PA14_12200 | Max Lysine: 4

VLLLSLPKLDGSTQKTKWAK

>PA14_12230 | Max Lysine: 4

LKSDGQPVEIGGTEKMSKSK

>PA14_12350 | Max Lysine: 4

IREKAQEKVFSKLGMWRELK

>PA14_12560 | Max Lysine: 4

LILFMAFIVWDLAKKSKAGK

>PA14_12620 | Max Lysine: 4

PEVLKDAVGADGKVDNRKLK

>PA14_12780 | Max Lysine: 4

SNKAIGEKLFLSNKTVSTYK

>PA14_12900 | Max Lysine: 4

QTGKAIEIAAKRVAKFVPAK

>PA14_13040 | Max Lysine: 4

GVAFEFRFKAKPAKQHIWDK

>PA14_13110 | Max Lysine: 4

SQIAVVTDIPKTSVGKLDKK

>PA14_13130 | Max Lysine: 4

FKRGETFSKVFKGIHDLELK

>PA14_13360 | Max Lysine: 4

WARAKVKSDLLQKAEVHSDK

>PA14_13430 | Max Lysine: 4

PGIRYEKIDSEQKNLLKNSK

>PA14_13600 | Max Lysine: 4

VYTTDGRLKDFKLRVLKDDK

>PA14_13730 | Max Lysine: 4

NKMIARKLDITEGTVKVHVK

>PA14_13780 | Max Lysine: 4

WYIYSANRLKYPKVRKPLLK

>PA14_13870 | Max Lysine: 4

IPTQIEKRDRELYELVKKSK

>PA14_13950 | Max Lysine: 4

GKMWLTTKATLEVVSLATKK

>PA14_14140 | Max Lysine: 4

AGLEYQVKVVSDPRKAPKGK

>PA14_14150 | Max Lysine: 4

SKGDALRRERAIKALSKRAK

>PA14_14310 | Max Lysine: 4

KALSNRTRLEILKGLKDPAK

>PA14_14380 | Max Lysine: 4

VLVTVALVIPMMKKRLLGKK

>PA14_14390 | Max Lysine: 4

DMLALDKQIELIKKVVPGAK

>PA14_14440 | Max Lysine: 4

LLQKRTSGMMQPKLAEKIAK

>PA14_14610 | Max Lysine: 4

WRPQAKRAKEHKNLLGNLQK

>PA14_14630 | Max Lysine: 4

AAKALTDAGIAVKANSLSKK

>PA14_14730 | Max Lysine: 4

AIKGVAHFYSGKGKHIITSK

>PA14_14740 | Max Lysine: 4

CSVLAEDAIKAAVRDYKQKK

>PA14_14850 | Max Lysine: 4

LGLVSLQMKKPAQAKEYFEK

>PA14_14860 | Max Lysine: 4

GKVLVSALKRKGDSLELAGK

>PA14_14890 | Max Lysine: 4

RLLVNAGAGSFKSQFKKADK

>PA14_14910 | Max Lysine: 4

KKELPPAELTDFKEEVVLSK

>PA14_14990 | Max Lysine: 4

LIGKWLKRTGKRDRMVIASK

>PA14_15030 | Max Lysine: 4

PMDAAKKMRFFKTLVQVGLK

>PA14_15070 | Max Lysine: 4

SPKRGPNGSVNAFDKIKPEK

>PA14_15310 | Max Lysine: 4

IIHKNMGIEQQAAEVRKVKK

>PA14_15470 | Max Lysine: 4

CSACPITVKKAISKVEGVSK

>PA14_15520 | Max Lysine: 4

QLASQSQKKANDALFKGLDK

>PA14_15530 | Max Lysine: 4

MPEVNDANCQIEKIKKIEDK

>PA14_15570 | Max Lysine: 4

KAGKKTVYRLAQQGEIPGFK

>PA14_15580 | Max Lysine: 4

VTQTLAALKASPATAKAKAK

>PA14_15600 | Max Lysine: 4

IKDGSITGYIYDPKAAKLVK

>PA14_15610 | Max Lysine: 4

PFPLKLLGALAQLCKSVKDK

>PA14_15670 | Max Lysine: 4

KAWEGGEEHKHNAPPLAKLK

>PA14_16020 | Max Lysine: 4

ETLIEQGLLSERQLKKALKK

>PA14_16050 | Max Lysine: 4

VKDGKPVNLTEKAESQAIAK

>PA14_16130 | Max Lysine: 4

RNKKSLTLNLKHAEGQAILK

>PA14_16150 | Max Lysine: 4

IKASGNSVDGVLARLKEKGK

>PA14_16180 | Max Lysine: 4

EREVELQKIEKEKALEEQRK

>PA14_16250 | Max Lysine: 4

QVLAQAKSLKAQGRKTENDK

>PA14_16370 | Max Lysine: 4

AAGAIGLLLGNKKARKFGGK

>PA14_16410 | Max Lysine: 4

LSMRESPAFLKMKAEGKVSK

>PA14_16510 | Max Lysine: 4

NKDTAMKMLRAKLYELEMQK

>PA14_16530 | Max Lysine: 4

QKQYADKTKEELEAAAIPVK

>PA14_16580 | Max Lysine: 4

KWDDGIRQDFLKGRGKHAPK

>PA14_16630 | Max Lysine: 4

TKVAALETKDAGDWLAKADK

>PA14_16640 | Max Lysine: 4

WDARVAERKTQAAKAQKAVK

>PA14_16770 | Max Lysine: 4

PKPGYSLEGKSSRYDIYPKK

>PA14_16830 | Max Lysine: 4

NPDVRIELPGKLGKAAKLMK

>PA14_16950 | Max Lysine: 4

LEARLKGKLLEVFSVDKFPK

>PA14_16970 | Max Lysine: 4

YGIKACDTMKKARTWLDEHK

>PA14_17040 | Max Lysine: 4

KHLRKLNRPEMAEKYPLASK

>PA14_17060 | Max Lysine: 4

KMGKFIFGARNKIHIINLEK

>PA14_17070 | Max Lysine: 4

DIEKAIDDMRAAGAIKAAKK

>PA14_17100 | Max Lysine: 4

DVQKLTDKFIGEIEKALEAK

>PA14_17120 | Max Lysine: 4

YFSGKAFGKRKLAPRVSPGK

>PA14_17150 | Max Lysine: 4

SIEIEGNKAISKEDLLKGLK

>PA14_17250 | Max Lysine: 4

LSNLAIKLAKDYNAQRKAGK

>PA14_17310 | Max Lysine: 4

RGPGLEEGMKIFEEIKKTFK

>PA14_17320 | Max Lysine: 4

NTKILKEGIEKGIGNSILIK

>PA14_17500 | Max Lysine: 4

KKAAKLLDITLTARGQSGGK

>PA14_17530 | Max Lysine: 4

VKEGDEVVGSETRVKVVKNK

>PA14_17600 | Max Lysine: 4

AHAASLKDFELSKMLEKVAK

>PA14_17690 | Max Lysine: 4

EIKLSQGAKPGHGGILPKHK

>PA14_17720 | Max Lysine: 4

SKEIARKLAISAETVKVHRK

>PA14_17780 | Max Lysine: 4

LKLEESEDMKRVLAQKRTVK

>PA14_17790 | Max Lysine: 4

KKLEEQLGTQLLLRNNKSVK

>PA14_17990 | Max Lysine: 4

DLLKKARAEHKVLSYSHDPK

>PA14_18040 | Max Lysine: 4

RLQKELAWKGETRGQGKHDK

>PA14_18150 | Max Lysine: 4

VVKRLPKTRSGKILRAVLRK

>PA14_18380 | Max Lysine: 4

KDAMMIAHKDRVQDVKHVVK

>PA14_18410 | Max Lysine: 4

SQPGGKPQLVAEPPFKNKQK

>PA14_18500 | Max Lysine: 4

VRDSKVTGWSESKKEPAWFK

>PA14_18580 | Max Lysine: 4

KKAVLDSDVSFICVGTPSKK

>PA14_18630 | Max Lysine: 4

RAEVKRDLQYGAVAGKQKAK

>PA14_18670 | Max Lysine: 4

MKGDKKVIQHLNKILGNELI

>PA14_18690 | Max Lysine: 4

PANWKKGDKGMTASPEGVAK

>PA14_18880 | Max Lysine: 4

NRTGIAPGKNVLEVEKKLLK

>PA14_18960 | Max Lysine: 4

GYDRQKWKKSALLDSLHIGK

>PA14_18970 | Max Lysine: 4

KNDAIVGKAIHQLRVNLWKK

>PA14_19120 | Max Lysine: 4

ESTVNFHHKNIQKKFDAPNK

>PA14_19360 | Max Lysine: 4

KALYRKLGFRELATFTIKRK

>PA14_19370 | Max Lysine: 4

PARFKLGDGGKQVLKGAARK

>PA14_19430 | Max Lysine: 4

EMGIGPVFSVPKLLKAKGLK

>PA14_19470 | Max Lysine: 4

DWKLVQAGQRVQIIKKDAEK

>PA14_19490 | Max Lysine: 4

SLKDEEEIKRRFPKGYRAVK

>PA14_19500 | Max Lysine: 4

KDSPIRSLAELKGRKVAATK

>PA14_19540 | Max Lysine: 4

KDSPIRSVAELKGRKVALNK

>PA14_19610 | Max Lysine: 4

EWKAKYQKEASPEQQAAFAK

>PA14_20200 | Max Lysine: 4

QIKTRKIWDRKDPFFAETVK

>PA14_20530 | Max Lysine: 4

IDARFAQKKILFQLDRLEKK

>PA14_20670 | Max Lysine: 4

KHLLKEVALKHGLIVVCMAK

>PA14_21030 | Max Lysine: 4

YALKVRKVFVTGGVDEKMAK

>PA14_21110 | Max Lysine: 4

VASQIKLPKPKPPAVAAMPK

>PA14_21175 | Max Lysine: 4

LRKFKPLGAYLEKQLGMPVK

>PA14_21310 | Max Lysine: 4

PVKLGDDLKVELEVLEKLPK

>PA14_21340 | Max Lysine: 4

DLLPALKGWLVNSVVKSVKK

>PA14_21370 | Max Lysine: 4

DILPPLKRFIVNFVVKHIKK

>PA14_21480 | Max Lysine: 4

KVIVEWEKDPNPRAVIKRDK

>PA14_21540 | Max Lysine: 4

ESGIGKRDKLFVQEGRKVFK

>PA14_21620 | Max Lysine: 4

VIKTKGGLEVATTDKEFSFK

>PA14_21640 | Max Lysine: 4

MGVVKGCKAFLPLLERSKGK

>PA14_21670 | Max Lysine: 4

AEAEKMGKEVATKVASITQK

>PA14_21730 | Max Lysine: 4

RDRPVLGLNGKPVQEKKRSK

>PA14_21960 | Max Lysine: 4

DSWANWKDTWKDLESKYGLK

>PA14_22020 | Max Lysine: 4

RDKDALTKEGVEKVMAELRK

>PA14_22040 | Max Lysine: 4

PAKAEEKPADPVSRPTKVVK

>PA14_22050 | Max Lysine: 4

HRRFKTRPPGEAKLYPKRRK

>PA14_22110 | Max Lysine: 4

GSPRSLTRNQRATAKDKKAK

>PA14_22120 | Max Lysine: 4

RVQTKLSQFAKTANQKIQFK

>PA14_22130 | Max Lysine: 4

SKTPVIETKYDRERMQVQKK

>PA14_22350 | Max Lysine: 4

YASVFKGGKANEKDELRVSK

>PA14_22480 | Max Lysine: 4

AQFGRDIIKLRIKLTCKIAK

>PA14_22540 | Max Lysine: 4

FKKFADATSRKWFVRQWQDK

>PA14_22620 | Max Lysine: 4

VWEMIFSGKKSVRLETQRKK

>PA14_22820 | Max Lysine: 4

KDRADAFRKWLGAGKLGDVK

>PA14_22870 | Max Lysine: 4

IVASFGKDVAFNIKPKQLLK

>PA14_23110 | Max Lysine: 4

ASAPGDKPAARKPQVKRRPK

>PA14_23190 | Max Lysine: 4

LAKKFDREASLIGLAKPELK

>PA14_23280 | Max Lysine: 4

KEPLIKDVLEKIGQEAVALK

>PA14_23340 | Max Lysine: 4

KTGESVRLDGKFVPHFKPGK

>PA14_23430 | Max Lysine: 4

EVKVSMKADGFVADMGAGKK

>PA14_23440 | Max Lysine: 4

MVTKPRKEFVERFAREKIMK

>PA14_23510 | Max Lysine: 4

KDIKDILEGAVVPGARGKRK

>PA14_23560 | Max Lysine: 4

QLCYMPLLRNPDKSKLSKRK

>PA14_23620 | Max Lysine: 4

SLKLFPADGTASVKMLKSLK

>PA14_23750 | Max Lysine: 4

EHVLATQCLVAKKMKNMLVK

>PA14_23920 | Max Lysine: 4

ARKKSVRQKLNAIELEFRGK

>PA14_24040 | Max Lysine: 4

ELDGQPLVLAGSKGEKEQKK

>PA14_24070 | Max Lysine: 4

GRFNLNGLVRKRKVKPDSVK

>PA14_24170 | Max Lysine: 4

KAEVHPAARQVFLLQRKKSK

>PA14_24245 | Max Lysine: 4

KLAIEIGKWPDGRKLTQEQK

>PA14_24300 | Max Lysine: 4

RYDWKLLGKKEIYIPYNNYK

>PA14_24330 | Max Lysine: 4

DKYQLQNPNVKVHIVGFAKK

>PA14_24370 | Max Lysine: 4

LKLPAAKALMVVEPGKSQGK

>PA14_24440 | Max Lysine: 4

QSLYQLLWKLYKRNPAEWKK

>PA14_24570 | Max Lysine: 4

GTQRLVDMQKLQKRIEEAKK

>PA14_24675 | Max Lysine: 4

KRRERQAGKKQYERQSSEGK

>PA14_24730 | Max Lysine: 4

YLKYPWTSRHAEALGYKKHK

>PA14_24790 | Max Lysine: 4

ITAKLRASKSTLKIGTLTPK

>PA14_25020 | Max Lysine: 4

PAPAAAAPSEAPAKKKLSYK

>PA14_25090 | Max Lysine: 4

KFGARSHQLAWKATQEGKFK

>PA14_25110 | Max Lysine: 4

MGKSLVIVESPAKAKTINKY

>PA14_25230 | Max Lysine: 4

KNLMRLTLLKLQAEKLGILK

>PA14_25250 | Max Lysine: 4

SELKLGAASVDLGKMVAKFK

>PA14_25330 | Max Lysine: 4

WKKDQVEAPTYKMAPQVSSK

>PA14_25390 | Max Lysine: 4

RVEGLDNGVILHLKSGKKIK

>PA14_25560 | Max Lysine: 4

LKEISREYFKKSPEGRINIK

>PA14_25580 | Max Lysine: 4

KIVDKRYHALVRGHWPAGKK

>PA14_25800 | Max Lysine: 4

MIADAVGIGKGTIYKHFKSK

>PA14_25900 | Max Lysine: 4

VKQQIDYVEAKGPVVNGPKK

>PA14_26020 | Max Lysine: 4

LVGSTHYVQNLAPEEKKKIK

>PA14_26310 | Max Lysine: 4

IAAVGKPDIAINTVGKVLKK

>PA14_26470 | Max Lysine: 4

LKHLRKAPVEKLSLCGGFGK

>PA14_26700 | Max Lysine: 4

DEIMLGIICKLMGILPGKKK

>PA14_26720 | Max Lysine: 4

RELEQKVLDKAAEARPKFEK

>PA14_27370 | Max Lysine: 4

IRGIAERHLKQPKHVKIAAK

>PA14_27520 | Max Lysine: 4

LKKRALGLLGSQGIKWNFTK

>PA14_27590 | Max Lysine: 4

KKADAGLKARIEELRALVAK

>PA14_28180 | Max Lysine: 4

IEAIIRPKMLKTFPQLKDVK

>PA14_28390 | Max Lysine: 4

QAQSTAYNYCMQNSDKKKRK

>PA14_28420 | Max Lysine: 4

KNELKLPVHAIWLKNQPLRK

>PA14_28650 | Max Lysine: 4

KDYDVIKKMTPRAEVIELFK

>PA14_28750 | Max Lysine: 4

RDIKLKLIQPGKPTQNAFIK

>PA14_28760 | Max Lysine: 4

TVPDAKRLKNLDLENSRLKK

>PA14_28820 | Max Lysine: 4

KAHLRLQLNQPPTFQSLKKK

>PA14_29110 | Max Lysine: 4

KVLKALGAELVLTEPAKGMK

>PA14_29160 | Max Lysine: 4

EKAQIQSLGGLDKLIEEFKK

>PA14_29230 | Max Lysine: 4

FTQKPKEELADFKNWTGSVK

>PA14_29350 | Max Lysine: 4

TDDLSLQATVTWYGKQKPKK

>PA14_29400 | Max Lysine: 4

ANRAAKLAARKAAHADTIKK

>PA14_29510 | Max Lysine: 4

FSQADKAKAQTADTQVKMLK

>PA14_29940 | Max Lysine: 4

NKALIEAAANIAKALKQREK

>PA14_30030 | Max Lysine: 4

MNVPDDKLPLIKTMKIDYLK

>PA14_30190 | Max Lysine: 4

PVMIKVVDAAVEKAYKGERK

>PA14_30230 | Max Lysine: 4

TKYRGDFEKRFKALLNELRK

>PA14_30280 | Max Lysine: 4

IHRRDKLRSEKILQDKLFDK

>PA14_30290 | Max Lysine: 4

KRPPPKIDPPPPPKAPEPSK

>PA14_30310 | Max Lysine: 4

AITYKEGGNVVDFVLKPKTK

>PA14_30320 | Max Lysine: 4

ALRKLVGRALNEDKGLGKRK

>PA14_30370 | Max Lysine: 4

NHLFKGAPAKLGAKLAGLPK

>PA14_30550 | Max Lysine: 4

EHSPLKSLAELKGKRIAVTK

>PA14_30710 | Max Lysine: 4

AQAAQAKPAQTKSSAGRAKK

>PA14_30800 | Max Lysine: 4

AKDELKKIVPADAFAATETK

>PA14_30980 | Max Lysine: 4

IDLKDDSTRSLKARCDARKK

>PA14_30990 | Max Lysine: 4

KGAEVKMHLKQGEGVVFQWK

>PA14_31070 | Max Lysine: 4

SAPKNRGKGQGQSFVSKVLK

>PA14_31270 | Max Lysine: 4

KELETTRRLLESRTPKQPKK

>PA14_31280 | Max Lysine: 4

KKVHEVTRNDITALMKRMEK

>PA14_31390 | Max Lysine: 4

GKGNPHGNQMDGGKGNKGNK

>PA14_31680 | Max Lysine: 4

FQAQIDSEVKDGKPPKLTLK

>PA14_31750 | Max Lysine: 4

IIFPKGTRNLNNKVKLLPFK

>PA14_31760 | Max Lysine: 4

YICGKLFGKRKIAPNLSPSK

>PA14_32100 | Max Lysine: 4

YDLDVRGESPLIKAKKVILK

>PA14_32160 | Max Lysine: 4

KVKAPGEYPEGFDKATRGLK

>PA14_32250 | Max Lysine: 4

QKELREKRLQKQQLEAQAQK

>PA14_32400 | Max Lysine: 4

KTYAATLIKDEAVGTDLGKK

>PA14_32410 | Max Lysine: 4

YTDELPANAKRKTVRRSKPK

>PA14_32610 | Max Lysine: 4

KDPQKALHDHEQAGKASTLK

>PA14_32905 | Max Lysine: 4

AELAEKRPGALKAFWKQVAK

>PA14_33030 | Max Lysine: 4

LDKVIRTMRQTGADMKSKYK

>PA14_33360 | Max Lysine: 4

APKTYNHLKEKEIPSFSRQK

>PA14_33480 | Max Lysine: 4

TTPKQLIAGMVKGQSGKAGK

>PA14_33510 | Max Lysine: 4

PEYKEIPQGWRAAGKSGLKK

>PA14_33560 | Max Lysine: 4

EPSPSQLKKTIDQLKALDVK

>PA14_33600 | Max Lysine: 4

ATAALVKRGKRPARQPKPAK

>PA14_33710 | Max Lysine: 4

TAPAFADEAKKPGTVFKDCK

>PA14_33730 | Max Lysine: 4

EEPKAGLKELVDAIDYTVKK

>PA14_33830 | Max Lysine: 4

SPVKQMLKEAGLKMSLARLK

>PA14_33900 | Max Lysine: 4

VPARDKLLLKKPEQFRYIGK

>PA14_33910 | Max Lysine: 4

KWKARDVLLRYKPIGFARQK

>PA14_33960 | Max Lysine: 4

KTNSSKGGGGFNELRFEDKK

>PA14_33990 | Max Lysine: 4

GIVVAKLEKLRERYKAATGK

>PA14_34030 | Max Lysine: 4

SLNFTKIKWELTAQKDDGTK

>PA14_34130 | Max Lysine: 4

LKLADDLLGLKRAIDDLKTK

>PA14_34140 | Max Lysine: 4

SGDSKSAKAALEAAQLQQKK

>PA14_34600 | Max Lysine: 4

EAVVKLLMWEIGKNLKDSEK

>PA14_34880 | Max Lysine: 4

QKEGLIFKIHGKGTFVARPK

>PA14_34900 | Max Lysine: 4

KAKEHDRELRVLLVDKANVK

>PA14_35030 | Max Lysine: 4

KGKDPRITIMSAGLQSDGKK

>PA14_35690 | Max Lysine: 4

LANRPPPEGLWKRTKFYVKK

>PA14_35740 | Max Lysine: 4

GRRQSDFNTLKQAPKKPSRK

>PA14_35790 | Max Lysine: 4

CKKPSNKEQWAILSQRLGVK

>PA14_35800 | Max Lysine: 4

DIQAVDKEGKSRRRKGTSIK

>PA14_35810 | Max Lysine: 4

AFFFKQWGGWGSDGVKRSKK

>PA14_36010 | Max Lysine: 4

MNEHKEKIRNQLETINYKRK

>PA14_36020 | Max Lysine: 4

SDGIQAKKTEVLYKGIAVGK

>PA14_36200 | Max Lysine: 4

AKSKQIEGLDVDYAKALADK

>PA14_36620 | Max Lysine: 4

EAEAAAAAPKKTRGRAKAAK

>PA14_36820 | Max Lysine: 4

QGGKGNFAEDPKRASEAGKK

>PA14_36840 | Max Lysine: 4

VKIFAGKAAASYHQAKLIIK

>PA14_36980 | Max Lysine: 4

EAGKMALEKSTAADVKVFAK

>PA14_37680 | Max Lysine: 4

LHEKERQLQEKEKQLRQWHK

>PA14_37710 | Max Lysine: 4

VAVEPKTKADQEKMGIALSK

>PA14_38300 | Max Lysine: 4

QRLKEEGVIRKQVTLLDRKK

>PA14_38350 | Max Lysine: 4

DALMKQAQDGCVLAYKFKGK

>PA14_38700 | Max Lysine: 4

AAQLKQRLAKGEDFATLAKK

>PA14_39130 | Max Lysine: 4

KKLHRQAVTVENISKGYDGK

>PA14_39270 | Max Lysine: 4

VVSLRVKAPTGKDPYGIKLK

>PA14_39480 | Max Lysine: 4

KIDAINARIKNRKQHEEDIK

>PA14_39780 | Max Lysine: 4

SPKSQKSGGVSASYLRKDAK

>PA14_40200 | Max Lysine: 4

AAKQGRDALKVEWDESKAEK

>PA14_40230 | Max Lysine: 4

AKIKPRDVAFLVPGMPAKVK

>PA14_40280 | Max Lysine: 4

LLGTPIEYGLKKLPRKVNEK

>PA14_40340 | Max Lysine: 4

ALLKAALAKKNQAQLYPQRK

>PA14_40770 | Max Lysine: 4

YNRYGRRDNKYKARIKILVK

>PA14_40880 | Max Lysine: 4

KALTPDAPLWEPVKAWLKDK

>PA14_41090 | Max Lysine: 4

DLKRWNGLDKHALKVGQTLK

>PA14_41230 | Max Lysine: 4

KKVLAVAVYNHYKRLNQRDK

>PA14_41250 | Max Lysine: 4

QKLNPAGSPSVEPKSFEKGK

>PA14_41260 | Max Lysine: 4

CISKLRNKLKDNPREPVRIK

>PA14_41390 | Max Lysine: 4

LKLDEEKAPETAANFKEYVK

>PA14_41400 | Max Lysine: 4

TRHKLARKLRKESRAQTRMK

>PA14_41420 | Max Lysine: 4

TEKVTDAKGIPVRKQQQFRK

>PA14_41440 | Max Lysine: 4

KQHFPDNPLKKAILTPDDWK

>PA14_41470 | Max Lysine: 4

GEWFKAKPEVPEKLTLTVFK

>PA14_41490 | Max Lysine: 4

AQLAERKRIELAKGLLMKMK

>PA14_41500 | Max Lysine: 4

MSVEKVPSKNKVIIGQIHSK

>PA14_41530 | Max Lysine: 4

VKAIQEHGLFSVDEVKKHTK

>PA14_41670 | Max Lysine: 4

GSKAIKMIYGDEAKAGRSVK

>PA14_41690 | Max Lysine: 4

KLDTGAKTASLSARDIKRFK

>PA14_41710 | Max Lysine: 4

KQTLYYRLVLTKRYTGEKTK

>PA14_41730 | Max Lysine: 4

VLKYNKRHLYPVVDDKIITK

>PA14_41750 | Max Lysine: 4

FSTGVKLRPFKKWQFNFDLK

>PA14_41760 | Max Lysine: 4

AKALEEVFQAAEKSYTLLKK

>PA14_41800 | Max Lysine: 4

HNLFAKLGVKSRTQAVLKGK

>PA14_41860 | Max Lysine: 4

MRITMVKKVLATGEDCRKCK

>PA14_41870 | Max Lysine: 4

ILRKVESIKQIAQEFSNEKK

>PA14_41920 | Max Lysine: 4

ATTEKSIRSMHAKLKDVLPK

>PA14_42060 | Max Lysine: 4

GWRKVSSGELYKWQFSKGTK

>PA14_42080 | Max Lysine: 4

SAKKTFFAGGDLNELIKVTK

>PA14_42090 | Max Lysine: 4

DAVRTPRGKGKKDGALHSVK

>PA14_42270 | Max Lysine: 4

LKRKGYPRENFSTLKDVFPK

>PA14_42440 | Max Lysine: 4

GSLGALKNGKAISQEKTLQK

>PA14_42470 | Max Lysine: 4

YKDVLQTQDGKRKALLDELK

>PA14_42500 | Max Lysine: 4

QGAGAPSAKARGKAGGGKPK

>PA14_42670 | Max Lysine: 4

YSDWALGQFFEKAKKSPYYK

>PA14_43030 | Max Lysine: 4

QKPKFFGISAEGKAAELNYK

>PA14_43050 | Max Lysine: 4

PVLRKDGLKAAVQVLKQGMK

>PA14_43110 | Max Lysine: 4

RAEFEEKKIGGYKALDVMEK

>PA14_43180 | Max Lysine: 4

ASKHAVIGLTKSAAIEYAKK

>PA14_43540 | Max Lysine: 4

DKAPQKLGEALKAQLALRHK

>PA14_43610 | Max Lysine: 4

KFQMGVYSKMPAQKLDRLLK

>PA14_43680 | Max Lysine: 4

KFFGQVLPTAKKVTYNIHIK

>PA14_43970 | Max Lysine: 4

AAQLGLKTACIEKYIGKEGK

>PA14_44010 | Max Lysine: 4

KKVDRVVLCSGKVYYDLLEK

>PA14_44060 | Max Lysine: 4

MKKAVNSKRPVNLDLRTIKL

>PA14_44080 | Max Lysine: 4

KLAKDEDGVKVYLSSVQGSK

>PA14_44240 | Max Lysine: 4

KRLVKGVAHKHGMLACFMAK

>PA14_44280 | Max Lysine: 4

KEVSTFCRKFEKPLRAALVK

>PA14_44350 | Max Lysine: 4

KMPAYPWLVENKLDGKDIEK

>PA14_44380 | Max Lysine: 4

LVENKLDGKDTATKMEVLRK

>PA14_44390 | Max Lysine: 4

KKSFDEATMLPFADDPEAKK

>PA14_44400 | Max Lysine: 4

FADKEKGWTGVHQWEKEMAK

>PA14_44510 | Max Lysine: 4

PGFHPSDHDTAKLEKYWKDK

>PA14_44560 | Max Lysine: 4

KCWEKYRLQEKTLLNTEIGK

>PA14_44590 | Max Lysine: 4

SLLLVEKGTAGFSVGRKLKK

>PA14_44620 | Max Lysine: 4

KGGMAGLMKQAQQMQEKMQK

>PA14_44660 | Max Lysine: 4

VAKDKLEGLGAKVAGSVSAK

>PA14_44710 | Max Lysine: 4

KILVPRARPSQAFKAYKVSK

>PA14_44780 | Max Lysine: 4

DLHAVFSHLLGGKKKPRRAK

>PA14_44880 | Max Lysine: 4

HLEKYKLAPPKMPENLHWFK

>PA14_44900 | Max Lysine: 4

PPYALPDHKILSTIKSKEYK

>PA14_44910 | Max Lysine: 4

AGVKASFRHGSKGKGQANTK

>PA14_45000 | Max Lysine: 4

KAAGKLKSRAAWLESCQQRK

>PA14_45100 | Max Lysine: 4

VTLLEPKAQEIKREDDGKLK

>PA14_45280 | Max Lysine: 4

FSGGKKMTAQIKALADEALK

>PA14_45330 | Max Lysine: 4

PEVTKALKDSGQLKHYENGK

>PA14_45350 | Max Lysine: 4

LKRESRTAWAKAEVKALVEK

>PA14_45590 | Max Lysine: 4

AVAKNIAAKSPAAKPVAPAK

>PA14_45620 | Max Lysine: 4

VNGYVVKPFTAQVLKEKIEK

>PA14_45800 | Max Lysine: 4

KMKPLRGTALFILDAKLVFK

>PA14_46070 | Max Lysine: 4

GGDHTITLPILRAIKKKHGK

>PA14_46220 | Max Lysine: 4

NYLKAEVFQPLDKSKLPNWK

>PA14_46340 | Max Lysine: 4

TECSAKAGQWLQKARAKAAK

>PA14_46540 | Max Lysine: 4

LKQKGAQVKRMHFRRQQHNK

>PA14_46570 | Max Lysine: 4

KTTVSDLAKAIGFSKAYIYK

>PA14_46620 | Max Lysine: 4

RLKTQFHKKFARGVEYRLGK

>PA14_46650 | Max Lysine: 4

ALKDLFPLPPSRPKSRKASK

>PA14_46740 | Max Lysine: 4

RGDARLDNRKIKAAFGGKAK

>PA14_46850 | Max Lysine: 4

KFHKSNIFQKVGCSCIGAFK

>PA14_46950 | Max Lysine: 4

NLTIAQIKVLGRSKEEATKK

>PA14_46990 | Max Lysine: 4

TQASQDLGMAKTTLFDKVKK

>PA14_47090 | Max Lysine: 4

RKGFDEAFLGPKAALPKPGK

>PA14_47190 | Max Lysine: 4

KVQDVDAFHDMKKAGTAYRK

>PA14_47540 | Max Lysine: 4

GLSKLKRTEVSGGLAIVKAK

>PA14_47790 | Max Lysine: 4

EKDQRHRERMERKKAVVDEK

>PA14_47920 | Max Lysine: 4

VVSSTVAASWIKSGKYKGLK

>PA14_48150 | Max Lysine: 4

SMTKDEKEKTHVDAIIERYK

>PA14_48760 | Max Lysine: 4

NINAWDNEDFVKAVKATGKK

>PA14_48870 | Max Lysine: 4

YHIVEKDTYSVVKEKIPEGK

>PA14_48910 | Max Lysine: 4

TATGKTQDTKAGKSLFRSPK

>PA14_49510 | Max Lysine: 4

MEKKLIVKLIDSIGKSHEEI

>PA14_49740 | Max Lysine: 4

ATNYIGDFRVQCSYKEKKGK

>PA14_49840 | Max Lysine: 4

RPAYEPKPDKAAANHYLNKK

>PA14_49920 | Max Lysine: 4

NKSKNVWWFDIPLSQLKAGK

>PA14_49940 | Max Lysine: 4

DKAFFEGAATYVKQLRQQKK

>PA14_49990 | Max Lysine: 4

MAIMKKTVAPKAAKPAPAVE

>PA14_50130 | Max Lysine: 4

LALKGSDEAIREKVFKNMSK

>PA14_50270 | Max Lysine: 4

KPITLTVSRDDAGVKENVKK

>PA14_50460 | Max Lysine: 4

KGSSNLGKDEFLKLLVAQLK

>PA14_50480 | Max Lysine: 4

KARDLDFAAVLAEQKDKAAK

>PA14_50520 | Max Lysine: 4

AGKFIAERYKDKTIAVLHDK

>PA14_50680 | Max Lysine: 4

MLAKAAVLQQLPLEKKTRGK

>PA14_50750 | Max Lysine: 4

PQKRDYLLQRLAEAKASKVK

>PA14_50810 | Max Lysine: 4

GRDVAAKAAEQKAEIAAAKK

>PA14_50830 | Max Lysine: 4

LRLSMDSPAKRAFVKQLKAK

>PA14_50840 | Max Lysine: 4

LKKGFSKLTDLFTRLTDILK

>PA14_50900 | Max Lysine: 4

ALKLGRNDPCPCGAGGKLKK

>PA14_51050 | Max Lysine: 4

KTRNDVANKGRLSLPGKPTK

>PA14_51060 | Max Lysine: 4

MDKKKLLRLITEKLAADLEV

>PA14_51240 | Max Lysine: 4

EFSPDGCRLWDKETRKKMDK

>PA14_51260 | Max Lysine: 4

KKPDEDKPGFFSRLFGGGEK

>PA14_51320 | Max Lysine: 4

LKQVRADLMLKMSKPQESEK

>PA14_51550 | Max Lysine: 4

KTDSTKFSVVLKSGGRSIDK

>PA14_51650 | Max Lysine: 4

KSKVMNSDQVEAMYRKLTEK

>PA14_51790 | Max Lysine: 4

LVKIPGVGKKTAERLLVELK

>PA14_52130 | Max Lysine: 4

IDKLRGEQARLKTLKASELK

>PA14_52160 | Max Lysine: 4

KPDAAKLAEAAVKQRWEELK

>PA14_52465 | Max Lysine: 4

GRSVTPMAETKTKKASIGRK

>PA14_52530 | Max Lysine: 4

VILQTGGRKLRFGKYTLRKK

>PA14_52720 | Max Lysine: 4

PEVLDGVKAKHERFKSRLQK

>PA14_52750 | Max Lysine: 4

HVLGVSKKEAIEKAEHYLAK

>PA14_52800 | Max Lysine: 4

IKPFEKVKQTSFDDHHVDIK

>PA14_52900 | Max Lysine: 4

TKIKTTAVRKGDRYVINGQK

>PA14_53020 | Max Lysine: 4

GGKNAFVKAMNAKAHALGMK

>PA14_53120 | Max Lysine: 4

GVYENAFPKEGRKRAMKWLK

>PA14_53150 | Max Lysine: 4

NPLVIWSTVQLGKRVKHLKK

>PA14_53290 | Max Lysine: 4

EKILQDKLQARVAEGKIVLK

>PA14_53400 | Max Lysine: 4

GYGPMEKAKDGKVLVHGMSK

>PA14_53490 | Max Lysine: 4

DFPFMKRYSKAYLARHPEKK

>PA14_53600 | Max Lysine: 4

CHKVVIEKGKERVDGCAFNK

>PA14_53610 | Max Lysine: 4

SLLLDLIIKSLAKPRTVYKK

>PA14_53670 | Max Lysine: 4

SAILKPGVQVVYYKGKLTDK

>PA14_53950 | Max Lysine: 4

LLKMLERKDKIMGFGHAIYK

>PA14_54080 | Max Lysine: 4

ERKEVYGWLFKSRHKNARDK

>PA14_54320 | Max Lysine: 4

LVEREGQKKIIIGDKGERIK

>PA14_54520 | Max Lysine: 4

DTYSKLGLTAKVKVSQSELK

>PA14_54540 | Max Lysine: 4

DSPYKNLDDLVKALKADPSK

>PA14_54570 | Max Lysine: 4

SAVAYMTEKKLAGAKGEFGK

>PA14_54590 | Max Lysine: 4

KLEASWKEALREEFDKPYMK

>PA14_54850 | Max Lysine: 4

PANMVKQLKTDMRRKHQLPK

>PA14_55070 | Max Lysine: 4

TVADTKRLKDLELENSRLKK

>PA14_55080 | Max Lysine: 4

KGMATDVKYWSRNGDDKVLK

>PA14_55980 | Max Lysine: 4

LKDLKALLNHLKDNPEILGK

>PA14_56110 | Max Lysine: 4

HQAAWQRFDKRLAFLEKGKK

>PA14_56240 | Max Lysine: 4

PKIRIAKFANKRIELQVGDK

>PA14_56300 | Max Lysine: 4

GDKVEVDVAAKGGGSENKSK

>PA14_56510 | Max Lysine: 4

EPVLLKLLDFDLEEKLKPLK

>PA14_56700 | Max Lysine: 4

EQEKLKALQVELGKTNEQLK

>PA14_56790 | Max Lysine: 4

GYSSLSYLKSLPLDKIKIDK

>PA14_56830 | Max Lysine: 4

KDLQKAIDAFLAKPDAETLK

>PA14_56850 | Max Lysine: 4

VTALDMLKKKLGTPLGRQSK

>PA14_56880 | Max Lysine: 4

AQWAKTSATVAELKAALAKK

>PA14_56920 | Max Lysine: 4

KSRRRLTVNKASQHDLKQLK

>PA14_56950 | Max Lysine: 4

DTLEVKRAGKSLKLNPIGLK

>PA14_57010 | Max Lysine: 4

MAAKEVKFGDSARKKMLVGV

>PA14_57050 | Max Lysine: 4

MKPEALEKMTAGIPLKRMGK

>PA14_57060 | Max Lysine: 4

GGKLLLKTDYGGDITLKWDK

>PA14_57170 | Max Lysine: 4

RRSNRKLELEVQVRSKIEKK

>PA14_57260 | Max Lysine: 4

EAAKKFIRIKREVSVEEGDK

>PA14_57340 | Max Lysine: 4

SGLRGKGLKSLVKAPLELLK

>PA14_57425 | Max Lysine: 4

KRQVLKDRRGRVIKDVQVTK

>PA14_57510 | Max Lysine: 4

PRADLKSKAEQAARTVQKVK

>PA14_57560 | Max Lysine: 4

LALHEVGSNNPDGVDIKKKK

>PA14_57590 | Max Lysine: 4

AVKGMLPKNPLGRDMYRKLK

>PA14_57640 | Max Lysine: 4

VIRKVGKLVGKMTYGVSADK

>PA14_57650 | Max Lysine: 4

HAKPGLFGKLLGRKSGGPVK

>PA14_57710 | Max Lysine: 4

VVRKGDEVVALPSGKGSKVK

>PA14_57720 | Max Lysine: 4

GSAKHTDVMKTEGLKQALDK

>PA14_57840 | Max Lysine: 4

DELLSDIKANKAAYKADPQK

>PA14_57850 | Max Lysine: 4

LEDLIGKFLLNSVNKDEAKK

>PA14_57890 | Max Lysine: 4

AGKGRVVVVGMGKSGHIGKK

>PA14_57920 | Max Lysine: 4

SVGKPAYYEQKPAPDKDVTK

>PA14_57950 | Max Lysine: 4

HFDKITNVQVIMEVEKLKQK

>PA14_58060 | Max Lysine: 4

DPLRKALLEAPKHKAHIARK

>PA14_58090 | Max Lysine: 4

YSVKGPWQDPKISFDKPFEK

>PA14_58250 | Max Lysine: 4

NGQVGQKKQFLRSEKPLYLK

>PA14_58330 | Max Lysine: 4

LGKLKMGGMDFSIADATRKK

>PA14_58410 | Max Lysine: 4

KVQNGPIKDSTFKLTYMMHK

>PA14_58630 | Max Lysine: 4

KVMKTGVGPERISYGNTIKK

>PA14_58690 | Max Lysine: 4

CQGLGVEIKVKLIILKLFEK

>PA14_58750 | Max Lysine: 4

ISERRKPQDGRIKMRVSKTK

>PA14_58970 | Max Lysine: 4

QICSRAYRLGLKKSPEFSKK

>PA14_59070 | Max Lysine: 4

NDLKGDLKFIERAVGVQKAK

>PA14_59210 | Max Lysine: 4

LRRRKDVLPSLKGKQRQLLK

>PA14_59290 | Max Lysine: 4

KSMTLKVTLEGLDKLHGGSK

>PA14_59540 | Max Lysine: 4

LKDIYTERDGDSHKTARGKK

>PA14_59580 | Max Lysine: 4

TLIEQFKFEIALLKRHKFAK

>PA14_59590 | Max Lysine: 4

GKSWTKAEVIVGLKSQTWIK

>PA14_59610 | Max Lysine: 4

CYYMQIRKKTRQMLGMIEKK

>PA14_59770 | Max Lysine: 4

RLLKRSVKTVSTQKVSAMRK

>PA14_59830 | Max Lysine: 4

YNSSVEKAAKGKFPRNVVCK

>PA14_59850 | Max Lysine: 4

ENIVIPYKTLTKGVAMFKHK

>PA14_59940 | Max Lysine: 4

ITKHPLLLPYAMKITKMWRK

>PA14_60000 | Max Lysine: 4

IQETYDKKLQSLQELLSKSK

>PA14_60110 | Max Lysine: 4

KVEPAFDGKLKLRIFNTTNK

>PA14_60140 | Max Lysine: 4

KSKVMNSDQVEAMYRKLTEK

>PA14_60190 | Max Lysine: 4

IQLKIEREALKKEDDEATRK

>PA14_60370 | Max Lysine: 4

AVDSEVAGLKLKVVKSTHAK

>PA14_60400 | Max Lysine: 4

MANTPSAKKRAKQAEKRRSH

>PA14_60450 | Max Lysine: 4

LGKDHTLFAKVDGVVKFETK

>PA14_60460 | Max Lysine: 4

IVTGGKQHKVTEGEFLKVEK

>PA14_60490 | Max Lysine: 4

FSHQPRPDFPDKIKDWPKDK

>PA14_60630 | Max Lysine: 4

MKMLKRFIVKKNERGLLYSE

>PA14_60650 | Max Lysine: 4

WKALADRFKAITDKHPRLEK

>PA14_60660 | Max Lysine: 4

SMARKNYRGYLKGDSVRLKK

>PA14_60800 | Max Lysine: 4

KAARLAQEAKQEASHAKAMK

>PA14_60870 | Max Lysine: 4

LAEACKQLRSWHKAKVRVPK

>PA14_60990 | Max Lysine: 4

KEAGKHGFKRAIVPLGNAPK

>PA14_61040 | Max Lysine: 4

VHYVKFHWKSLQGQKNLDPK

>PA14_61150 | Max Lysine: 4

IWNDFERFRRAKLKLKFNDK

>PA14_61220 | Max Lysine: 4

KRGGIERASKQLAAYGKPRK

>PA14_61400 | Max Lysine: 4

DWRLEVAGQRVQIIKKDPKK

>PA14_61430 | Max Lysine: 4

MRKDKKQIIGEEISDESIKL

>PA14_61710 | Max Lysine: 4

QLPILGKGAVERALKQRKHK

>PA14_61820 | Max Lysine: 4

LDSCEKQLQKVARNAKGGDK

>PA14_61990 | Max Lysine: 4

DRAALRDQKERLEKELKQLK

>PA14_62000 | Max Lysine: 4

GIAIAKAFQDKTGIQVKIRK

>PA14_62020 | Max Lysine: 4

LYKGISVGKVTDLHVSKDIK

>PA14_62130 | Max Lysine: 4

EKLRAMMPWIAANKIVDKSK

>PA14_62230 | Max Lysine: 4

AYKIKKPMDFGFLDFTSLDK

>PA14_62350 | Max Lysine: 4

KYKSDVDTDQKSAYGGPYDK

>PA14_62630 | Max Lysine: 4

EGVRGGKYIPLKQNVEKALK

>PA14_62690 | Max Lysine: 4

TSKVKSSLIANKNVSGVDIK

>PA14_62880 | Max Lysine: 4

MALTQEQKKQFKSIGHHLKP

>PA14_62910 | Max Lysine: 4

EKVKTLGLSSIDRELMYKLK

>PA14_62960 | Max Lysine: 4

KKAYRRLAMKYHPDRNPGDK

>PA14_63270 | Max Lysine: 4

KGLDKCPKVVVLGRAPESLK

>PA14_63540 | Max Lysine: 4

RELIGRIKKNPLKRALRVDK

>PA14_63570 | Max Lysine: 4

KVSRAWARKHHNGWLKEVGK

>PA14_63605 | Max Lysine: 4

PKDMMLKVWAEIAETSKPGK

>PA14_63680 | Max Lysine: 4

AKRIAKRLEKDGVIEHFNHK

>PA14_64000 | Max Lysine: 4

GEHVELLIDELLKRGFKAKK

>PA14_64110 | Max Lysine: 4

AFCKGGVNIHYLEKKLGMDK

>PA14_64190 | Max Lysine: 4

QTKASELLGLNRGTLRKKLK

>PA14_64270 | Max Lysine: 4

VSNPVNEKFVADWKAYAKAK

>PA14_64510 | Max Lysine: 4

VFGWKVKGTYKGQALAGSGK

>PA14_64540 | Max Lysine: 4

MKGPEKKRAKIAIDPSSERQ

>PA14_64670 | Max Lysine: 4

KIPRKGGPGICKSDLLVINK

>PA14_64700 | Max Lysine: 4

GMKQKDIAQALGVALPTVKK

>PA14_65000 | Max Lysine: 4

TKLIGSGEKDSVTFDVSKLK

>PA14_65170 | Max Lysine: 4

KEIDYKDLNTLKAYVSETGK

>PA14_65430 | Max Lysine: 4

VQKKLNLFDDGGSLKLEQIK

>PA14_65540 | Max Lysine: 4

LFKGKTPEQAQAALQRLLKK

>PA14_65740 | Max Lysine: 4

KEHLGLPNKDDVKTGIITYK

>PA14_65810 | Max Lysine: 4

AYKALQKGVPVIALKTGVSK

>PA14_66000 | Max Lysine: 4

EQAKGGDLPPALRDKGFKGK

>PA14_66040 | Max Lysine: 4

KSFVVEKGTPGMTVTRLEKK

>PA14_66080 | Max Lysine: 4

PVIAVMVTTASRKFRKQSKK

>PA14_66150 | Max Lysine: 4

PTWGRHLALSKDVLKRRGKK

>PA14_66230 | Max Lysine: 4

SGFKTKGLDRSLKALAALPK

>PA14_66240 | Max Lysine: 4

LKKTCTYQPTEEDRKLFDLK

>PA14_66290 | Max Lysine: 4

IKGYGTGSGEAKNIAHNVKK

>PA14_66320 | Max Lysine: 4

PQTLMKYAGLALHKAKANGK

>PA14_66400 | Max Lysine: 4

QSLDKLAERKLAEADQKVVK

>PA14_66550 | Max Lysine: 4

EGGSSKDFRKSKAMLYDNPK

>PA14_66580 | Max Lysine: 4

NRAAALAAVKKLPSKVQAGK

>PA14_66620 | Max Lysine: 4

VPWDQALDLVLKTKGLDKRK

>PA14_66630 | Max Lysine: 4

FQPPVKIDLTVRQKGNKVVK

>PA14_66650 | Max Lysine: 4

TQPGEEDAKAKHGVAQGAKK

>PA14_66660 | Max Lysine: 4

LIKKKANTLLGIDISSTSVK

>PA14_66830 | Max Lysine: 4

KKLILAATSAGAVMVPGKPK

>PA14_66900 | Max Lysine: 4

VPESQKAKKVAEVFHSVAAK

>PA14_66960 | Max Lysine: 4

TKRLKNLGSDVGEAIKGFRK

>PA14_67030 | Max Lysine: 4

VVRKRGKAEREAKARALLAK

>PA14_67050 | Max Lysine: 4

VVGQTLLVRKELEGKIKSYK

>PA14_67065 | Max Lysine: 4

ETKGFGKIFYRRRRRRVKRK

>PA14_67180 | Max Lysine: 4

GNPPALTELGKLYIYVAKKK

>PA14_67190 | Max Lysine: 4

GVEKDTQKALDLYQKMIDLK

>PA14_67210 | Max Lysine: 4

GNPPALTELGKLYIYVAKKK

>PA14_67460 | Max Lysine: 4

AACSKINQENYSKLKAGMSK

>PA14_67560 | Max Lysine: 4

HGKTTLVDKLLKLSGTLDRK

>PA14_67580 | Max Lysine: 4

MKLIVKTFQEITIKSRPVRK

>PA14_67600 | Max Lysine: 4

LIKDHDVKWVDLRFTDTKGK

>PA14_67750 | Max Lysine: 4

NIPADKLNSRMTELEKYKGK

>PA14_67810 | Max Lysine: 4

GGRPFDVELKRAIIKVKSVK

>PA14_67850 | Max Lysine: 4

AKKYRIGAYKGDAIAEFLGK

>PA14_68130 | Max Lysine: 4

EVATRKVALAKAEADYKRRK

>PA14_68200 | Max Lysine: 4

VEFDQGGKAISLEEKPLKPK

>PA14_68260 | Max Lysine: 4

PKGQGALLFKKLVEERLPGK

>PA14_68290 | Max Lysine: 4

FVYKDMRLKECPKVLLESGK

>PA14_68330 | Max Lysine: 4

MSTEKTKLGVHSEAGKLRKV

>PA14_68340 | Max Lysine: 4

QCKKFAEESGAKLTLTEDPK

>PA14_68450 | Max Lysine: 4

YYRHDNGHHYGRYKKWKRHK

>PA14_68580 | Max Lysine: 4

NFAQKKVLIAGMRYAGEMKK

>PA14_68630 | Max Lysine: 4

EKDDDKVRLDKWLWAARFFK

>PA14_68820 | Max Lysine: 4

KSLGRMNVAEKRKPQDGRVK

>PA14_68900 | Max Lysine: 4

DELIKPVFDAYTSKTGVKVK

>PA14_69150 | Max Lysine: 4

REIGKLLAQLKEPEPPKGLK

>PA14_69170 | Max Lysine: 4

RYAKASKWRDGQTDCMFDKK

>PA14_69200 | Max Lysine: 4

QGKLKVCKLNIDENQDTPPK

>PA14_69340 | Max Lysine: 4

FIARFKAKATKARQAQSRIK

>PA14_69450 | Max Lysine: 4

GDKLLDAPLAKIGGKGLFVK

>PA14_69520 | Max Lysine: 4

LIRLKKSHGEKVDDALHGAK

>PA14_69690 | Max Lysine: 4

ARFVQDKRLTVKKSIRVETK

>PA14_69870 | Max Lysine: 4

LKNRKTGELTTARKQIAEGK

>PA14_69950 | Max Lysine: 4

RALGWLKRKLKLSEILELGK

>PA14_69970 | Max Lysine: 4

TGLLGAPKVGDKAEWGKRAK

>PA14_70040 | Max Lysine: 4

GIPLKAMKWLLEKHAPLAIK

>PA14_70050 | Max Lysine: 4

AGKKAMEKTPAGQYVQVGGK

>PA14_70140 | Max Lysine: 4

ERSIKDKFLPLVIEALKGWK

>PA14_70390 | Max Lysine: 4

NQKFKFMDDFTHYLSKQRRK

>PA14_70420 | Max Lysine: 4

LIDALAVLKKRGALKGNPLK

>PA14_70470 | Max Lysine: 4

VIGREKHLYSIYQKMRGKRK

>PA14_70570 | Max Lysine: 4

KWLQPLDIEVAWLAGKLKGK

>PA14_70600 | Max Lysine: 4

KNPQTGQPVKIKASNTVAFK

>PA14_70790 | Max Lysine: 4

VSDYIGKPFSNDQLVAKIKK

>PA14_70830 | Max Lysine: 4

LQKRMQRVDDLYAKLYKLEK

>PA14_70860 | Max Lysine: 4

MSRKMKDVELQAFEQKYGYK

>PA14_71000 | Max Lysine: 4

RKKLIDEKLELVGLSQWRDK

>PA14_71030 | Max Lysine: 4

ENKLMDAVLNENKKPEEAAK

>PA14_71160 | Max Lysine: 4

LTAVKRAIKRKQWVVFFGWK

>PA14_71330 | Max Lysine: 4

NLSLLKNGKVKGIRLDTLEK

>PA14_71340 | Max Lysine: 4

AERRSYHYCSQGKSKKQARK

>PA14_71410 | Max Lysine: 4

TVSMTMDGKQGSKKLMGRIK

>PA14_71530 | Max Lysine: 4

LLPGFKGAKPYHQAYQKGVK

>PA14_71580 | Max Lysine: 4

SKGGLKQSDRKMPAVMYSAK

>PA14_71780 | Max Lysine: 4

HLKSVKRSLRSLRLSPKVMK

>PA14_71800 | Max Lysine: 4

EKLKVLQALKPISGLDVQDK

>PA14_72380 | Max Lysine: 4

GSGKGELARAIHTWSKRAKK

>PA14_72480 | Max Lysine: 4

VLMTKADKLAFGAAKNALLK

>PA14_72520 | Max Lysine: 4

MSAVKITQKIKGFKVVNEAE

>PA14_72540 | Max Lysine: 4

IALQDASQDIWDKKYRLKSK

>PA14_72940 | Max Lysine: 4

YRKLKALSMLLAELGVKPEK

>PA14_73120 | Max Lysine: 4

SGIKTLADLKGKRISVGAPK

>PA14_73260 | Max Lysine: 4

KGDFNDEIDAGIKAGIEKFK

>PA14_73360 | Max Lysine: 4

KRLTLLDSNGKKTRFLTQVK

>PA14_73420 | Max Lysine: 4

LGLVIGKKNVKLAVQRNRLK

>PA14_00020 | Max Lysine: 3

KLVDGKFPDYERVLPRGGDK

>PA14_00070 | Max Lysine: 3

PDDGCDCRKPKPGMLRQIGK

>PA14_00160 | Max Lysine: 3

LEKMLAKGVDNALLRFGLGK

>PA14_00170 | Max Lysine: 3

VAHGDAKSSKVIGRSINEIK

>PA14_00210 | Max Lysine: 3

GQPAYGIFRQGKVYIDPKTK

>PA14_00250 | Max Lysine: 3

RVLELTDGKKCPVVYDSVGK

>PA14_00450 | Max Lysine: 3

CGGAKIYLKREELNHTGAHK

>PA14_00470 | Max Lysine: 3

MSNHHTYKKIELVGSSKTSI

>PA14_00550 | Max Lysine: 3

GGIVLTLLLSLKDKLHALLK

>PA14_00560 | Max Lysine: 3

EIKQMMLQKALPLTLGGLGK

>PA14_00580 | Max Lysine: 3

YFKGESKEAQAQALEADLEK

>PA14_00620 | Max Lysine: 3

KRLCAADGRTPVTSYAKALK

>PA14_00640 | Max Lysine: 3

VYNVPWSIKSRDGEEKWLLK

>PA14_00670 | Max Lysine: 3

RTLGNLSAILKKAAAHAEAK

>PA14_00720 | Max Lysine: 3

ATALAAPKPCEELKAEIETK

>PA14_00740 | Max Lysine: 3

KLYDQDNRELARGRKGRCAK

>PA14_00780 | Max Lysine: 3

VPPGKVLESGFLYVGSPAKK

>PA14_00790 | Max Lysine: 3

ALAGLTDSAKAQMKQAAEAK

>PA14_00830 | Max Lysine: 3

AWQQKLSASLPKPAAGTPLK

>PA14_00875 | Max Lysine: 3

GERPLLMPGKKTLFQRVLTK

>PA14_01110 | Max Lysine: 3

RSSKGGTPANFNEIRMEDKK

>PA14_01150 | Max Lysine: 3

YVARQLENAEKQLPGFKLHK

>PA14_01190 | Max Lysine: 3

YYFKEASLTLSRNLRVLKPK

>PA14_01250 | Max Lysine: 3

VLVGFALTLLKLVLKAARLK

>PA14_01300 | Max Lysine: 3

LLFLFIVIKCIRGGKPAPAK

>PA14_01460 | Max Lysine: 3

IPDLAVQMKPLGDGFIKLIK

>PA14_01480 | Max Lysine: 3

ESIYTAILEKRLAPAAKLSK

>PA14_01510 | Max Lysine: 3

VLIGPEKALKAVQDIVEQVK

>PA14_01550 | Max Lysine: 3

YVVKGKLAGGLFGTVRFSEK

>PA14_01600 | Max Lysine: 3

PSTGEAIHKVPLADGKTLQK

>PA14_01750 | Max Lysine: 3

KTLYPVWARLTPEKLELATK

>PA14_01760 | Max Lysine: 3

PKELKIQALTTVAGNVPLEK

>PA14_01800 | Max Lysine: 3

TAKVARQFPKVTFEHATGYK

>PA14_01870 | Max Lysine: 3

FGMDHEKRKIFRADLLRDSK

>PA14_01890 | Max Lysine: 3

SKTLKELEEILGARLFERSK

>PA14_01940 | Max Lysine: 3

QAQSKLADLNYQRQKALLPK

>PA14_01960 | Max Lysine: 3

DGDRVVRAGVNSLKPGQKIK

>PA14_02060 | Max Lysine: 3

LHFNPQVKYDLGKALGYEPK

>PA14_02140 | Max Lysine: 3

VRSIQAIANLDKAALKAAYK

>PA14_02190 | Max Lysine: 3

KIYFFPRNGRVLVKSLVELK

>PA14_02200 | Max Lysine: 3

MPAPLKKRFFLRGTGPNAGK

>PA14_02220 | Max Lysine: 3

ARIAKGVNELVAAHIAVKMK

>PA14_02260 | Max Lysine: 3

LKSAGYEVDEAADGKEGLGK

>PA14_02270 | Max Lysine: 3

SAISQLADKDMSFALDSKQK

>PA14_02360 | Max Lysine: 3

QLLEKEFANDGIAVKWSFFK

>PA14_02500 | Max Lysine: 3

ALVKGVQFARQKRQDRQFQK

>PA14_02510 | Max Lysine: 3

EQKDPLVVSIDGAGKLFVNK

>PA14_02630 | Max Lysine: 3

LVPLLDRFGRKKAEGAPPLK

>PA14_02700 | Max Lysine: 3

EVLIRTVRRYWQILGKPQKK

>PA14_02720 | Max Lysine: 3

VAGEYNGEMAKVKAMVDVGK

>PA14_02740 | Max Lysine: 3

ATTLELPKDQLIQHALKQMK

>PA14_02760 | Max Lysine: 3

AQKADRKGNVLLWGILGVQK

>PA14_02770 | Max Lysine: 3

GSAKEVLIILKQSHRTFVDK

>PA14_02850 | Max Lysine: 3

ELKLHLSAAKNNGVSRDEIK

>PA14_02890 | Max Lysine: 3

RNNNLASAKKRALVFGPTLK

>PA14_03010 | Max Lysine: 3

MSKPDPAVVEESAPRGKGRK

>PA14_03050 | Max Lysine: 3

KARLPAEVAEKLVTGPSLEK

>PA14_03166 | Max Lysine: 3

VNISTVYPDKYFDQSCILKK

>PA14_03200 | Max Lysine: 3

RKYMVLAAMRLAMLIKIIRK

>PA14_03240 | Max Lysine: 3

GQPTGQRVHKPVVITKVFDK

>PA14_03290 | Max Lysine: 3

LRRWVKQLEAERQGVTPKSK

>PA14_03310 | Max Lysine: 3

PKPRSTKESLHILVPRWDAK

>PA14_03340 | Max Lysine: 3

YIEAIESKVNKRFTFGSLNK

>PA14_03370 | Max Lysine: 3

KMLLKEGCPECHASTPAGSK

>PA14_03380 | Max Lysine: 3

RLKKRHDPMIALEAAQSFTK

>PA14_03450 | Max Lysine: 3

PYIELAEEIAKRVPGDFPKK

>PA14_03490 | Max Lysine: 3

AKASPDAYAAMLGLEKALAK

>PA14_03560 | Max Lysine: 3

KKLENRWEYLSQALRDLNSK

>PA14_03710 | Max Lysine: 3

GQVVQIERKEKFRLQQPAVK

>PA14_03720 | Max Lysine: 3

FGTGYSSLMRLKRLPVHKLK

>PA14_03800 | Max Lysine: 3

MKSRKINKSRLALAITAGTL

>PA14_03810 | Max Lysine: 3

ALRVLEESRDAKGRKLVVHK

>PA14_03860 | Max Lysine: 3

NGVVRGKRIERNSLNKVFEK

>PA14_03880 | Max Lysine: 3

MTTKLDQLTSWLKERKITEV

>PA14_03950 | Max Lysine: 3

ILIIPIILFNKNQAKELEGK

>PA14_03980 | Max Lysine: 3

HYEENEVKANSKGVLVLEPK

>PA14_04100 | Max Lysine: 3

KVYFITPADGATVDKTFTVK

>PA14_04180 | Max Lysine: 3

IVKRIKGDIYEFRDATGSMK

>PA14_04250 | Max Lysine: 3

RPKVLLLDESLSALDLKLRK

>PA14_04270 | Max Lysine: 3

NKGFEGLAYDAEGKRLFVAK

>PA14_04310 | Max Lysine: 3

HKAEFDGAVASSEATAKRLK

>PA14_04320 | Max Lysine: 3

LKREDLQPVFSFKIRGAYNK

>PA14_04410 | Max Lysine: 3

MLNTLRKIVQEVNSAKDLKA

>PA14_04440 | Max Lysine: 3

VALQMVLDLKPKGSREVPGK

>PA14_04510 | Max Lysine: 3

LETPEDKQWREGKLPEALRK

>PA14_04610 | Max Lysine: 3

ATSKVSKQPVEGPLWMQRIK

>PA14_04650 | Max Lysine: 3

TGPKKALEDAGATVRILSAK

>PA14_04670 | Max Lysine: 3

RKPAGSISKARYLRLAEEIK

>PA14_04680 | Max Lysine: 3

HAMLKLSGWKIEGQLPALDK

>PA14_04700 | Max Lysine: 3

AEDAVSDLAKEQSKAEQGDK

>PA14_04760 | Max Lysine: 3

KKNPLFSLEQRVALAQEVTK

>PA14_04780 | Max Lysine: 3

AGLKVLLVEEGPLKTSSDFK

>PA14_04980 | Max Lysine: 3

KLEVLADQKTLFPNVVETLK

>PA14_05000 | Max Lysine: 3

SLDRLLLFFPDPWHKKRHHK

>PA14_05010 | Max Lysine: 3

LKKLGILLLGAGCAWAAQAK

>PA14_05040 | Max Lysine: 3

AKLSAPDGRIVRTWKTRLPK

>PA14_05050 | Max Lysine: 3

MIKLEQLVLASHNAGKLKEL

>PA14_05080 | Max Lysine: 3

RKPGWWDSCIGPGKPIDTRK

>PA14_05120 | Max Lysine: 3

EGKANAHLLAFLGKAFGVAK

>PA14_05150 | Max Lysine: 3

LSVKPQAMKAVCQALAPALK

>PA14_05220 | Max Lysine: 3

GRAKGYRVVLVVPDKMSTEK

>PA14_05330 | Max Lysine: 3

PTEMYKLTAMLEKHGHQVLK

>PA14_05390 | Max Lysine: 3

GILPIAEIATPLEKLVKEYK

>PA14_05420 | Max Lysine: 3

DGMAEQVGLSKYHLLRAFKK

>PA14_05460 | Max Lysine: 3

EVALKMSYHFWLNSGRPRKK

>PA14_05510 | Max Lysine: 3

AADYKIDKEGQHAFIEFRIK

>PA14_05540 | Max Lysine: 3

PALCATMLKPIEKGDHGEHK

>PA14_05550 | Max Lysine: 3

LKADQAQLQLTKDTLGTYQK

>PA14_05580 | Max Lysine: 3

HHYWSRKQLFRAKIQAWLCK

>PA14_05590 | Max Lysine: 3

KTEAGHEKLLATARNLAGYK

>PA14_05690 | Max Lysine: 3

QRKALLSHEDVKQRAWQTLK

>PA14_05775 | Max Lysine: 3

LTILVIFIFNYLKSISYKLK

>PA14_05820 | Max Lysine: 3

RELAAKADILLENFKVGGLK

>PA14_05840 | Max Lysine: 3

GSMVTRAKKVDGGYRLSGSK

>PA14_05860 | Max Lysine: 3

TMTCHVRNKITRKSILTIDK

>PA14_05880 | Max Lysine: 3

TPDKPAEKPPARDAEPMARK

>PA14_05960 | Max Lysine: 3

GFKTLAEGQKVSFEVVQGQK

>PA14_05970 | Max Lysine: 3

KKDGERWVRFDVVLPTADSK

>PA14_05990 | Max Lysine: 3

FAMVAKPMAKPLLDFFGYRK

>PA14_06060 | Max Lysine: 3

YVVKPFSPREVAARVKAILK

>PA14_06070 | Max Lysine: 3

YVAAPIKDAGKIIGVVSVAK

>PA14_06160 | Max Lysine: 3

FLPLRGTKYDAPFGKVSHHK

>PA14_06340 | Max Lysine: 3

LCALLSGERLASLGLKKGQK

>PA14_06620 | Max Lysine: 3

AKLPDAPAGTKGISLFIVPK

>PA14_06640 | Max Lysine: 3

IIRTKAEPQADGSYKISGTK

>PA14_06680 | Max Lysine: 3

DEMLVSSRILKKTGLRLAQK

>PA14_06740 | Max Lysine: 3

DPEVLFKNKGCVACHAIDTK

>PA14_06810 | Max Lysine: 3

LAEFLKWSSKIDTNQWPPNK

>PA14_06930 | Max Lysine: 3

YPWLVAEKRLIRAALDAGKK

>PA14_06990 | Max Lysine: 3

VLLRQFPGVITAKLFGYKPK

>PA14_07030 | Max Lysine: 3

DGVRKDFAAVEDACEACHKK

>PA14_07070 | Max Lysine: 3

KPVKGPRVETFLDVFRGSAK

>PA14_07090 | Max Lysine: 3

DKIADQISDAVLDAIIAKDK

>PA14_07130 | Max Lysine: 3

IKTAIDTARKSDQPTLICCK

>PA14_07140 | Max Lysine: 3

LIALRRVEDDEGRVKWWRKK

>PA14_07210 | Max Lysine: 3

SGSSGKPGGKDWTRWTCDSK

>PA14_07240 | Max Lysine: 3

SPCLRKSGKGCRYSQATLLK

>PA14_07250 | Max Lysine: 3

QFKGRDAYQKHWQACTEMCK

>PA14_07300 | Max Lysine: 3

AGDLVENWRLWKEVNSKAAK

>PA14_07330 | Max Lysine: 3

WFLKCVKEHYFDFNGRARRK

>PA14_07450 | Max Lysine: 3

QKGGLDITKWLQWFLETLLK

>PA14_07500 | Max Lysine: 3

ILARKNGPMPKRIDELYGLK

>PA14_07550 | Max Lysine: 3

SEIKSRLLDAVKVAMRAQDK

>PA14_07570 | Max Lysine: 3

AVVETLLIKCRRALKQTGLK

>PA14_07580 | Max Lysine: 3

MLRVAGKKLAILTLLGDVGK

>PA14_07660 | Max Lysine: 3

KNKSTVNRQRFLRRYREHIK

>PA14_07700 | Max Lysine: 3

LGNHDLHLLAVAHKSERLKK

>PA14_07730 | Max Lysine: 3

LKLKFGLESRFSLHQGDALK

>PA14_07760 | Max Lysine: 3

VRTPGGFIILKLEEKRGGSK

>PA14_07770 | Max Lysine: 3

TPTLKYLYTKYDLDLDSQGK

>PA14_07800 | Max Lysine: 3

LRKLDRRAQRRAIASFGKGK

>PA14_07810 | Max Lysine: 3

SASADKDASAADSASKPDVK

>PA14_07840 | Max Lysine: 3

QQLAVSNKTISTHKARLMQK

>PA14_07860 | Max Lysine: 3

QKSYDGETLIVKDLNLDIRK

>PA14_07930 | Max Lysine: 3

VDTLKWLKRNGVEMALITNK

>PA14_07960 | Max Lysine: 3

GVSQNTIHKLTSGKAQSTRK

>PA14_08070 | Max Lysine: 3

TPGAQASAKPNVPVLLTSKK

>PA14_08150 | Max Lysine: 3

TVKTRYQLPGSGEVKSVELK

>PA14_08210 | Max Lysine: 3

NPADQIEDPCLSDTSRKYKK

>PA14_08220 | Max Lysine: 3

ESALVRKEITWTRVPAGRKK

>PA14_08350 | Max Lysine: 3

VVAAAGGKVAKHGNRAVSGK

>PA14_08400 | Max Lysine: 3

LRFPAPVKLGMSLLAKVMTK

>PA14_08430 | Max Lysine: 3

GKPGDQPKGMMLVGVQGAGK

>PA14_08450 | Max Lysine: 3

ASLETVKDNLARLFKSEPGK

>PA14_08510 | Max Lysine: 3

PGAANKVKTLIDEEGNPRLK

>PA14_08560 | Max Lysine: 3

ILVEAELVAKLKRGQPLRIK

>PA14_08620 | Max Lysine: 3

LGVREAGLKWPNDVLQGGKK

>PA14_08640 | Max Lysine: 3

QSPLRAKEIAPLELYKDGQK

>PA14_08695 | Max Lysine: 3

MNAKAEAKESRFDLLKWLLV

>PA14_08710 | Max Lysine: 3

VPTEEVVEMRNGQKRKSERK

>PA14_08840 | Max Lysine: 3

HKRVLDIVQPTDKTVDALMK

>PA14_08850 | Max Lysine: 3

GQMVDVTGESKGKGFAGTIK

>PA14_08930 | Max Lysine: 3

SQVKRDIARVKTVLNQQAGK

>PA14_09040 | Max Lysine: 3

GLGKTGGRGHKGLTSRSGGK

>PA14_09050 | Max Lysine: 3

TAISPQLEQLKKEGESGRRK

>PA14_09100 | Max Lysine: 3

RYIGPKCKLSRREGTDLFLK

>PA14_09130 | Max Lysine: 3

NMAVSLFEHELIKTTLPKAK

>PA14_09150 | Max Lysine: 3

VTHDITPYTRAKIFSQVGKK

>PA14_09160 | Max Lysine: 3

AKGIALCEQHKDFVSRDILK

>PA14_09195 | Max Lysine: 3

VGFFGMVPFIIYAEKKRRMK

>PA14_09240 | Max Lysine: 3

APSALKLKQYLHACGLAAFK

>PA14_09340 | Max Lysine: 3

MKTETKVIKGRQGIARNRHT

>PA14_09400 | Max Lysine: 3

ANREREKEEWAAASRPKTEK

>PA14_09470 | Max Lysine: 3

DANELREKNRATVEKYMNTK

>PA14_09530 | Max Lysine: 3

YATGSAKKDAGGFAGYPPVK

>PA14_09600 | Max Lysine: 3

SKVKEVPPDSDSLAIIRSGK

>PA14_09660 | Max Lysine: 3

AFKIPRTVVFSELPKTSTGK

>PA14_09710 | Max Lysine: 3

FLKYAAESNLKRVVLECGGK

>PA14_09730 | Max Lysine: 3

LKALDEAGKAALAEIVARLK

>PA14_09760 | Max Lysine: 3

AEIETLKAIQEDFRKAIEEK

>PA14_09790 | Max Lysine: 3

VGIEIEITRLVGKFKLSQNK

>PA14_09850 | Max Lysine: 3

GKEWERDIELKYVVQSGRFK

>PA14_09950 | Max Lysine: 3

EWLSEAAVAAKKEADGFVVK

>PA14_09960 | Max Lysine: 3

KNEGYVSSEKGHRGGWRLAK

>PA14_10040 | Max Lysine: 3

AKDSYLVKGLTAASGSPAFK

>PA14_10220 | Max Lysine: 3

NDTWKTRVQGMQETLDTRKK

>PA14_10240 | Max Lysine: 3

KEEGAHIEKGDEVLDVETDK

>PA14_10340 | Max Lysine: 3

RLGLGTRAVKLDLGDLGKLK

>PA14_10370 | Max Lysine: 3

QKSGRAEAIWFPFTDKPWLK

>PA14_10400 | Max Lysine: 3

AISRCLSRHSKKHRPIPLTK

>PA14_10410 | Max Lysine: 3

RAVKAGKRLDTNRGTSSQQK

>PA14_10500 | Max Lysine: 3

IVWVAYAVVFFGTLIKRKVK

>PA14_10530 | Max Lysine: 3

KPVKAYDQEGLVMHCSSFSK

>PA14_10590 | Max Lysine: 3

IQRAWVERKIADGRVLKGHK

>PA14_10630 | Max Lysine: 3

GRRIMEAAGIKKYSMELGGK

>PA14_10650 | Max Lysine: 3

PEFQQPPYQKPPVKPVLFIK

>PA14_10730 | Max Lysine: 3

KIVVCWGGHSISSEEYKYTK

>PA14_10770 | Max Lysine: 3

GPDGGVVGASKTVRDISAKK

>PA14_10790 | Max Lysine: 3

VKIAYAILSGLEQQAKVPLK

>PA14_10820 | Max Lysine: 3

LLHDIGKMAVPDPILNKPGK

>PA14_10910 | Max Lysine: 3

AQRPAPRAEDAKGKRLALLK

>PA14_10920 | Max Lysine: 3

TREFWKDLQPIPDCFKPDAK

>PA14_11000 | Max Lysine: 3

QSKEKLCWETDTGNGGYTHK

>PA14_11100 | Max Lysine: 3

SGAKAGLELKAEGAIDINDK

>PA14_11240 | Max Lysine: 3

SIAFKWGFSDSAHFSRAFKK

>PA14_11250 | Max Lysine: 3

DEPQHAVMVCKKILHTFGIK

>PA14_11270 | Max Lysine: 3

DPDDSSSDIKLDGAKQRGTK

>PA14_11290 | Max Lysine: 3

EKIVLAHGVSTVSLVKHDDK

>PA14_11310 | Max Lysine: 3

KGPSGSGKTTLLGLLGGVQK

>PA14_11320 | Max Lysine: 3

AASTDADKAAVAKARAQLEK

>PA14_11410 | Max Lysine: 3

KGGDLRVYIATGKLDLGDVK

>PA14_11510 | Max Lysine: 3

PLQRGLNKHNRRYLATKAGK

>PA14_11560 | Max Lysine: 3

VESDTATLGKTQGKDQAHNK

>PA14_11620 | Max Lysine: 3

LDPNQKHQVRQLVKNLSESK

>PA14_11650 | Max Lysine: 3

PDEDVLVDARRGLKVKPQGK

>PA14_11750 | Max Lysine: 3

GMLSYPESSRKFLEKRLRRK

>PA14_11810 | Max Lysine: 3

KRIAKIAFTGSTPVGSHILK

>PA14_11890 | Max Lysine: 3

LEEGKGVRIVESDELRKLGK

>PA14_11900 | Max Lysine: 3

TDDLSKAFELAKEEVRQREK

>PA14_11970 | Max Lysine: 3

AYYLEEKGSHASLGYTEKRK

>PA14_12010 | Max Lysine: 3

RGGKGLIERISREAKVPVIK

>PA14_12060 | Max Lysine: 3

KSGTAQVVAIKQNERYDRSK

>PA14_12080 | Max Lysine: 3

RKQSILDAISRPAERVKQWK

>PA14_12090 | Max Lysine: 3

LSFAAAKKLGYAETGTARVK

>PA14_12330 | Max Lysine: 3

LKSRKAHIRPRGANQQRYVK

>PA14_12390 | Max Lysine: 3

GDKELEITLNAAEKAFAALK

>PA14_12450 | Max Lysine: 3

EQEEKFARLVTAVGKYWICK

>PA14_12540 | Max Lysine: 3

MAKAPPSEPKLLLFNKPFDV

>PA14_12570 | Max Lysine: 3

KQLEDDGYIVRQVALLDRKK

>PA14_12630 | Max Lysine: 3

NFWRSTYAEVRKDLKGRYPK

>PA14_12670 | Max Lysine: 3

LKPGQALKVMTWNIQYLAGK

>PA14_12710 | Max Lysine: 3

KLKVIAAILPGPWHPAVVAK

>PA14_12750 | Max Lysine: 3

NLGFTVKLKPYIQRADGSAK

>PA14_12810 | Max Lysine: 3

AVTALKKVVPGSILEAADGK

>PA14_12870 | Max Lysine: 3

VKYLWFRADGKLPDVPALHK

>PA14_12920 | Max Lysine: 3

IDPTKVPQADGLYEKAIGEK

>PA14_12980 | Max Lysine: 3

LKKRAAAAGVDEETYAEEPK

>PA14_13090 | Max Lysine: 3

INKLCGSGMKAVMLAHDLLK

>PA14_13170 | Max Lysine: 3

LAKIIRLVEDAQAAKAPIQK

>PA14_13190 | Max Lysine: 3

HRVAIPMTVLEELDKLKTGK

>PA14_13230 | Max Lysine: 3

VIEQVQLLEKLGGKSGHYRK

>PA14_13250 | Max Lysine: 3

FDACNFVMDYLKTRAPFWKK

>PA14_13340 | Max Lysine: 3

TAEEFQRQKAKIVAAILASK

>PA14_13420 | Max Lysine: 3

QPGYKATWHAMFKGESDVPK

>PA14_13500 | Max Lysine: 3

GNWKRTVDAPQFGTDVHGKK

>PA14_13510 | Max Lysine: 3

LYPSRQYLDAKIKTWVEYLK

>PA14_13560 | Max Lysine: 3

VKKGDLLMQIDPDHYRIAVK

>PA14_13740 | Max Lysine: 3

LKIQVSRLGTLLKREAPAEK

>PA14_13770 | Max Lysine: 3

NDIADAKASFRDQAVIFKRK

>PA14_13830 | Max Lysine: 3

RFDLSQYTWKAGSSQMLSKK

>PA14_13890 | Max Lysine: 3

QTHIPASKDGTRTTARSKGK

>PA14_13940 | Max Lysine: 3

FNTKVKQIVNEATAFSTARK

>PA14_13970 | Max Lysine: 3

PSFPMKQMTFADAEYAGKRK

>PA14_13990 | Max Lysine: 3

KCQWIEQEYDGLIPSLKVRK

>PA14_14000 | Max Lysine: 3

REHKRELVLALHAEGAFKGK

>PA14_14060 | Max Lysine: 3

AVGKPDAKAGELPVVYIQLK

>PA14_14100 | Max Lysine: 3

QRLKLAKPDQYVVLPEVISK

>PA14_14160 | Max Lysine: 3

MGYLKNHWWKRWIRRRGCKL

>PA14_14230 | Max Lysine: 3

TGAEGGGYAVFAGKLREKLK

>PA14_14300 | Max Lysine: 3

NSRYPFLPKRGYAMKALILK

>PA14_14340 | Max Lysine: 3

HASLDEATRGSRRKWKNLGK

>PA14_14360 | Max Lysine: 3

ERVRKFHSEVVKVGGSTELK

>PA14_14370 | Max Lysine: 3

RKGLDVPDLLAMFEKVRGEK

>PA14_14520 | Max Lysine: 3

KDKRTWHDRYSESQVVRLPK

>PA14_14550 | Max Lysine: 3

FPPLLCDSKGEAMYAEKIGK

>PA14_14600 | Max Lysine: 3

AMRKIKEEGVTFASPVDGAK

>PA14_14650 | Max Lysine: 3

GKKVATALQQADTGNPANLK

>PA14_14660 | Max Lysine: 3

EVVAVQPVKETIKTPRQVCK

>PA14_14680 | Max Lysine: 3

GSHEFLEKGHIVAGNTKCFK

>PA14_14690 | Max Lysine: 3

TYEVRMAWLAAQGKPTKMEK

>PA14_14700 | Max Lysine: 3

NKGKRHPTLGNNVVVGAGAK

>PA14_14820 | Max Lysine: 3

IKPDAVSKNVIGEILTRFEK

>PA14_14830 | Max Lysine: 3

GVDDFDAMTNVGKALREKLK

>PA14_14880 | Max Lysine: 3

NIPIRIGVNAGSLEKDLQKK

>PA14_14900 | Max Lysine: 3

LNPAGQPDPAKVAELAGKLK

>PA14_15100 | Max Lysine: 3

ERFSVGSKHYREQRITLKNK

>PA14_15120 | Max Lysine: 3

HKNGMMSMQKVDSVDVAPGK

>PA14_15200 | Max Lysine: 3

SEKNLVHLFASNLKRLAEEK

>PA14_15230 | Max Lysine: 3

AFEALKEKLAGEGLFASERK

>PA14_15340 | Max Lysine: 3

AEDKFLGRLAGVADPEEKRK

>PA14_15360 | Max Lysine: 3

KAELRNQARLMVDSKLSDRK

>PA14_15430 | Max Lysine: 3

ERQLEQIQVDKVFTDKASGK

>PA14_15460 | Max Lysine: 3

KITGMTCDSCAAHVKEALEK

>PA14_15480 | Max Lysine: 3

THCEEASSLAEHKLKDVREK

>PA14_15510 | Max Lysine: 3

MDDEKRVTRKSSPPIKVYCL

>PA14_15540 | Max Lysine: 3

GGGSSSAGSKGGDKGGAGDK

>PA14_15590 | Max Lysine: 3

AYDKRGEQYLLIKSPPASGK

>PA14_15680 | Max Lysine: 3

MRVTKLISNGKGYPTVKGVR

>PA14_15700 | Max Lysine: 3

SFLTNIFDKFHKLGPENRPK

>PA14_15740 | Max Lysine: 3

PVGKDSMSMKTRWQDNGEDK

>PA14_15770 | Max Lysine: 3

WKVELVVADELIHASVKALK

>PA14_15780 | Max Lysine: 3

KASDAQLKALGAMAVVRPGK

>PA14_15830 | Max Lysine: 3

LIRLGLSPTEKVARMKRLRK

>PA14_15870 | Max Lysine: 3

GAKIGKYCIIGANALIPEGK

>PA14_15890 | Max Lysine: 3

YPCVVKPIMSSSGKGQSVLK

>PA14_15920 | Max Lysine: 3

GLYVRLKLAETPVFAKAMAK

>PA14_15980 | Max Lysine: 3

RQAELVRGRLHGKVLAAKLK

>PA14_15990 | Max Lysine: 3

KIKPLEGALADARQAAGGRK

>PA14_16040 | Max Lysine: 3

TLEQVNLRQGVVKVFGKGSK

>PA14_16070 | Max Lysine: 3

AHELVLKAIENGKHVVTANK

>PA14_16090 | Max Lysine: 3

HGRNGKAVAELLDAFKASGK

>PA14_16100 | Max Lysine: 3

KAPGKTPTIPPSRNAFTCLK

>PA14_16160 | Max Lysine: 3

EDGGAGLILKVRAEENKGFK

>PA14_16190 | Max Lysine: 3

LILKLRELAERLRELKDSVK

>PA14_16320 | Max Lysine: 3

LLLGDGKEVVKGALITTQYK

>PA14_16330 | Max Lysine: 3

NEFIIYEIDGKRGILDAKGK

>PA14_16350 | Max Lysine: 3

LKLGIHGLVSKRGMLDDLPK

>PA14_16360 | Max Lysine: 3

NALYSEDAKHLLKRLGVELK

>PA14_16430 | Max Lysine: 3

GVRKAYLEQHDKVLELYREK

>PA14_16590 | Max Lysine: 3

MGDGSDGSLLGKLWSAGKRK

>PA14_16600 | Max Lysine: 3

ADGVRLRAWWLPAKKGVPVK

>PA14_16610 | Max Lysine: 3

FTMLKSLPGEDVKLSLEPAK

>PA14_16660 | Max Lysine: 3

GEWKELPAKQVELEATVRVK

>PA14_16690 | Max Lysine: 3

REQYGPRFLDKIELIRKGAK

>PA14_16700 | Max Lysine: 3

KVAGKDDVTGEELIQREDDK

>PA14_16790 | Max Lysine: 3

VGPGRPKDPAKREAILEAAK

>PA14_16800 | Max Lysine: 3

FRIGGKVIRRLVEVGERVKK

>PA14_16820 | Max Lysine: 3

KVELIGLQDEKIWIDLSNTK

>PA14_16910 | Max Lysine: 3

ALLEALAEARKVEAKSANPK

>PA14_17050 | Max Lysine: 3

YIVNEQKAIPAPLNYKGFPK

>PA14_17080 | Max Lysine: 3

ADVVLKATKVDGVYTADPFK

>PA14_17110 | Max Lysine: 3

MDGNNRWAKKRLLPGVAGHK

>PA14_17140 | Max Lysine: 3

MSLLTLDSIKKMLLGELSVK

>PA14_17190 | Max Lysine: 3

KMLDVKPADGTLYYFVGSDK

>PA14_17210 | Max Lysine: 3

GEGTVIGPHVVLKGPTKIGK

>PA14_17230 | Max Lysine: 3

DSKKLSEARREALFEEIREK

>PA14_17290 | Max Lysine: 3

VPYIATAGETKTKPTQHSVK

>PA14_17330 | Max Lysine: 3

LKKGTETVEERARHELGMVK

>PA14_17400 | Max Lysine: 3

HVIPLYTAECGKCKFCLSGK

>PA14_17420 | Max Lysine: 3

AEKGWKVGNVDATIVAQAPK

>PA14_17480 | Max Lysine: 3

QPSPRATPKATTSFSSKQHK

>PA14_17490 | Max Lysine: 3

ITEKKDALPDAEEWDGVAGK

>PA14_17570 | Max Lysine: 3

EKDAEWENRWRNRIGKPSIK

>PA14_17590 | Max Lysine: 3

MLDKQHLLKIARTPMPFGKY

>PA14_17610 | Max Lysine: 3

LKDLNSRQRRALNKLWTEIK

>PA14_17640 | Max Lysine: 3

LKMAGVAKSEIDARVEQALK

>PA14_17700 | Max Lysine: 3

VYTGEQRKTKSEGRVAGFNK

>PA14_17760 | Max Lysine: 3

VGVKSTGVFCRLTCPARKPK

>PA14_17810 | Max Lysine: 3

PKVREDGTRNAYRIVRLKDK

>PA14_17820 | Max Lysine: 3

GPPFEGDTASYFKGVNRNKK

>PA14_17850 | Max Lysine: 3

DQKEGMRAFLEKRTAEYLGK

>PA14_17890 | Max Lysine: 3

ATAKLRLSRSELKVGGLVPK

>PA14_17900 | Max Lysine: 3

HQFKELEERLGMQLFVRKTK

>PA14_17930 | Max Lysine: 3

KREKLPASRGLRFTGSSPLK

>PA14_17960 | Max Lysine: 3

MTDKHNKKYVVALDQGTTSS

>PA14_18120 | Max Lysine: 3

ALLKEHHDELAKIVSSELGK

>PA14_18160 | Max Lysine: 3

GRLALIDDPASLDIGKLKQK

>PA14_18275 | Max Lysine: 3

DTARFAGKKIYRCGTGVALK

>PA14_18300 | Max Lysine: 3

QKHKLFERIRVFYDGDLRGK

>PA14_18430 | Max Lysine: 3

LNGKLAHAFEAHYDKEFPIK

>PA14_18450 | Max Lysine: 3

HTVDKFAEGCTRFMQGFVKK

>PA14_18470 | Max Lysine: 3

TSKYEGSDSARATLNVKAEK

>PA14_18510 | Max Lysine: 3

QPGEKDIGQELDLVVTKYFK

>PA14_18520 | Max Lysine: 3

MQGNAEKQKALLEQLRAAYK

>PA14_18565 | Max Lysine: 3

IDGDTVLDHGVVKKTVPWFK

>PA14_18640 | Max Lysine: 3

ASNGSIMTVPDVEKRLKALK

>PA14_18650 | Max Lysine: 3

LAEMFEKGELQTLVKDAAAK

>PA14_18700 | Max Lysine: 3

AALTEIFRGIRKALKANGCK

>PA14_18720 | Max Lysine: 3

EAPKAEDKPAPPAAEPAAPK

>PA14_18860 | Max Lysine: 3

MTKANEIETAKVKQFSARIM

>PA14_18900 | Max Lysine: 3

HGFDGKSLGDPADAGWAVKK

>PA14_18930 | Max Lysine: 3

GYPGCKPYAEAIAAGDKINK

>PA14_18985 | Max Lysine: 3

SEFKAEEHHTVHGERKVELK

>PA14_19020 | Max Lysine: 3

SIAFGDQLKLLNKDPATVTK

>PA14_19050 | Max Lysine: 3

FDPEKQMFLADRFIKGTCPK

>PA14_19065 | Max Lysine: 3

VMGNVKNIVAVASGKGGVGK

>PA14_19090 | Max Lysine: 3

FKVFTNIHSAVVDPKNFDEK

>PA14_19170 | Max Lysine: 3

HAPRRGKKVRVSDLNSSYWK

>PA14_19190 | Max Lysine: 3

GTAAVAVGTLLAACKAKGEK

>PA14_19310 | Max Lysine: 3

LQSVSPKHDLPLKPEGQAAK

>PA14_19350 | Max Lysine: 3

RHLDDKAGVAALLAALKAVK

>PA14_19560 | Max Lysine: 3

VLEGETVDYAGKHIQVKGAK

>PA14_19590 | Max Lysine: 3

MTIKAINVRNQFKGTVKEII

>PA14_19620 | Max Lysine: 3

VKDLRLRTFIGIKEEEILNK

>PA14_19630 | Max Lysine: 3

FIGKAHVAYIPTGKVLGLSK

>PA14_19700 | Max Lysine: 3

LMFHEITASSLVKVDASGKK

>PA14_19830 | Max Lysine: 3

ASAGKVDGKGFLSDPFADEK

>PA14_19950 | Max Lysine: 3

KAVPSLKEEQVEELGIFMAK

>PA14_20080 | Max Lysine: 3

SSKAKGRVQVLHVRRGDEVK

>PA14_20110 | Max Lysine: 3

MKNYLQILRETLKAMLSKPM

>PA14_20140 | Max Lysine: 3

DFITKVLPEHEYFGDQVKEK

>PA14_20300 | Max Lysine: 3

IRVDSLNKTFARKQALFNLK

>PA14_20380 | Max Lysine: 3

VAVKGGEKAIDNAHRLLARK

>PA14_20430 | Max Lysine: 3

DALSIGDVNPKGKRMQQLPK

>PA14_20510 | Max Lysine: 3

AMEFDQNGVLIKLTTKEGRK

>PA14_20550 | Max Lysine: 3

GPRGGVYCLTPGGKKSYRRK

>PA14_20590 | Max Lysine: 3

LPVLVSARRISEEMAKLKQK

>PA14_20600 | Max Lysine: 3

YIEPSRFYPVPKPNDKVSAK

>PA14_20650 | Max Lysine: 3

LGFQASVHMSADARQWKKDK

>PA14_20750 | Max Lysine: 3

INVFKVKEVLQCPRLTVMPK

>PA14_20760 | Max Lysine: 3

NKQYLVSSRLNKLMEQQGIK

>PA14_20780 | Max Lysine: 3

MTAKGYPMADILREMNAKLK

>PA14_20880 | Max Lysine: 3

GVQVWERKADGGFPEAKALK

>PA14_21050 | Max Lysine: 3

AKIQRFYKRRRLSPDTVGEK

>PA14_21120 | Max Lysine: 3

HIYGMTPPSGSSQKDKTLFK

>PA14_21180 | Max Lysine: 3

YEPVKWHVRTLLKDLDTAVK

>PA14_21210 | Max Lysine: 3

KLTHFINPTNYIYAVGKYAK

>PA14_21220 | Max Lysine: 3

PLREAALKAADDVLAKAREK

>PA14_21230 | Max Lysine: 3

RFDPRVEWLIDTLKMLKQFK

>PA14_21280 | Max Lysine: 3

PIHWKPFLAPSTLALKFAIK

>PA14_21320 | Max Lysine: 3

LGKSALIDFLSFGSFNKAFK

>PA14_21450 | Max Lysine: 3

SEFKAEEHHTVHGERKVELK

>PA14_21690 | Max Lysine: 3

SLAKELRLDAEQRLKAGQLK

>PA14_21700 | Max Lysine: 3

WDQGPGIPPDKQKVIFEEFK

>PA14_21750 | Max Lysine: 3

ALGGQFGEKYNKDALMRALK

>PA14_21760 | Max Lysine: 3

GFKSLQEGQKVSFVVVNGQK

>PA14_21830 | Max Lysine: 3

RVPALLIAVARKRSRLKLVK

>PA14_21890 | Max Lysine: 3

KAQLIAELGLKVWPLFSAGK

>PA14_22080 | Max Lysine: 3

VEHVAALMKQTTIKVATMPK

>PA14_22100 | Max Lysine: 3

KGTGNEVEVRLGKDGSLTLK

>PA14_22140 | Max Lysine: 3

TIRTNKGRQPAKRTERPSIK

>PA14_22190 | Max Lysine: 3

EQKAAKITEIRLEVEEESRK

>PA14_22210 | Max Lysine: 3

QLLDRKVSEVRNKLEVSERK

>PA14_22240 | Max Lysine: 3

HVATIKATPGLGKRMVEQIK

>PA14_22250 | Max Lysine: 3

VLDSKPASRRASFYKGVDVK

>PA14_22310 | Max Lysine: 3

LKLRPEREFALPDNFWPVKK

>PA14_22320 | Max Lysine: 3

PLCNSVGFYFHEKIWKRFEK

>PA14_22370 | Max Lysine: 3

MSRHDRDMLRYGLHVVKKFK

>PA14_22410 | Max Lysine: 3

RDFQDFLEALSSRKRKQLRK

>PA14_22440 | Max Lysine: 3

YGKGSGVIQGLDLKVRPGEK

>PA14_22450 | Max Lysine: 3

VQTGGFSAGMQEKKTRAPIK

>PA14_22460 | Max Lysine: 3

KAEQAGLREKIEARWAENDK

>PA14_22470 | Max Lysine: 3

YTNKEKPLGPASQILMEMLK

>PA14_22520 | Max Lysine: 3

LEIDMSKKILVRGATGTVGK

>PA14_22590 | Max Lysine: 3

RRRWGYGYWSLSAYLKHKVK

>PA14_22650 | Max Lysine: 3

DKRVVASRRFEVSQPVDGKK

>PA14_22660 | Max Lysine: 3

SGIKVGDVAFLRLDPKDPRK

>PA14_22680 | Max Lysine: 3

ALLKTVGHALHDFCEPEKPK

>PA14_22760 | Max Lysine: 3

RKLTLYDRSLDMHVSNLRKK

>PA14_22780 | Max Lysine: 3

ADPYRAAGVYAEVVVKPFKK

>PA14_22880 | Max Lysine: 3

MRSAPKKGERLKSPCINICE

>PA14_22890 | Max Lysine: 3

KAAAHLQAGAGKVLISAPGK

>PA14_22910 | Max Lysine: 3

PTGISNKEKAAVRQLFAEGK

>PA14_22960 | Max Lysine: 3

PGKPLALKRCIGNLLDNALK

>PA14_23010 | Max Lysine: 3

DAKLRVEMRTELKLMHQRLK

>PA14_23030 | Max Lysine: 3

MYKNKKTRPAARTVGCLFAL

>PA14_23060 | Max Lysine: 3

KNLLEQIQSRLDELNKAERK

>PA14_23070 | Max Lysine: 3

YLRTGKRMPQKLSQIVIHFK

>PA14_23100 | Max Lysine: 3

GAKLALFYGDHLVVYKRDEK

>PA14_23130 | Max Lysine: 3

MAPPKQSNTIDFDAAKQKRG

>PA14_23220 | Max Lysine: 3

GLEVKDIIGLTYNPLTKHYK

>PA14_23270 | Max Lysine: 3

KKAIEEARRYGTVNVAASAK

>PA14_23310 | Max Lysine: 3

ADLAKLDLGKAVLTDVGSAK

>PA14_23360 | Max Lysine: 3

SLMYMRGTKALEAEIKALEK

>PA14_23370 | Max Lysine: 3

MRKKTLCVFGTRPEAIKMAP

>PA14_23380 | Max Lysine: 3

KQVIGVDVNSKAVETINQGK

>PA14_23390 | Max Lysine: 3

KIIKSTQALARPLNQLFLPK

>PA14_23450 | Max Lysine: 3

LARQAAKAGVKRFVFISSIK

>PA14_23500 | Max Lysine: 3

TGVDLELDDWKQVLDVLKAK

>PA14_23520 | Max Lysine: 3

MIAGLLYGLEKKAPHWELLK

>PA14_23760 | Max Lysine: 3

DRANVDTDQIIPKQFLKSIK

>PA14_23800 | Max Lysine: 3

PEVGKDIAPLKDAYSIDELK

>PA14_23850 | Max Lysine: 3

QVKPYAVDVSGGVEASKGIK

>PA14_24180 | Max Lysine: 3

QKKPQMAEAVLFFNDSGVCK

>PA14_24220 | Max Lysine: 3

QNHFTCAPGDTVTISKKPQK

>PA14_24260 | Max Lysine: 3

LRLEYLGSIFCTHCGRKTKK

>PA14_24270 | Max Lysine: 3

GLYKSGKMFCTQCEAEGFRK

>PA14_24430 | Max Lysine: 3

KSALQGLKGYLSSQILGQEK

>PA14_24480 | Max Lysine: 3

NDGLLQRLGIRRLSQKLKVK

>PA14_24580 | Max Lysine: 3

LQEPPEPRRQRKRKTPASGK

>PA14_24600 | Max Lysine: 3

LVTASTETDKSAAGLKRSAK

>PA14_24610 | Max Lysine: 3

YLDVAPKKANGRTILLMHGK

>PA14_24665 | Max Lysine: 3

EFSGKGSGIDNTHFGALKVK

>PA14_24690 | Max Lysine: 3

QLKNGVLNGNLYLKGGGDPK

>PA14_24700 | Max Lysine: 3

KKLCVDEFQELGFELNFHYK

>PA14_24710 | Max Lysine: 3

SNKQISEQMFISNKTVSTYK

>PA14_24770 | Max Lysine: 3

IRGKIHDSLKRARDTLRDTK

>PA14_24840 | Max Lysine: 3

RILAESGVAKMTLYKHFRSK

>PA14_24860 | Max Lysine: 3

FATIMKRMTANKPSIEQAHK

>PA14_24880 | Max Lysine: 3

FEQLDGKRARVNKDQVRTVK

>PA14_24910 | Max Lysine: 3

MALREAGELTLWKLAIKPGK

>PA14_24920 | Max Lysine: 3

LPLRVKNALRQLLKDGLPRK

>PA14_24940 | Max Lysine: 3

AKALLKRFGGVFTGTLLGRK

>PA14_24960 | Max Lysine: 3

DLQGNLLGKGKVELEAYYSK

>PA14_24970 | Max Lysine: 3

MDKDKVLLVLHGKQAGNEEV

>PA14_25000 | Max Lysine: 3

FGLWQGEGQLTEEKVWKRLK

>PA14_25160 | Max Lysine: 3

ELGFKSPNAAEEHLKALARK

>PA14_25180 | Max Lysine: 3

ITSKAGVNLAAVNYHFGSKK

>PA14_25270 | Max Lysine: 3

LSHLKFRAAMKRQGVDTFFK

>PA14_25280 | Max Lysine: 3

MAVQVGDRVKLGQALFTDKK

>PA14_25305 | Max Lysine: 3

LRNLLDKVEHHFEKGGRYEK

>PA14_25320 | Max Lysine: 3

EPGMSGKEVKALFDSRITAK

>PA14_25350 | Max Lysine: 3

TPVKQDMQIRVPEEVFGVKK

>PA14_25420 | Max Lysine: 3

KVMNKRIDLLGQVMVQSLLK

>PA14_25430 | Max Lysine: 3

EAKVSIIPDFFKQGSLAELK

>PA14_25450 | Max Lysine: 3

VRLELKDLYQSPQVAAKVVK

>PA14_25520 | Max Lysine: 3

CPLTKGPLVLSEDKTELISK

>PA14_25550 | Max Lysine: 3

QADGRLKLAAGWLIDKGGWK

>PA14_25640 | Max Lysine: 3

GTEEIKGNQQVKLAASLLQK

>PA14_25670 | Max Lysine: 3

MSTIEERVKKIVAEQLGVKE

>PA14_25690 | Max Lysine: 3

GGSVKGFNVEEYLSAKEARK

>PA14_25760 | Max Lysine: 3

GQGVREQRAQVVEGVKKLLK

>PA14_25770 | Max Lysine: 3

LMEEPEKIPVAGKVVWITPK

>PA14_25810 | Max Lysine: 3

NGCSWNKCGFCEMYTQPQKK

>PA14_25830 | Max Lysine: 3

MSDTGKANDLLAQIPRGEKK

>PA14_25880 | Max Lysine: 3

GAIQHLAGMKDSKVIVAINK

>PA14_25960 | Max Lysine: 3

MKTLAKLPVTIVTGFLGAGK

>PA14_25970 | Max Lysine: 3

VRARAVNPKWIAGMKRHGYK

>PA14_26420 | Max Lysine: 3

MNDFGTPLVGGKYHKRLREK

>PA14_26510 | Max Lysine: 3

KALRLLRAAPVVGYFVAKGK

>PA14_26560 | Max Lysine: 3

DHKLSEQSLAKAQSEFDQVK

>PA14_26570 | Max Lysine: 3

QRTMRSVSTISTHKKNAMVK

>PA14_26590 | Max Lysine: 3

TRPKGGFILWVSLPAKVNTK

>PA14_26670 | Max Lysine: 3

VGKGQLLLVLEAMKMEHPLK

>PA14_26730 | Max Lysine: 3

LAALGAHVVLVGRKAEKLEK

>PA14_26780 | Max Lysine: 3

GLLLLRDSAKKLPLSEEQLK

>PA14_26810 | Max Lysine: 3

LRKALVDLKESQAQLVQSEK

>PA14_26830 | Max Lysine: 3

QIFRYLPKPIRRGLFEKGLK

>PA14_26890 | Max Lysine: 3

AALALADQLDPKLCRVKVGK

>PA14_27050 | Max Lysine: 3

LNSRSPSARNLTLKLIPKPK

>PA14_27100 | Max Lysine: 3

MKKKSLLPLGLAIGLASLAA

>PA14_27130 | Max Lysine: 3

YIYGKKMLREIDSRVRFLRK

>PA14_27180 | Max Lysine: 3

GPYKLTLGVPGYLIHGSNKK

>PA14_27220 | Max Lysine: 3

KFVAGQRKQTLPADASITGK

>PA14_27250 | Max Lysine: 3

MAGWETHKKAQWLCQSRKGM

>PA14_27500 | Max Lysine: 3

VSKSNKLANVCYDIRGPVLK

>PA14_27680 | Max Lysine: 3

DKIDQSIAKYSETLASRGGK

>PA14_27730 | Max Lysine: 3

AFIKQHGFFGLIIPKEYGGK

>PA14_27770 | Max Lysine: 3

AKIAKERAERYLTQLGLWDK

>PA14_27840 | Max Lysine: 3

YFKPRAIEVKAGETVRFVLK

>PA14_27920 | Max Lysine: 3

NTVDTRANLLKSEKLISGDK

>PA14_27940 | Max Lysine: 3

KPSEVLAHINRGLINTKLGK

>PA14_27980 | Max Lysine: 3

PKENREVPPLRYEVAAQLKK

>PA14_28010 | Max Lysine: 3

LLSAHQYRCDKREVKVVNAK

>PA14_28030 | Max Lysine: 3

PPGKERNSFPRFPQRSWMKK

>PA14_28080 | Max Lysine: 3

AQAIGITEQNLSLLKQGKVK

>PA14_28130 | Max Lysine: 3

EDELAELILKVSSLTPEFKK

>PA14_28140 | Max Lysine: 3

HNLDRQQLLKMLDDQGVQKK

>PA14_28170 | Max Lysine: 3

DKQTSTPGPRQPAKGGAASK

>PA14_28210 | Max Lysine: 3

IGLGMRLPRFGKVNLNVKGK

>PA14_28250 | Max Lysine: 3

KAFGEKYVIFPNPTDGHWMK

>PA14_28260 | Max Lysine: 3

IYSEEQMRKALGLAETRPKK

>PA14_28320 | Max Lysine: 3

MRYKPEQKQATRALLLSKAA

>PA14_28350 | Max Lysine: 3

KAPLASTFGDAIGSPAWKSK

>PA14_28400 | Max Lysine: 3

KQGDSGSAADDLSKGGAAVK

>PA14_28450 | Max Lysine: 3

AALSSTAMAAKLDEKVPYPK

>PA14_28540 | Max Lysine: 3

MSVDLKKIAAAFPEFQLSSK

>PA14_28570 | Max Lysine: 3

SAGQANAPRTFSEAKKIGWK

>PA14_28690 | Max Lysine: 3

LEQIRVHYLGKKGELTQVMK

>PA14_28720 | Max Lysine: 3

KAEIAERLYEELGLNKREAK

>PA14_28730 | Max Lysine: 3

KQLIRQMIAELEEVLLVLKK

>PA14_28830 | Max Lysine: 3

QGVVERFISQAKTEAKASGK

>PA14_28840 | Max Lysine: 3

WPHHGSLAKDIREETEKALK

>PA14_28850 | Max Lysine: 3

LRRWVKQLQQERDSVTPKSK

>PA14_28895 | Max Lysine: 3

WLEPQKQQYVSQILDFFEKK

>PA14_29030 | Max Lysine: 3

RNKPLASLANMAAVKFQLRK

>PA14_29040 | Max Lysine: 3

ITRKKPAPDDHDAWFGLPGK

>PA14_29200 | Max Lysine: 3

IPDGTDIPPDLKVKQDSYNK

>PA14_29240 | Max Lysine: 3

QSIDRQTLKNNVVGLAKAAK

>PA14_29270 | Max Lysine: 3

GIALESPKITRKLTTAWFAK

>PA14_29320 | Max Lysine: 3

FTEGWDRQVRFVREEGKKIK

>PA14_29330 | Max Lysine: 3

LELDENENKQSRKLLQELAK

>PA14_29390 | Max Lysine: 3

RSSKGGTPANFNEIRMEDKK

>PA14_29710 | Max Lysine: 3

DEALKLRDAGTIKSFEELNK

>PA14_29740 | Max Lysine: 3

KDTWRSRSLWDSELEVPHKK

>PA14_29770 | Max Lysine: 3

KVLVLLGRHDEQGKGAGAFK

>PA14_29800 | Max Lysine: 3

LVLSGGAVLILKKQADTEVK

>PA14_29820 | Max Lysine: 3

RRIKQAVPLSQEHKHHLFAK

>PA14_29860 | Max Lysine: 3

ALWGHSSDDGKKTRIYAATK

>PA14_29880 | Max Lysine: 3

VIIACHHEQNIFKMGGLWKK

>PA14_29920 | Max Lysine: 3

FKRQDLVYEKHDLLISGPGK

>PA14_29930 | Max Lysine: 3

GADMVKMFFKEDWTPPFADK

>PA14_29970 | Max Lysine: 3

TVKDSGLKGRGGAGFPTGVK

>PA14_29990 | Max Lysine: 3

AKQDLEQEALRFKPEDWGMK

>PA14_30020 | Max Lysine: 3

RIGALDWAPESRRKRQAKLK

>PA14_30110 | Max Lysine: 3

IKEIERTTNHDVKAVEYLLK

>PA14_30200 | Max Lysine: 3

MLSGKVKWFNNAKGYGFILA

>PA14_30210 | Max Lysine: 3

FNMDREKATKIMLTVHTQGK

>PA14_30240 | Max Lysine: 3

TAHISGKMRKNYIRILTGDK

>PA14_30260 | Max Lysine: 3

GLQAVYLGYWIKNCRKMNYK

>PA14_30360 | Max Lysine: 3

DWPSYAGKKRHLPWYLLAAK

>PA14_30470 | Max Lysine: 3

ALLVPKDSPVRSIADLKGRK

>PA14_30490 | Max Lysine: 3

GEAGFPKGKPQSVGAQRLLK

>PA14_30520 | Max Lysine: 3

VALVVGKDSPIRSVAGLKGK

>PA14_30560 | Max Lysine: 3

ARHPLPDKRYHVACRKSGCK

>PA14_30570 | Max Lysine: 3

KVRKLLGPDAPVDSWDLVFK

>PA14_30580 | Max Lysine: 3

VIALMKKRIHFEDAVREEAK

>PA14_30650 | Max Lysine: 3

EDCLKLARELKPDVVLMDVK

>PA14_30690 | Max Lysine: 3

MKKPAPAHIKLLFCIGISTA

>PA14_30700 | Max Lysine: 3

ATVKGYLPAERIKAAYPHSK

>PA14_30730 | Max Lysine: 3

PNKSVTDLSSYSHRVIFKNK

>PA14_30750 | Max Lysine: 3

WMKLMLHELRAAREHVKSGK

>PA14_30770 | Max Lysine: 3

DRINGYAETNTAIVKKSPVK

>PA14_30810 | Max Lysine: 3

AYKYNFLNAWRLEAVRLRKK

>PA14_30840 | Max Lysine: 3

ITQEVGQAKEALEKEIGERK

>PA14_30850 | Max Lysine: 3

VSKSEGLDGLPADYSKLPQK

>PA14_30860 | Max Lysine: 3

LTSTEKTWMASVSWEYPKDK

>PA14_30880 | Max Lysine: 3

NKTSFLAEKVLGNVVSAGVK

>PA14_30910 | Max Lysine: 3

KSEAERELGRLRRQWFAKRK

>PA14_30950 | Max Lysine: 3

IQPEHAQRLDELAAKKGVSK

>PA14_30960 | Max Lysine: 3

SSVAPIKARKLRYYADANFK

>PA14_31000 | Max Lysine: 3

KALDNLGAFKRWFGFQPNTK

>PA14_31010 | Max Lysine: 3

FVPAAVALFVTGKVSEKETK

>PA14_31110 | Max Lysine: 3

DSQDLMAYPFFSLAKSKRVK

>PA14_31180 | Max Lysine: 3

ADKDGFTGQLRTLTLNVKAK

>PA14_31190 | Max Lysine: 3

HYEVVAGDRRLTALKLLAKK

>PA14_31440 | Max Lysine: 3

ATKLGPGGEKEASIELLSLK

>PA14_31480 | Max Lysine: 3

ELALELGFADGSAFHKAFKK

>PA14_31530 | Max Lysine: 3

GKTERLVDSDEQPPKARLDK

>PA14_31580 | Max Lysine: 3

AEGDRWQPTAIMEELKAKAK

>PA14_31610 | Max Lysine: 3

GGILLSLLKTRTPKATVEDK

>PA14_31650 | Max Lysine: 3

EDCDIGIGEENRKRWLKTGK

>PA14_31690 | Max Lysine: 3

KPWKLAVQVGGELQQALTLK

>PA14_31870 | Max Lysine: 3

KPRVNGELVKVLFQEGQEVK

>PA14_31890 | Max Lysine: 3

KKNAIMMIDFALDAERNEGK

>PA14_31900 | Max Lysine: 3

TAIAAANSNGPKGAVEKDDK

>PA14_31990 | Max Lysine: 3

LKLMAGKQGYDFAFTREEGK

>PA14_32080 | Max Lysine: 3

YTCPFHGWTFNNSGKLLKVK

>PA14_32220 | Max Lysine: 3

KLKIGAGEVDADVAHAIAIK

>PA14_32230 | Max Lysine: 3

VKMTVKLPVDMDPQQAERLK

>PA14_32270 | Max Lysine: 3

NYPLDDKLSLLGGANYYKVK

>PA14_32390 | Max Lysine: 3

AVLLKGHHEPKDRFSVFLDK

>PA14_32530 | Max Lysine: 3

LSKQLHDLKQGGSREHPVMK

>PA14_32540 | Max Lysine: 3

VEKTRQKFHANDGVNLYGQK

>PA14_32740 | Max Lysine: 3

VSVTDKNRSTGSKADDDWEK

>PA14_32750 | Max Lysine: 3

LTLPRGRPFWSKWATAWKIK

>PA14_32770 | Max Lysine: 3

GSSVKDAAKAAVSSAIETGK

>PA14_32810 | Max Lysine: 3

VLTAVKGGAQITAKLPTAAK

>PA14_32820 | Max Lysine: 3

VLTAVKGGAQITAKLPTAAK

>PA14_32890 | Max Lysine: 3

LEQLDGKRTRVNKDQVRSIK

>PA14_32950 | Max Lysine: 3

EVPETQLGETKVLKTWFAGK

>PA14_33010 | Max Lysine: 3

KYAEGYPGKRYYGGCEHVDK

>PA14_33050 | Max Lysine: 3

LWCAQQTGKTRAAVFHRAKK

>PA14_33160 | Max Lysine: 3

RGKTSVTGARVTEVKELQAK

>PA14_33240 | Max Lysine: 3

HKVVILFEGRDAAGKGGVIK

>PA14_33280 | Max Lysine: 3

APKVVALLNPGALPKTSSGK

>PA14_33330 | Max Lysine: 3

FIQERLQGKLDKLQPDEDDK

>PA14_33340 | Max Lysine: 3

FHDIGKASQAFQAKLRNRGK

>PA14_33350 | Max Lysine: 3

KRLFKLAAQATRYGEFVRAK

>PA14_33420 | Max Lysine: 3

EINAWDNPEFVAAVKATGKK

>PA14_33430 | Max Lysine: 3

LAVWDVQLKAGKHLALDLPK

>PA14_33530 | Max Lysine: 3

ATLKKQLLEATASNETRLAK

>PA14_33610 | Max Lysine: 3

ADLGESLKAIKEQLRGVPDK

>PA14_33630 | Max Lysine: 3

LKAESAPVEDALAKSLEALK

>PA14_33650 | Max Lysine: 3

IEKVGSLSPQERKALAVLLK

>PA14_33690 | Max Lysine: 3

GTSYVGQHIIAKLRKELGEK

>PA14_33700 | Max Lysine: 3

MTKRKLAYIWSLRNAAADKA

>PA14_33720 | Max Lysine: 3

SGVKLPIREIGKLVRELNQK

>PA14_33760 | Max Lysine: 3

ERDEDAATTVAVIGYKVRKK

>PA14_33810 | Max Lysine: 3

HSQYLEHMAKQPCSSGKPMK

>PA14_33820 | Max Lysine: 3

KSVAIEVRGADGKLSRVEHK

>PA14_33860 | Max Lysine: 3

LNWGDVEWIKRRWGGKLVLK

>PA14_33890 | Max Lysine: 3

TYNRIKTAIHNASAKLQEAK

>PA14_34050 | Max Lysine: 3

RLKLRLLNVGKNELQNDLEK

>PA14_34070 | Max Lysine: 3

AIEKKELPLVVGILADLSGK

>PA14_34250 | Max Lysine: 3

TFKESGETDKAAFYARQEVK

>PA14_34280 | Max Lysine: 3

GLQLLQKAGLLKLRDGVGYK

>PA14_34320 | Max Lysine: 3

AEAKVLSTEISLAASEKLFK

>PA14_34330 | Max Lysine: 3

GKALFTQPAMPIKCAGAPQK

>PA14_34370 | Max Lysine: 3

MADLKIRNLQKGFDGQAIIK

>PA14_34420 | Max Lysine: 3

PEFSGGEWKKALDFYVSTLK

>PA14_34540 | Max Lysine: 3

ELQKRGVYKTEYAPGTLREK

>PA14_34680 | Max Lysine: 3

PFVERIPGDLKAWNRKLMLK

>PA14_34730 | Max Lysine: 3

ESGRSVPSIKVLCKIAQGLK

>PA14_34770 | Max Lysine: 3

FLQYAAAKQWGDKQFDRLDK

>PA14_34810 | Max Lysine: 3

SAFVVLAELPKTLNGKIDRK

>PA14_34870 | Max Lysine: 3

GKHFIVSMAPEFPYLHKNGK

>PA14_34960 | Max Lysine: 3

PNSKWMLGDWGGKRTELLEK

>PA14_34970 | Max Lysine: 3

TLLDLKTADGVKPALIAPTK

>PA14_34990 | Max Lysine: 3

LSATYDSDFEAGGKTIGKGK

>PA14_35010 | Max Lysine: 3

VAGGKRVKLALCGAALNGQK

>PA14_35060 | Max Lysine: 3

QVEGLDHDWDRLIHKVKVEK

>PA14_35140 | Max Lysine: 3

FKAMTFTSPLQYLKSVRLYK

>PA14_35190 | Max Lysine: 3

VAINPRGSLVNSIKVLKTPK

>PA14_35250 | Max Lysine: 3

TVFRAIRRLEKNLGKALFDK

>PA14_35290 | Max Lysine: 3

EKVFGTSGTAHTVKGQVVGK

>PA14_35320 | Max Lysine: 3

MKKVVLYKRLSAPLMERLRE

>PA14_35330 | Max Lysine: 3

GYFFFQVPGAIYAEKRSVKK

>PA14_35390 | Max Lysine: 3

YPKLAGLDREYLYKQIYDLK

>PA14_35490 | Max Lysine: 3

EWKDAIVDRLTSGVAALLKK

>PA14_35500 | Max Lysine: 3

QAVPVIGLRRKIAQKMQDAK

>PA14_35520 | Max Lysine: 3

WSKHPASQVPDGYYKVPLDK

>PA14_35530 | Max Lysine: 3

AMLKTRLFDARMLTAQRQKK

>PA14_35600 | Max Lysine: 3

VIKRVALLRALYFPFPVRKK

>PA14_35670 | Max Lysine: 3

ANPKPVYLALQRFLKVTGPK

>PA14_35760 | Max Lysine: 3

IKLISASIDDELLLVELMKK

>PA14_35770 | Max Lysine: 3

LLVPKWRHSIAKDVSEMPPK

>PA14_35780 | Max Lysine: 3

NVKSGIGLKSYEWSSLELLK

>PA14_35820 | Max Lysine: 3

TANGWDIKALMTYVGWKDIK

>PA14_35850 | Max Lysine: 3

VSTDKAARLKGFVSSWSNLK

>PA14_35890 | Max Lysine: 3

GADAVEAALKLARKATGRTK

>PA14_35940 | Max Lysine: 3

HFKAPRHVTLTELPKTATGK

>PA14_36000 | Max Lysine: 3

PQPRSRKEPSRTKDLHAIGK

>PA14_36090 | Max Lysine: 3

DDYARLGVAPKLKLSNTELK

>PA14_36130 | Max Lysine: 3

VLNKGGGNGAEAFLDLKLAK

>PA14_36170 | Max Lysine: 3

FKKLGYPIAPLVLAAVLGDK

>PA14_36190 | Max Lysine: 3

NRDNEVKVAAQKLIGQPAGK

>PA14_36250 | Max Lysine: 3

PYLQSQYREFFQLSPKAQKK

>PA14_36270 | Max Lysine: 3

IEQVKALLEIMGQKTWFFGK

>PA14_36290 | Max Lysine: 3

GQIGKLKGCRVVGVAGGAEK

>PA14_36300 | Max Lysine: 3

EILQTAGVPKGSFYHYFKSK

>PA14_36345 | Max Lysine: 3

IVTTLIEEGFPTKGFSVKGK

>PA14_36400 | Max Lysine: 3

VRDHPSRALNRKTRGAPKDK

>PA14_36420 | Max Lysine: 3

QPGSSSRRVNTSMKTTRKPK

>PA14_36470 | Max Lysine: 3

ALRKPGRAGQMNLSYLYLKK

>PA14_36530 | Max Lysine: 3

KPKDSTSFAGDLHRRWVDLK

>PA14_36570 | Max Lysine: 3

ITTPEYGCGLHGILKCKVEK

>PA14_36710 | Max Lysine: 3

EVVHGKHSLLDKMPGDRWQK

>PA14_36730 | Max Lysine: 3

HAKKGSRARDYYVWSDSDEK

>PA14_36810 | Max Lysine: 3

KFHWRPKSGTCSLVWDEAQK

>PA14_36870 | Max Lysine: 3

QPKPEFVSADYRPAAKLEGK

>PA14_36910 | Max Lysine: 3

YARKRDFRQTPEPSGRKPRK

>PA14_37090 | Max Lysine: 3

LENGKIKPEAAFEVGMVPGK

>PA14_37130 | Max Lysine: 3

YRIHKSLGHHKHPLGRMFYK

>PA14_37170 | Max Lysine: 3

PKALPGPTPKLFFAPAQAEK

>PA14_37200 | Max Lysine: 3

AQGIKVSASNYDKLPQDTRK

>PA14_37260 | Max Lysine: 3

DDYAKLGLTAKARVSNSLLK

>PA14_37430 | Max Lysine: 3

IKNTLIAQLFQQNYDWLCKK

>PA14_37470 | Max Lysine: 3

LKRRLNEEAAEQGRRLKFWK

>PA14_37490 | Max Lysine: 3

KPAGTLATVVVTGTRKADVK

>PA14_37640 | Max Lysine: 3

TSKIADMQVPEVFQIMLGKK

>PA14_37650 | Max Lysine: 3

SDDSDLTFTKLRWKALQGSK

>PA14_37690 | Max Lysine: 3

LDKASVMVFVDRLTPAKLAK

>PA14_37730 | Max Lysine: 3

KADGSIDHVVTGLANLGKVK

>PA14_37745 | Max Lysine: 3

EGLPHLRKRIADEMANDKAK

>PA14_37900 | Max Lysine: 3

SAIYGSDAIAGVVNIVLKKK

>PA14_37965 | Max Lysine: 3

HEQFGDGIISAINFKLDIKK

>PA14_38010 | Max Lysine: 3

LPAFKSNEALRERFFGGVKK

>PA14_38110 | Max Lysine: 3

FPAAALKVGFIGKVFVSALK

>PA14_38140 | Max Lysine: 3

NGVVRGKRIERNSLPKVFEK

>PA14_38180 | Max Lysine: 3

WFGGIKLSLSSDYKNFFTLK

>PA14_38190 | Max Lysine: 3

SHDQLVRVLELLGAKQFKVK

>PA14_38320 | Max Lysine: 3

RRLGFSKEDEITIVFCGSKK

>PA14_38330 | Max Lysine: 3

ELGKKGLDLQFNSDIARIDK

>PA14_38380 | Max Lysine: 3

MARKTKEESQKTRDGILDAA

>PA14_38395 | Max Lysine: 3

AEASHAAAADKLKRYADLIK

>PA14_38420 | Max Lysine: 3

IDDPRNARKFSLGSANKRNK

>PA14_38430 | Max Lysine: 3

QERIYSPRDKVSLKLILRGK

>PA14_38440 | Max Lysine: 3

TAFIVERDWKGFSRGPKLDK

>PA14_38460 | Max Lysine: 3

IVGNDATVKGGTYYPLTVKK

>PA14_38480 | Max Lysine: 3

RIGYPVLLKAAAGGGGKGMK

>PA14_38490 | Max Lysine: 3

LPKKVRLVEVGPRDGLQNEK

>PA14_38500 | Max Lysine: 3

MEKNSSPAETSGKQKVRSAE

>PA14_38570 | Max Lysine: 3

GNVVEAAKRLGLGRSTLYKK

>PA14_38590 | Max Lysine: 3

ASAGKSAYVAAKHGVLGLTK

>PA14_38630 | Max Lysine: 3

QPRAGTTVEALAKLKPAFRK

>PA14_38710 | Max Lysine: 3

GNKMFAILDFVDKDGGLAFK

>PA14_38740 | Max Lysine: 3

RRALARLGVPLLNKPLKPGK

>PA14_38780 | Max Lysine: 3

LLAGSRKAFAQKLAMARAVK

>PA14_38840 | Max Lysine: 3

KRIAKIAFTGSTPVGSHILK

>PA14_38900 | Max Lysine: 3

EHLGVSAKTVSNHVSLLKQK

>PA14_38930 | Max Lysine: 3

ALTRKQLLVLERMTKGESNK

>PA14_38970 | Max Lysine: 3

EARLREAKREAEQANLSKTK

>PA14_39090 | Max Lysine: 3

TAGASEGKSAYDEFKEYMQK

>PA14_39150 | Max Lysine: 3

AGKTFRYTENGPVGLAGDKK

>PA14_39190 | Max Lysine: 3

RKHVIHAEHVDDMTWKDALK

>PA14_39240 | Max Lysine: 3

QAQGAKDLNHDGGAFAFKEK

>PA14_39350 | Max Lysine: 3

KSLANEFFLTMEDGAKAYQK

>PA14_39390 | Max Lysine: 3

EFKRNRTWHIEGIKPGAIHK

>PA14_39440 | Max Lysine: 3

PFKFNRFARKVLGERYEQDK

>PA14_39460 | Max Lysine: 3

TRVLRDAHAKAHGCVKAEFK

>PA14_39500 | Max Lysine: 3

QGDKGSVSVGDKHFRTQAFK

>PA14_39560 | Max Lysine: 3

IEATYNPVKDGSGRIIKVIK

>PA14_39590 | Max Lysine: 3

GFPRIGRDRELKKAQEAFWK

>PA14_39610 | Max Lysine: 3

TILELKVLIGLALKRFEDAK

>PA14_39620 | Max Lysine: 3

VENPGEADMRMLVKDGKELK

>PA14_39820 | Max Lysine: 3

GATYIKPEQNKTGDPASEGK

>PA14_39860 | Max Lysine: 3

KPEAQRLALRQRIDDMKRRK

>PA14_39960 | Max Lysine: 3

DAVELRRKNRETVVKYMNTK

>PA14_39980 | Max Lysine: 3

QRTVKFHIVNAMRKLNSSNK

>PA14_40010 | Max Lysine: 3

DFKLRQGNSVVTNIHRFKSK

>PA14_40020 | Max Lysine: 3

LFKTRYSKYGYGIDVRRTYK

>PA14_40040 | Max Lysine: 3

EHVPFHKTPLRTLFDLKNNK

>PA14_40080 | Max Lysine: 3

KLPVLAVLGAALLLSACAKK

>PA14_40100 | Max Lysine: 3

HDPVRKNFSFGKCFDFHYLK

>PA14_40120 | Max Lysine: 3

KKRYAGLVRRADGGEEMVFK

>PA14_40180 | Max Lysine: 3

SAVAGKRVTTIEGIQSKPGK

>PA14_40240 | Max Lysine: 3

IDYLQQWLGKRTLVITTHKK

>PA14_40320 | Max Lysine: 3

PTATLAAKAVDLVTNKRSIK

>PA14_40370 | Max Lysine: 3

KKDELSQLFRDHLGRANLRK

>PA14_40540 | Max Lysine: 3

KHFRPDGSPFKRLRFLRGTK

>PA14_40630 | Max Lysine: 3

CIAYCKPGEEKVEDTAIALK

>PA14_40640 | Max Lysine: 3

PIQGVIVHKTHITYPLTAKK

>PA14_40650 | Max Lysine: 3

KDGLRLGQKCSGESVDSCAK

>PA14_40660 | Max Lysine: 3

IVNACKNSWDKSYLAGTPNK

>PA14_40710 | Max Lysine: 3

TVTRRPISVTRKPNFKWSIK

>PA14_40830 | Max Lysine: 3

AKQILDRATVADGVDKPLLK

>PA14_40890 | Max Lysine: 3

QGIYSVTKAAVISMTKVFAK

>PA14_40960 | Max Lysine: 3

QLLPRKSRKQLFSRIDRELK

>PA14_41020 | Max Lysine: 3

MYKDLKFPVLIVHRDIKADT

>PA14_41080 | Max Lysine: 3

SLELSTNPFLRVSENSVKKK

>PA14_41110 | Max Lysine: 3

LVFERVKGWWGEKLPVNRGK

>PA14_41130 | Max Lysine: 3

APKHALTLYDEPPKYPADFK

>PA14_41140 | Max Lysine: 3

TKNSFLDEIGKQYVVTARAK

>PA14_41160 | Max Lysine: 3

TIEKQLAEVLALHKGLGGAK

>PA14_41300 | Max Lysine: 3

MSMKWTEQRLRKALKQMANN

>PA14_41350 | Max Lysine: 3

ADLVVVAAGKPGLVKGEWIK

>PA14_41360 | Max Lysine: 3

IEPGEAKEDPLDFVLWKGAK

>PA14_41380 | Max Lysine: 3

QYEFSRLNLNYTVTSKRKLK

>PA14_41430 | Max Lysine: 3

LDHKACEMKAAATAMQLIGK

>PA14_41590 | Max Lysine: 3

LLGFVIAKLLDALLSKLLAK

>PA14_41630 | Max Lysine: 3

RPLKAVADLLEDLEAGKGPK

>PA14_41680 | Max Lysine: 3

TEEDMERLQLPNALKQYKHK

>PA14_41810 | Max Lysine: 3

SGPDRLKRAEQRLKSVDWNK

>PA14_41830 | Max Lysine: 3

VVGYRLRQKDPKRQSVIAFK

>PA14_41840 | Max Lysine: 3

YRDGHGECCGIRKIEPLKRK

>PA14_42120 | Max Lysine: 3

GAGKPSAGPFYLVWSKPEEK

>PA14_42160 | Max Lysine: 3

CRKPKELAHVLAHLPELVVK

>PA14_42230 | Max Lysine: 3

QWGFKGLTISDHGAVKELIK

>PA14_42310 | Max Lysine: 3

ANALKEQANAANKDVNDAIK

>PA14_42390 | Max Lysine: 3

VKGLKELLVHEHPPMLACLK

>PA14_42570 | Max Lysine: 3

EWLAKYEEVELLLKIGEYQK

>PA14_42760 | Max Lysine: 3

GKTTGTPIGLLIRNTDQKSK

>PA14_42780 | Max Lysine: 3

KARYSAGRVSELHSDWHKDK

>PA14_42840 | Max Lysine: 3

FKSEMRGVKRISVDRADTGK

>PA14_42850 | Max Lysine: 3

EDVNREGLLDTPKRAAKAMK

>PA14_42860 | Max Lysine: 3

SMSELVKDKSEGGWLSRSMK

>PA14_42910 | Max Lysine: 3

TFGGEKFFQLLDRLSKNPVK

>PA14_42940 | Max Lysine: 3

YVVPVTAKELTRVELKLGRK

>PA14_42980 | Max Lysine: 3

GASVKGEFERRLKGVIDEVK

>PA14_43000 | Max Lysine: 3

PRYYDQHAAKVSKRLLGGLK

>PA14_43040 | Max Lysine: 3

MAKEGSVAPKERINVTFKPA

>PA14_43070 | Max Lysine: 3

GQPTGQRVHKPVVITKVFDK

>PA14_43080 | Max Lysine: 3

KVVLEAGAELTLKGGGSFLK

>PA14_43140 | Max Lysine: 3

DDDKRKRLLGLTAEGVRLEK

>PA14_43190 | Max Lysine: 3

GAIDYKNEDLAAGLKRECPK

>PA14_43280 | Max Lysine: 3

KRFMKRNDTAEAGCRLYLTK

>PA14_43290 | Max Lysine: 3

WKELDLAKAEGFAGTVSYSK

>PA14_43320 | Max Lysine: 3

ADKGFCKHSGEQVLNERQTK

>PA14_43380 | Max Lysine: 3

RGKARADSLKAGSQGLSANK

>PA14_43400 | Max Lysine: 3

LAGLMVGRTPEYLGKKLEAK

>PA14_43420 | Max Lysine: 3

VERGTPGLSLGKPDRKMGQK

>PA14_43430 | Max Lysine: 3

TPRESLLGNQELAKKTGLPK

>PA14_43440 | Max Lysine: 3

DQKEGMHAFLEKRPPNYQGK

>PA14_43530 | Max Lysine: 3

EEMGLPYRVHALSFDKKEQK

>PA14_43640 | Max Lysine: 3

KVLQPHQAALTDKLLVSLTK

>PA14_43660 | Max Lysine: 3

DKQLYNSQSIKSLLLGSYAK

>PA14_43730 | Max Lysine: 3

TEVPAKLGTKFGMRYQLSGK

>PA14_43740 | Max Lysine: 3

AMDKGRVDHVMLSGVAVAKK

>PA14_43760 | Max Lysine: 3

HFKTRLEKLALRQRRIIELK

>PA14_43790 | Max Lysine: 3

LNIDGKAPLKPRSEYRLVGK

>PA14_43900 | Max Lysine: 3

ALLLAASLGLAACDKKEEDK

>PA14_43940 | Max Lysine: 3

KRMGHAGAIISGGKGTADEK

>PA14_44020 | Max Lysine: 3

GKNGLACITPLSAAGLKGGK

>PA14_44030 | Max Lysine: 3

VKEVLAGNGVGPNKDHVLLK

>PA14_44190 | Max Lysine: 3

KAFSVAKLGIAEDPGFQLDK

>PA14_44300 | Max Lysine: 3

MWTTLKAGRPWMGIVKNRCK

>PA14_44311 | Max Lysine: 3

APVGERYRKVEVDDLKELAK

>PA14_44340 | Max Lysine: 3

VVWVAYAVVFFGTLVKRKVK

>PA14_44360 | Max Lysine: 3

ALLWLVFATRKGEAKGTTEK

>PA14_44370 | Max Lysine: 3

IVWITYAIVFFGTIVKRKVK

>PA14_44450 | Max Lysine: 3

FDDEDPKHQAGVDEAEKPSK

>PA14_44490 | Max Lysine: 3

TVEDMDSLDEIVKRGRPLKK

>PA14_44500 | Max Lysine: 3

GSAVAYALNKPLVLFRKQGK

>PA14_44570 | Max Lysine: 3

PGGIKTNIAKTARMNESMAK

>PA14_44680 | Max Lysine: 3

MRLKSIKLAGFKSFVDPTTV

>PA14_44700 | Max Lysine: 3

AYKHNFLNAWRLEAERLKRK

>PA14_44740 | Max Lysine: 3

VARSLGKDPLEVRKLNYYGK

>PA14_44760 | Max Lysine: 3

LARNDFAYFGLIGSKTKRVK

>PA14_44770 | Max Lysine: 3

NLFLGSGLFDLPKLERYKVK

>PA14_44800 | Max Lysine: 3

QRAHLVKEDGRIENLSKAMK

>PA14_44840 | Max Lysine: 3

TELNDAYKARFGFPFIKAVK

>PA14_44850 | Max Lysine: 3

KEGVFDDSGKWMDGWETRRK

>PA14_44890 | Max Lysine: 3

GQPTGQRVHKPVVITKVFDK

>PA14_44980 | Max Lysine: 3

DKGFAATKTSDIAARAGLPK

>PA14_45020 | Max Lysine: 3

KIGFIGTGIMGKPMAQNLQK

>PA14_45030 | Max Lysine: 3

LPAEGIGLADKQAVNKALLK

>PA14_45050 | Max Lysine: 3

MTADKKAKILATLGPATRSR

>PA14_45300 | Max Lysine: 3

YLIDKQGIIRHKIVGVVDQK

>PA14_45310 | Max Lysine: 3

KGSIHVSRDGKEIATLHPEK

>PA14_45400 | Max Lysine: 3

KAEVLAVKEGLANNFQVLLK

>PA14_45520 | Max Lysine: 3

MKVWAVANQKGGVGKTTSSI

>PA14_45540 | Max Lysine: 3

PNDAAFDIVEKVAKILAPYK

>PA14_45580 | Max Lysine: 3

KNFEDISRNPDKVRQLLCEK

>PA14_45610 | Max Lysine: 3

GQVIKRVTKLVTEVESNLVK

>PA14_45630 | Max Lysine: 3

LMQAGMIGLLEAAKKYDAGK

>PA14_45640 | Max Lysine: 3

SVRKAVQKQRAVYEAFPRSK

>PA14_45660 | Max Lysine: 3

PAGAGKTTTLAKMAARYVLK

>PA14_45680 | Max Lysine: 3

LVDRNQGGQLLARIKGVRKK

>PA14_45700 | Max Lysine: 3

EAWKLGRKGIDDGVLVLVAK

>PA14_45710 | Max Lysine: 3

TLDIEAFSDKLFQAWKLGRK

>PA14_45780 | Max Lysine: 3

ATPEFAQRLLELLNKDPKGK

>PA14_45790 | Max Lysine: 3

NEKFGIRLTDVISPSERIKK

>PA14_45880 | Max Lysine: 3

VVEVAIKRLRAKLDGPFENK

>PA14_45920 | Max Lysine: 3

GGRRTGDSKKLSRWQDLLTK

>PA14_45960 | Max Lysine: 3

GKLEWSAILQKMASDLGFSK

>PA14_45970 | Max Lysine: 3

ALKRADVGVAMGNKGTEAAK

>PA14_46030 | Max Lysine: 3

LEASYNPVYDADGKLFKVVK

>PA14_46110 | Max Lysine: 3

IGLGIVGLSLFLAKPLLKLK

>PA14_46140 | Max Lysine: 3

EHGIGLEKKEALGRYADPLK

>PA14_46250 | Max Lysine: 3

LGRVSFLGHELFKSWGKLRK

>PA14_46270 | Max Lysine: 3

KQAYQQCYAASPAFRAKTHK

>PA14_46300 | Max Lysine: 3

ELKRLMGSKTEYRELYGRLK

>PA14_46420 | Max Lysine: 3

DPVRQARQEKSGKQPGDPRK

>PA14_46450 | Max Lysine: 3

KRLLPGKHLAELYTSIGFYK

>PA14_46560 | Max Lysine: 3

VAAFKALGGGWKVPEALAAK

>PA14_46590 | Max Lysine: 3

PSEVLEEFYQARKKISDEAK

>PA14_46640 | Max Lysine: 3

EKPDGKDWQDMTGYRVPPLK

>PA14_46880 | Max Lysine: 3

LARKSVYGLALGGLDKALDK

>PA14_46960 | Max Lysine: 3

MDGKPYFLDYREVAPKAASK

>PA14_46970 | Max Lysine: 3

EILSGRDASKMVNIKTEAFK

>PA14_46980 | Max Lysine: 3

IIGVAVVKVKMDALEERWEK

>PA14_47040 | Max Lysine: 3

IGFSILIEVFNQVARKRRKK

>PA14_47120 | Max Lysine: 3

AIQKGTNTKVTLEPATSYMK

>PA14_47140 | Max Lysine: 3

KWEFLGRRLELDAALFQVKK

>PA14_47150 | Max Lysine: 3

MKLKRYLLVAKPGIIFGNLI

>PA14_47210 | Max Lysine: 3

RFKAIATSEQGFQDWVAKVK

>PA14_47300 | Max Lysine: 3

PRDGKVLVLINGAYGKRLAK

>PA14_47360 | Max Lysine: 3

AKWVAKLRATRTDANLLLLK

>PA14_47380 | Max Lysine: 3

PINPKRLPNSAQDEKTGLFK

>PA14_47410 | Max Lysine: 3

MAAKVEPFWKRKTLAQLDQD

>PA14_47420 | Max Lysine: 3

MAHTIHGKKKLLARVRRIAG

>PA14_47450 | Max Lysine: 3

MKRICSVYKSPRKNEMYLYV

>PA14_47460 | Max Lysine: 3

AHSMGYSKLVKEVLDIDLPK

>PA14_47490 | Max Lysine: 3

RKANADFLHRHPWLKAQLAK

>PA14_47550 | Max Lysine: 3

QLTKLRSKESIDLCQRYAGK

>PA14_47600 | Max Lysine: 3

ALGPRNGVSCGALEKKMGIK

>PA14_47610 | Max Lysine: 3

AVAKEMGITKGGVQYCFGTK

>PA14_47960 | Max Lysine: 3

VHKAYGQFEVVKGVDLRVDK

>PA14_48060 | Max Lysine: 3

SQNQKINLNEKALSDVGGLK

>PA14_48090 | Max Lysine: 3

EKSHYDLDDKVRETLNQVRK

>PA14_48100 | Max Lysine: 3

KQPYYALRAQVDAAGMGKLK

>PA14_48450 | Max Lysine: 3

PGMDKGQVEAELKALLGLRK

>PA14_48520 | Max Lysine: 3

LPFAALLTACDDGKKEPPPK

>PA14_48530 | Max Lysine: 3

ALLVRKRNGVKQLLCAWTGK

>PA14_48570 | Max Lysine: 3

LTPFEKSASELKYHRHGPGK

>PA14_48590 | Max Lysine: 3

MKQFDKHADAYNVVRGKIAY

>PA14_48610 | Max Lysine: 3

KAPLVALLEQREKRWQAQPK

>PA14_48680 | Max Lysine: 3

TFPGLVRKVLLKLASLQLGK

>PA14_48700 | Max Lysine: 3

DPETNIETAKRVRKLYPHLK

>PA14_48800 | Max Lysine: 3

SYMEAKRVLEPGYKDALTSK

>PA14_48840 | Max Lysine: 3

LEKHGLSGSVVRLEKVLHLK

>PA14_48860 | Max Lysine: 3

DMVAALKAQEIIDKPPTSLK

>PA14_48890 | Max Lysine: 3

MKKVKPIHQNRLLLQPDGQL

>PA14_48940 | Max Lysine: 3

MKAMKQRIAKFSPVASFRNL

>PA14_48970 | Max Lysine: 3

PRPAQQPQGQDKQDKSPDAK

>PA14_48990 | Max Lysine: 3

KHDPSRRPPKYWHVVYDSGK

>PA14_49010 | Max Lysine: 3

HETVRGWIRKGYIPAYKIGK

>PA14_49030 | Max Lysine: 3

MSKRLVLFNHKGGVSKTTTV

>PA14_49070 | Max Lysine: 3

SPKPEEQRALKREIEMEFEK

>PA14_49080 | Max Lysine: 3

VERGMQGFERGRNLKKLGMK

>PA14_49130 | Max Lysine: 3

YPETAVAMKPFGDGFVKLIK

>PA14_49160 | Max Lysine: 3

KNFVVDVIKIDGSFVRNLDK

>PA14_49200 | Max Lysine: 3

NNIQKSKSLNRNLNSPNLDK

>PA14_49280 | Max Lysine: 3

ANYLKKAGWRTGQPWGYEVK

>PA14_49300 | Max Lysine: 3

PQYDNLAGKLARRAVLTSKK

>PA14_49330 | Max Lysine: 3

SFHGALGTATPASKGSVKAK

>PA14_49340 | Max Lysine: 3

TLAFSLKESLFKALYPLVGK

>PA14_49350 | Max Lysine: 3

DCIDSVAAKAALIAWCKRRK

>PA14_49360 | Max Lysine: 3

YNADRKAKFNFTGPVVPIPK

>PA14_49410 | Max Lysine: 3

KVEFSVIQGQKGLQAEDVSK

>PA14_49440 | Max Lysine: 3

RPKIGDDPMHPRLIKTVRSK

>PA14_49470 | Max Lysine: 3

IFNMYHEIPSVAKKASWGLK

>PA14_49520 | Max Lysine: 3

FGNPFRGFTLAMEADFKKRK

>PA14_49540 | Max Lysine: 3

WRAWFVKDHVKSLTTQRGSK

>PA14_49560 | Max Lysine: 3

EGKIYRVLAGNPAKHDLDIK

>PA14_49590 | Max Lysine: 3

KRLGYRLCERGKGGFRLTAK

>PA14_49610 | Max Lysine: 3

KAFSVAKLGIAEDPSFELDK

>PA14_49690 | Max Lysine: 3

QIGVIKGKAAEVDLFPMLSK

>PA14_49760 | Max Lysine: 3

RLLKRLAGPAVASVASGKAK

>PA14_49800 | Max Lysine: 3

EEVVGKALQGLRDKAVIATK

>PA14_49910 | Max Lysine: 3

EKDAETGQRHLHEVSPFMKK

>PA14_49960 | Max Lysine: 3

EKAERADEKAICANRALNDK

>PA14_50000 | Max Lysine: 3

DPSIKSSLAFLRKTPWAREK

>PA14_50010 | Max Lysine: 3

DLDKLQGKIVRLYPDGGVPK

>PA14_50080 | Max Lysine: 3

KLVERYLEEARQAEDKREQK

>PA14_50100 | Max Lysine: 3

IGEPPATKGYPPSVFAKLPK

>PA14_50110 | Max Lysine: 3

MVPHDKDKDNPSELIRGKDV

>PA14_50140 | Max Lysine: 3

DALIDSQVPAKSGGAGMLKK

>PA14_50220 | Max Lysine: 3

NKTQNVDVRIIAATHKNLEK

>PA14_50290 | Max Lysine: 3

GQVKNIAIAAGDSAKAIAEK

>PA14_50310 | Max Lysine: 3

GIGSRLGLNKPKCLVEVGGK

>PA14_50340 | Max Lysine: 3

KRQPDQEKRSLLNTVSDLRK

>PA14_50380 | Max Lysine: 3

NRLNQLKVGKDRDGEANIRK

>PA14_50440 | Max Lysine: 3

VVAVVDRIKLVNPNLKQMEK

>PA14_50500 | Max Lysine: 3

RVAQKHGLHPKFGAITRVHK

>PA14_50510 | Max Lysine: 3

KVAAWLSGGGLSKVGEDAAK

>PA14_50540 | Max Lysine: 3

RIPLKLGRGVGYKVNGSGLK

>PA14_50550 | Max Lysine: 3

NLKVEEKQVVSMIGPNGAGK

>PA14_50560 | Max Lysine: 3

KVSTYYGKIQALHDVSVEVK

>PA14_50600 | Max Lysine: 3

ERLGCSKGQLSKRIGVLEQK

>PA14_50610 | Max Lysine: 3

AKATLHWKPLTFTQREACMK

>PA14_50700 | Max Lysine: 3

VPDKAALIAEIKQRYEAPLK

>PA14_50880 | Max Lysine: 3

SSKVDAADSEKLNGIAERLK

>PA14_50890 | Max Lysine: 3

KQVPDWLNAKFWANPENFGK

>PA14_50940 | Max Lysine: 3

WMTGSFEDPDVKLLKVEVVK

>PA14_50980 | Max Lysine: 3

RKAWQWGKLHTYEWRSDSSK

>PA14_51000 | Max Lysine: 3

QALKQHVTSLRNHVKGLSGK

>PA14_51080 | Max Lysine: 3

FRRHGVKVIHKCTAVRHALK

>PA14_51090 | Max Lysine: 3

AETPEQKARVDKLQRMTPEK

>PA14_51160 | Max Lysine: 3

RVTGLVDKGADKGALLYSEK

>PA14_51205 | Max Lysine: 3

MTKDATTKGTSSKDVGAVVN

>PA14_51220 | Max Lysine: 3

RAHPNYRRARQLILNKALKK

>PA14_51270 | Max Lysine: 3

SKVPNIIGIKEATGDLQRAK

>PA14_51300 | Max Lysine: 3

VMIYLVLVPILVFFFLKDKK

>PA14_51310 | Max Lysine: 3

LPLLKAKLELNRLPSGGVLK

>PA14_51330 | Max Lysine: 3

WDGACIVHEEFKAKQLEDMK

>PA14_51440 | Max Lysine: 3

GKRSRFELPLDFQGTEFQKK

>PA14_51450 | Max Lysine: 3

SKDGGNSGSVGLGWDTSKAK

>PA14_51460 | Max Lysine: 3

VKLKAGNASYVVDADMVAPK

>PA14_51470 | Max Lysine: 3

LNLNLDCGKTGTGLTTVKLK

>PA14_51490 | Max Lysine: 3

LALLDKHKHPHPPQLPHALK

>PA14_51540 | Max Lysine: 3

PEFTGKALDQWACQRDIKLK

>PA14_51580 | Max Lysine: 3

STKSKPIARGRGGPFGEVMK

>PA14_51620 | Max Lysine: 3

LRRWVKQLEAERQGVTPKSK

>PA14_51710 | Max Lysine: 3

SSDLKPEAMRALDVHAKDLK

>PA14_51720 | Max Lysine: 3

YIADQSFEKLTGIKGAFSTK

>PA14_51740 | Max Lysine: 3

GVKVDLPKVSSEALPQDNNK

>PA14_51750 | Max Lysine: 3

AKKALDAFEERFWSGIDLSK

>PA14_51800 | Max Lysine: 3

GADKQQVQMMVMHLLKLTQK

>PA14_51820 | Max Lysine: 3

ETFAKADRVRSEFVVKITGK

>PA14_51880 | Max Lysine: 3

DDYSRAGGAVKVRISKTMLK

>PA14_51900 | Max Lysine: 3

TKTITALVDGFQLPIEKTIK

>PA14_52010 | Max Lysine: 3

LLLAASKSPRELQVKLPDLK

>PA14_52040 | Max Lysine: 3

PTRIYVKPLLQLIKQTGAVK

>PA14_52060 | Max Lysine: 3

ARQGLGKGKQIELDFDWSQK

>PA14_52070 | Max Lysine: 3

TKGTVSQSLKVLEGRGLISK

>PA14_52120 | Max Lysine: 3

DAHAYRIKLEKEEELRQIGK

>PA14_52150 | Max Lysine: 3

RAALNNNEAGFGSFFKKGWK

>PA14_52180 | Max Lysine: 3

REGKITLEAVNKHFGPVVAK

>PA14_52210 | Max Lysine: 3

AIKGYKMILIMPDNSTAERK

>PA14_52260 | Max Lysine: 3

TLPDEQVEAKPACTRKLQRK

>PA14_52280 | Max Lysine: 3

GTGGSIKADDVLLVSLKGSK

>PA14_52310 | Max Lysine: 3

VSAGVAPNKFLAKIASDWRK

>PA14_52350 | Max Lysine: 3

KLGEEARVDLLRFDLENKGK

>PA14_52370 | Max Lysine: 3

KSPEQSAADLSKLMQHYREK

>PA14_52380 | Max Lysine: 3

ACIELKGNFPKGSETRELLK

>PA14_52430 | Max Lysine: 3

ELPLSNNMILKKLRVAFELK

>PA14_52460 | Max Lysine: 3

KELPGHTDKLFVVDYDGVLK

>PA14_52570 | Max Lysine: 3

APKEVAVHREEIYQRIQKEK

>PA14_52580 | Max Lysine: 3

GGTSVGTVERIEQVAEKVKK

>PA14_52600 | Max Lysine: 3

KDCFLGLEKRAYTRATTSQK

>PA14_52670 | Max Lysine: 3

NCVVFKPSELTPKVAELTLK

>PA14_52770 | Max Lysine: 3

KATPHGEIEAAKAMGMSRLK

>PA14_52780 | Max Lysine: 3

VKATALISVVGLQDMMFKAK

>PA14_52840 | Max Lysine: 3

KFSHVVAENTPKGQGALLFK

>PA14_52870 | Max Lysine: 3

SKQSLSLDLKQAEALPLLEK

>PA14_52940 | Max Lysine: 3

KRLEHDLRERVVFAISGKSK

>PA14_52980 | Max Lysine: 3

LPELVRANKARINGLRVKVK

>PA14_52990 | Max Lysine: 3

NPWFAEFTHTYGKLGLKASK

>PA14_53000 | Max Lysine: 3

IEVRDGIMQLEKVYLFKNFK

>PA14_53040 | Max Lysine: 3

PGDAKLDTRSVDYKCENGRK

>PA14_53070 | Max Lysine: 3

RKLRANGVKFMSTPDTYYEK

>PA14_53140 | Max Lysine: 3

VQQWKTEIHAINEKVTPAAK

>PA14_53190 | Max Lysine: 3

KVVLVSPQFAGLNAVKRHQK

>PA14_53220 | Max Lysine: 3

LVTALNPHIGYDKAAEIAKK

>PA14_53230 | Max Lysine: 3

TPLEGDGKRILLIVGSPKRK

>PA14_53250 | Max Lysine: 3

KLENGTYRMNCPLPQGKTGK

>PA14_53300 | Max Lysine: 3

TEEHGEVCPAGWRKGQKGMK

>PA14_53310 | Max Lysine: 3

EPQLRFDITKKRLLYIQGEK

>PA14_53340 | Max Lysine: 3

DLGMIFQAVHLKVLARLKAK

>PA14_53360 | Max Lysine: 3

NDPRALKRQDGKPVWYQNYK

>PA14_53390 | Max Lysine: 3

ADGEDKPVALSPSFEKSAEK

>PA14_53420 | Max Lysine: 3

DLPLSPFKGKVLLVVNVASK

>PA14_53510 | Max Lysine: 3

HFLASGEMKKRTGASLHLHK

>PA14_53530 | Max Lysine: 3

DTTKGLVHMLKRDGLLWNFK

>PA14_53560 | Max Lysine: 3

DIQKPWDSHDYQVLIDALKK

>PA14_53570 | Max Lysine: 3

LKDLGQIDYGNPLAGVKLLK

>PA14_53580 | Max Lysine: 3

PTPKSAQRKTESCGELLDHK

>PA14_53650 | Max Lysine: 3

GWISKYWDEDPEHPPFKPGK

>PA14_53660 | Max Lysine: 3

YTSGKKSRDAGAWLFDIYGK

>PA14_53690 | Max Lysine: 3

ASPPPERFKLAPLGSVVRKK

>PA14_53770 | Max Lysine: 3

IESAKSLEYDIGEPGVRKRK

>PA14_53820 | Max Lysine: 3

NALKQLAKNILQRYPDMTPK

>PA14_53840 | Max Lysine: 3

PEQYDDSGKMEEVINRVRKK

>PA14_53940 | Max Lysine: 3

KSMIKFGAAAMHIEDQVGAK

>PA14_53970 | Max Lysine: 3

DRLGLVRKPWVKTSLAPGSK

>PA14_54000 | Max Lysine: 3

FYDVLFSHTSKDLATKPEDK

>PA14_54170 | Max Lysine: 3

AMKLMGKQFVLGRTISEALK

>PA14_54180 | Max Lysine: 3

LEGPFTSLKLDAAYTKYEHK

>PA14_54240 | Max Lysine: 3

KTDTQRVGSFWADVFNFRKK

>PA14_54340 | Max Lysine: 3

KALEVQLEDNQFRAHLKYEK

>PA14_54350 | Max Lysine: 3

PDPAVLEKLNKEPLLVEYGK

>PA14_54430 | Max Lysine: 3

RVQRGDKRAFDLLVLKYQHK

>PA14_54480 | Max Lysine: 3

AGELLEAQLADLKKYAVFSK

>PA14_54640 | Max Lysine: 3

AVEPYKASSFDLTHKLTVEK

>PA14_54700 | Max Lysine: 3

NRLLLKEVSAVRLVFEGKLK

>PA14_54810 | Max Lysine: 3

SPANAPLPPADIKARLKSMK

>PA14_54820 | Max Lysine: 3

DYSRPSRDKRLWVFDLKARK

>PA14_54880 | Max Lysine: 3

ILQKVTLGGTGKEYGDRHPK

>PA14_54930 | Max Lysine: 3

KYWQDSLAKLPPGPQLPLVK

>PA14_54940 | Max Lysine: 3

LVKLWLEARKWDTSDLSSLK

>PA14_55000 | Max Lysine: 3

KLSPDLTIGLRTYTEPFAKK

>PA14_55050 | Max Lysine: 3

FPVAPATKLTLGGRYTWEKK

>PA14_55060 | Max Lysine: 3

PEFTGKALDQWAYQRDIKLK

>PA14_55090 | Max Lysine: 3

NCIARLMSAKRIQGWSRRKK

>PA14_55180 | Max Lysine: 3

ERGRTELVNKALKYAVVADK

>PA14_55200 | Max Lysine: 3

ERWRKGQPKGPLDGLPVTIK

>PA14_55300 | Max Lysine: 3

EQLGKYKQAYDELLVMARAK

>PA14_55320 | Max Lysine: 3

LGGGFGKKDLWLDQGYLTWK

>PA14_55340 | Max Lysine: 3

IQINLPKASATVALSQPKTK

>PA14_55380 | Max Lysine: 3

DDEEFDRKRYRAKSGFGVLK

>PA14_55390 | Max Lysine: 3

KVGMQELDQRLSKIVEAARK

>PA14_55410 | Max Lysine: 3

GKAAFLNNDYTKFVAGTTNK

>PA14_55450 | Max Lysine: 3

VDIPGIGKILDLKVLARALK

>PA14_55550 | Max Lysine: 3

QSAMVKLFLASYDDFKARLK

>PA14_55560 | Max Lysine: 3

AQPLTILLLPKTTGKAMECK

>PA14_55580 | Max Lysine: 3

LGWLFVSEGSKLGAAFLFKK

>PA14_55610 | Max Lysine: 3

KLLRGQLKARRCRDSRELAK

>PA14_55650 | Max Lysine: 3

KSQQSLQDLETQQMLAANKK

>PA14_55670 | Max Lysine: 3

GEQPANLGKFGQTRIKLTAK

>PA14_55720 | Max Lysine: 3

IVATLAYAVYSVLLKKWSLK

>PA14_55760 | Max Lysine: 3

KPWRKRPTVQEQAFDRELQK

>PA14_55800 | Max Lysine: 3

CLLCIALFILVKLFKRSVEK

>PA14_55810 | Max Lysine: 3

RDIHKIKYEVHGNSQPSALK

>PA14_55860 | Max Lysine: 3

LFAAGIGFLLPKQVLKHFAK

>PA14_55900 | Max Lysine: 3

AKNLGKSLFEVAPRDPLAAK

>PA14_56000 | Max Lysine: 3

MIKSLKFSHKILLAASLVVF

>PA14_56010 | Max Lysine: 3

MIKSLKFSHKILLAAALVVI

>PA14_56060 | Max Lysine: 3

MEWRITDSEVKKRVVLMASK

>PA14_56090 | Max Lysine: 3

YDFRIGDVISEAWAKIKGVK

>PA14_56210 | Max Lysine: 3

KSARELLATWKPQQNLDLYK

>PA14_56280 | Max Lysine: 3

MRTDKPPLKQRALQKLLLRR

>PA14_56620 | Max Lysine: 3

KALSHPVRRDMLRWLKEPEK

>PA14_56730 | Max Lysine: 3

SGLSTGTAKTSVEGLKLGGK

>PA14_56780 | Max Lysine: 3

ADAINAAFGSFDKFKEEFTK

>PA14_56800 | Max Lysine: 3

RIEVEEKRSGREKAWRTVDK

>PA14_56810 | Max Lysine: 3

LVRLKHAVGKAWANIDVLLK

>PA14_56890 | Max Lysine: 3

KSNEVKSQLPPDAEDPVLQK

>PA14_56900 | Max Lysine: 3

LWAEKTIAHYLKLGIDPLTK

>PA14_56930 | Max Lysine: 3

VDLDFSHKYDREHAEAYLKK

>PA14_57020 | Max Lysine: 3

AVKVGDKVVFGPYSGSNAIK

>PA14_57040 | Max Lysine: 3

KNARELLLKEYRAVLSTHSK

>PA14_57080 | Max Lysine: 3

MGVHKADRKGKTVEHAWAGS

>PA14_57140 | Max Lysine: 3

ECSSSSFKRLHDGLNLKAFK

>PA14_57240 | Max Lysine: 3

NQKNLVEVGDLVKRGQVLAK

>PA14_57275 | Max Lysine: 3

NEKLLTILGKDASLLAAFAK

>PA14_57290 | Max Lysine: 3

IKIRYACALAKLAGAGETIK

>PA14_57300 | Max Lysine: 3

LVAKEPLSVRLPKADFSFLK

>PA14_57330 | Max Lysine: 3

TNIDADHMATYGGDFNKLKK

>PA14_57370 | Max Lysine: 3

FDLNKPDFKAFGLIEEDGQK

>PA14_57380 | Max Lysine: 3

VDDYRKVIEKNSRGLPSRWK

>PA14_57390 | Max Lysine: 3

RKFTGPLAAMTGSSGKTTVK

>PA14_57450 | Max Lysine: 3

ISFHSLEDRIVKLFMRKHAK

>PA14_57460 | Max Lysine: 3

RLREYAKLDKRAMLVGQLNK

>PA14_57470 | Max Lysine: 3

AELPLKKAAALAAEITGVRK

>PA14_57480 | Max Lysine: 3

QQNPEHPAAKHLPAALEKLK

>PA14_57540 | Max Lysine: 3

MEKLVFTGAKIGDHMDIGMK

>PA14_57570 | Max Lysine: 3

WFPSAKAKAAGAPVQVNVGK

>PA14_57630 | Max Lysine: 3

QRLRIETAKGMLSSTRKSIK

>PA14_57690 | Max Lysine: 3

APKSPDSPGTLDATYGLKGK

>PA14_57740 | Max Lysine: 3

GYFWKGENPCVVGMKQRRLK

>PA14_57770 | Max Lysine: 3

KFWSPFVKDLVPYVPGEQPK

>PA14_57800 | Max Lysine: 3

GAPEPKGRLRVATKFVNVAK

>PA14_57810 | Max Lysine: 3

GGRVKLKDTDPTILEAVLQK

>PA14_57830 | Max Lysine: 3

KAGKVLSVRALPDDMREIAK

>PA14_57910 | Max Lysine: 3

PKGKEVELIDDVRVARTDAK

>PA14_57930 | Max Lysine: 3

MATLKAQHLAKSYKGRQVVR

>PA14_58030 | Max Lysine: 3

NVSRLLADKAIASFKVNQGK

>PA14_58100 | Max Lysine: 3

DLYGVEDEIQKALERKVLLK

>PA14_58230 | Max Lysine: 3

EKQETFLVPMRKPGELTLVK

>PA14_58270 | Max Lysine: 3

ELDDELGKLFGRVIGAKEAK

>PA14_58320 | Max Lysine: 3

GVPLAIAEQIGKPFFTTKGK

>PA14_58360 | Max Lysine: 3

RYRKDAQVRYAANPDYWKGK

>PA14_58420 | Max Lysine: 3

HKPLDDPRVRQAINLALDKK

>PA14_58490 | Max Lysine: 3

EEPTSGSLKIAGQEVKGASK

>PA14_58570 | Max Lysine: 3

AIAAPQAAHADEAGQKKTDK

>PA14_58580 | Max Lysine: 3

EQAEWADGKATAGYQSAKAK

>PA14_58610 | Max Lysine: 3

LMKQAGYKTFWITNQQTMTK

>PA14_58650 | Max Lysine: 3

LQGKGARAEKLLGSAQVFIK

>PA14_58700 | Max Lysine: 3

TTVKLLDTRKTLPGLRLAQK

>PA14_58770 | Max Lysine: 3

LVSYLALGGKCSSCKAAIGK

>PA14_58790 | Max Lysine: 3

APVEWKSDNKYRPFCSDRCK

>PA14_58820 | Max Lysine: 3

VARDMDGSKLVKAALAERPK

>PA14_58870 | Max Lysine: 3

GKRGKAQVTLVDANLTHIWK

>PA14_58890 | Max Lysine: 3

QLSSFIDQGAVKFLKHPNTK

>PA14_58910 | Max Lysine: 3

MNAKATSVVSTKGGVGKSTT

>PA14_58980 | Max Lysine: 3

AKAVAHVGSVIIESAKVEVK

>PA14_59060 | Max Lysine: 3

TEDKFVVRMPLGLRDQLKQK

>PA14_59090 | Max Lysine: 3

LKDGLYPSGKSSQSQAGLQK

>PA14_59100 | Max Lysine: 3

VRMPNSYSTYTYKQDSVCKK

>PA14_59140 | Max Lysine: 3

TAFQALQHSASLKGLLKPFK

>PA14_59150 | Max Lysine: 3

TKGMRVKVEGRAIMDRWPDK

>PA14_59190 | Max Lysine: 3

YWFSRFEFSLKENGYLKNYK

>PA14_59230 | Max Lysine: 3

KYYWAKFFWGAFFFVLVAWK

>PA14_59240 | Max Lysine: 3

HAPAKKPESTTVLPPAAPAK

>PA14_59250 | Max Lysine: 3

TDKDQLGIDWNLVYKSLNNK

>PA14_59270 | Max Lysine: 3

FWRPLSSQRQYMKEARKLGK

>PA14_59320 | Max Lysine: 3

ITTSKLPQDACITLATKIAK

>PA14_59350 | Max Lysine: 3

EQHAAQQQQQVAKAAEKYLK

>PA14_59370 | Max Lysine: 3

GRKSAAGRNPVKGFPSRVWK

>PA14_59480 | Max Lysine: 3

QLPTVVPAIPPKSPSPQKSK

>PA14_59550 | Max Lysine: 3

TASASRNISALKRDAKRLQK

>PA14_59650 | Max Lysine: 3

ERVRRWAILAGIDPKKVRSK

>PA14_59680 | Max Lysine: 3

RKQVLELHAKGRLQAPASLK

>PA14_59690 | Max Lysine: 3

ARRLEKQLEFAPFPLKLVAK

>PA14_59780 | Max Lysine: 3

MKSASALEHDNKLLLKWTTL

>PA14_59840 | Max Lysine: 3

QAVNISTLLALINSAKDTKK

>PA14_59880 | Max Lysine: 3

LGVAWHTYGTYHAIHDGKKK

>PA14_59920 | Max Lysine: 3

ESRQLKDQISKVIQENDSLK

>PA14_59960 | Max Lysine: 3

DQARLEKCAKDNELIDSDIK

>PA14_59990 | Max Lysine: 3

YKVVGICYWLLCGPHGCKVK

>PA14_60020 | Max Lysine: 3

DRIKDQVDPDLMTSFLKWAK

>PA14_60080 | Max Lysine: 3

KALDWVISEHGASMRARKQK

>PA14_60090 | Max Lysine: 3

SKPATLVSFEITSKATTNTK

>PA14_60100 | Max Lysine: 3

KEIQQNLVLKSGEQVITCSK

>PA14_60120 | Max Lysine: 3

MIYSPHSLLKLVRDGKLIKH

>PA14_60210 | Max Lysine: 3

LDKDTTGLMVVAKTLEAHTK

>PA14_60230 | Max Lysine: 3

QQAQDDLNNKSYNSAVTKLK

>PA14_60260 | Max Lysine: 3

MESEFFGHKKGSFTGAIEDK

>PA14_60270 | Max Lysine: 3

KPLGLELPVVPVKGQMILYK

>PA14_60320 | Max Lysine: 3

IGKLHMRNTSGTTVKSSTGK

>PA14_60330 | Max Lysine: 3

KEAEGRGLKVFDATCPLVTK

>PA14_60350 | Max Lysine: 3

KLEDGNVVDSTFDKQPASFK

>PA14_60360 | Max Lysine: 3

ALIAIVVSAILVVWLKRLKK

>PA14_60380 | Max Lysine: 3

QKLGRQLGSPTANIQLKRRK

>PA14_60390 | Max Lysine: 3

QLRKQRLYLPQPGWAKFLVK

>PA14_60410 | Max Lysine: 3

VFKWVGPNDKIVVEAFDDPK

>PA14_60420 | Max Lysine: 3

DGAVKAVSQDHKSLLPVGVK

>PA14_60445 | Max Lysine: 3

KAGDGGNGLMSFRREKFIEK

>PA14_60470 | Max Lysine: 3

GGKRLRPLLVLLAGKTLGYK

>PA14_60590 | Max Lysine: 3

SFKYLYFWGHRPATDDKVGK

>PA14_60710 | Max Lysine: 3

LTTLPMGGGKGGSDFDPKGK

>PA14_60730 | Max Lysine: 3

WKPTDRDTLGFAYHAKIRNK

>PA14_60750 | Max Lysine: 3

PWKLTVSSTTAGKVDGVGFK

>PA14_60820 | Max Lysine: 3

EEKALRALANSSNEALKLAK

>PA14_60890 | Max Lysine: 3

EHVIAAKAVCFKEALQPEFK

>PA14_60920 | Max Lysine: 3

LGAGKTTLLKYLLQAEHGMK

>PA14_60950 | Max Lysine: 3

LFYAIKVGIAAWGKSERTDK

>PA14_61010 | Max Lysine: 3

QGLKQQRVQLKDEIAERLRK

>PA14_61090 | Max Lysine: 3

YHAKLKRLFRGVEQVAANPK

>PA14_61130 | Max Lysine: 3

PQQVAQLKTESASVHTVKVK

>PA14_61190 | Max Lysine: 3

RFKPRSEPQAKPEAPLVVPK

>PA14_61200 | Max Lysine: 3

LVSKSGDIQIAGKIDVSGPK

>PA14_61260 | Max Lysine: 3

VALWTLVPDKLDDEESGLKK

>PA14_61270 | Max Lysine: 3

WNKGRMALVEQLHSKDFLYK

>PA14_61290 | Max Lysine: 3

ANCGPGGKIIFYTGLIDKLK

>PA14_61300 | Max Lysine: 3

KLLPTGVRENEFYACPKENK

>PA14_61360 | Max Lysine: 3

KDGDDRTDSAILKMFHMSRK

>PA14_61440 | Max Lysine: 3

QPIPITTPTLLRLKGKPLSK

>PA14_61460 | Max Lysine: 3

VHTAVLVDKEHERKARPDLK

>PA14_61470 | Max Lysine: 3

QYEIPGWAGPVTVEKISGKK

>PA14_61480 | Max Lysine: 3

FVYTFMGLAVKIKGTGFIDK

>PA14_61580 | Max Lysine: 3

KWIEPYTDAKLDELVQRGVK

>PA14_61590 | Max Lysine: 3

AVVNLAGEPIADKPWSHKRK

>PA14_61700 | Max Lysine: 3

MKASLLKKLDVLSDRYEELT

>PA14_61720 | Max Lysine: 3

HKTPDGTPPVEDTAVETKAK

>PA14_61740 | Max Lysine: 3

TWKTHKQQLSELDAWQIDGK

>PA14_61770 | Max Lysine: 3

RAVAKSLGVDLAIIDKRRPK

>PA14_61780 | Max Lysine: 3

NVGGAKETVLIKALQRHPAK

>PA14_61790 | Max Lysine: 3

SLVADRKYFGLVGKFSHQGK

>PA14_61890 | Max Lysine: 3

VEGRGIPISLPKPGAPAAKK

>PA14_61960 | Max Lysine: 3

VLKKLGNDEQALIGVAERAK

>PA14_62100 | Max Lysine: 3

LLAITSNRFAMRKLGSRWKK

>PA14_62110 | Max Lysine: 3

QNGAPLRLVVPWKYGFKSIK

>PA14_62150 | Max Lysine: 3

HDEVIEQITKNLNKLIEVVK

>PA14_62190 | Max Lysine: 3

QAREKQGDSAGAALARQKAK

>PA14_62200 | Max Lysine: 3

RNSKARPAPGLNKWLGWALK

>PA14_62270 | Max Lysine: 3

DYAWYTPAQPDKDYCAEMKK

>PA14_62300 | Max Lysine: 3

RVETLSAKPDLEALESNLKK

>PA14_62330 | Max Lysine: 3

LREGWAALKDTHHFHALLKK

>PA14_62390 | Max Lysine: 3

DANCGCCKDWIKHLEANGFK

>PA14_62400 | Max Lysine: 3

VWAKIPEPYAHLGSLEFAKK

>PA14_62440 | Max Lysine: 3

TLIGVAKRAGLMGKDGHMPK

>PA14_62490 | Max Lysine: 3

EEEFSLELRARDRERKLIKK

>PA14_62530 | Max Lysine: 3

SPQEFASQLAKPLGAKTAQK

>PA14_62540 | Max Lysine: 3

SGTGKELVARALHNLSKRAK

>PA14_62600 | Max Lysine: 3

RAENGSKMISVNGAAAHKAK

>PA14_62660 | Max Lysine: 3

NGVTYILTFEANKLKRIEFK

>PA14_62710 | Max Lysine: 3

VIRAVKELAAEAGKPAWDWK

>PA14_62720 | Max Lysine: 3

VALLSANINKLQDHFKANGK

>PA14_62730 | Max Lysine: 3

IEQVPPMYSALKKDGQPLYK

>PA14_62770 | Max Lysine: 3

VEDVQEQKPGAKVGEVIEEK

>PA14_62870 | Max Lysine: 3

ARSKTSQRWLKEHFDDPYVK

>PA14_62900 | Max Lysine: 3

KFPMTVQGARALEEEVKHLK

>PA14_62990 | Max Lysine: 3

NVRRRAEQDVEKAHKFALEK

>PA14_63010 | Max Lysine: 3

IARMLGGVDLTKESLAHAKK

>PA14_63020 | Max Lysine: 3

MVENSELRKAGLKVTLPRVK

>PA14_63030 | Max Lysine: 3

KEGSTTVTQPADQQKPEEQK

>PA14_63050 | Max Lysine: 3

PFTELHGVWQFKALGEKACK

>PA14_63080 | Max Lysine: 3

LRIGAKIGITTFKEVLVELK

>PA14_63100 | Max Lysine: 3

RETRANAELAAKIRHKYRLK

>PA14_63160 | Max Lysine: 3

KRIVELHFGSIGLGKGLEGK

>PA14_63170 | Max Lysine: 3

MNIGEAAKKSGLTPKMIRYY

>PA14_63200 | Max Lysine: 3

YVVRCGDSLRCAKVYKEANK

>PA14_63250 | Max Lysine: 3

NEGLRKILLEANRGKSNLDK

>PA14_63290 | Max Lysine: 3

VGDSKVLCRGVKLPGEIPPK

>PA14_63310 | Max Lysine: 3

NRDALIYRNLLKGHFRKLRK

>PA14_63340 | Max Lysine: 3

TVNYKVATLQVDLFDGKDGK

>PA14_63500 | Max Lysine: 3

DTAIRLMHYYQNCRGKRAKK

>PA14_63580 | Max Lysine: 3

VRNQEEVAKLIDVSKCIGCK

>PA14_63850 | Max Lysine: 3

KQKDESVAALTRGVEFLFRK

>PA14_63880 | Max Lysine: 3

PLMKALGLTHGGFYAHFKSK

>PA14_63920 | Max Lysine: 3

AATKVIDETQPLRALTKAVK

>PA14_63960 | Max Lysine: 3

LFANASKSFKPNGGTDMAGK

>PA14_63990 | Max Lysine: 3

LAPKGGTIVCNGYKDREFIK

>PA14_64030 | Max Lysine: 3

VKLNTNIKNPEEVQPGEAPK

>PA14_64050 | Max Lysine: 3

PMGDRVIKGLALFLKQRLRK

>PA14_64080 | Max Lysine: 3

AATPATNDAASKDPLEHKGK

>PA14_64100 | Max Lysine: 3

MDIRKVKKLIELLEESGIDE

>PA14_64180 | Max Lysine: 3

LGAQIIDINMGCPAKKVCNK

>PA14_64200 | Max Lysine: 3

YVEAKKGEASVSTAIQLQGK

>PA14_64230 | Max Lysine: 3

SNAPSKIIARNAGIKRILAK

>PA14_64390 | Max Lysine: 3

WGVVKADVGLKDGRIQAIGK

>PA14_64400 | Max Lysine: 3

SLREYRKIQLVPFDPDAKRK

>PA14_64440 | Max Lysine: 3

KKYTFPEEYQAWARDYMEVK

>PA14_64450 | Max Lysine: 3

GYEGRLMRSLKSLLGSKLLK

>PA14_64480 | Max Lysine: 3

SGTQKRLMKPGSCNSYILNK

>PA14_64560 | Max Lysine: 3

EPGERSLLKPGAKVVLFPQK

>PA14_64570 | Max Lysine: 3

MRILVIEDDTKTGEYLKKGL

>PA14_64610 | Max Lysine: 3

YAVAIQHLRLDKYAKGRLDK

>PA14_64960 | Max Lysine: 3

LKMTACNGHPVAKISDAPGK

>PA14_64980 | Max Lysine: 3

AARVIVDTYHKTQHKRELPK

>PA14_65030 | Max Lysine: 3

KAPGPGASVPKVVRGEYRVK

>PA14_65090 | Max Lysine: 3

DALQFGKLYKAADQALYRVK

>PA14_65150 | Max Lysine: 3

VILLEKVANLGNLGDKVNIK

>PA14_65190 | Max Lysine: 3

KVYGVHAVEALLRHHPKRVK

>PA14_65230 | Max Lysine: 3

MGKNVVVLGTQWGDEGKGKI

>PA14_65270 | Max Lysine: 3

ESDVKPGLHFKIPYVNQVRK

>PA14_65280 | Max Lysine: 3

LDEAFRKLQDSLNGLFGGKK

>PA14_65300 | Max Lysine: 3

PSAKYLIGSGKVEELHDLVK

>PA14_65350 | Max Lysine: 3

RRKFLRAEKTEFDHLQEVIK

>PA14_65370 | Max Lysine: 3

TRTGDYFIPLRKRTEIARKK

>PA14_65400 | Max Lysine: 3

EKAYVSRYALGRDYHKLIRK

>PA14_65410 | Max Lysine: 3

STLKELAARWAPQVRESFKK

>PA14_65450 | Max Lysine: 3

AFLQANPGSTFMVVLKKAPK

>PA14_65470 | Max Lysine: 3

LEPGQEKLQLEIASSPLLKK

>PA14_65560 | Max Lysine: 3

GLAGLGVAFRAKPLVKQSAK

>PA14_65640 | Max Lysine: 3

AEGGKIEPPPELGSARKLPK

>PA14_65720 | Max Lysine: 3

EEAFWKQAELAHKAWLLANK

>PA14_65750 | Max Lysine: 3

AYLKQDYDPDKDFLPPDLAK

>PA14_65760 | Max Lysine: 3

SGVCYGGKRFYDQGGLAGKK

>PA14_66010 | Max Lysine: 3

AERPVIEYAAAHAKGILVKK

>PA14_66050 | Max Lysine: 3

RTGFRLASERSAKQAKGPRK

>PA14_66060 | Max Lysine: 3

VDVESLLAKVKVLVLSDYGK

>PA14_66090 | Max Lysine: 3

HGDKVLRLRDGSFLKLFRRK

>PA14_66110 | Max Lysine: 3

RKHPTNTYKNARFMVDNVFK

>PA14_66120 | Max Lysine: 3

RKHVDVSRYDRILFFLRFKK

>PA14_66140 | Max Lysine: 3

MSARKQQLLKRHRRNKRIGL

>PA14_66190 | Max Lysine: 3

VGAGEWRGRKVLAKLLVGGK

>PA14_66210 | Max Lysine: 3

KRYRSAGKGLRRYLARPRIK

>PA14_66220 | Max Lysine: 3

VKIHRGIGWGEIAKNLLTAK

>PA14_66260 | Max Lysine: 3

VDGRKIGAGRRGPVTEKLQK

>PA14_66270 | Max Lysine: 3

AKRSLDNQEFFTRLGQKLIK

>PA14_66340 | Max Lysine: 3

KDERQLKRFYQALERSVAPK

>PA14_66350 | Max Lysine: 3

AKLPDAPPGVKGISLFIVPK

>PA14_66380 | Max Lysine: 3

FVYHLNKEKWCIGAALRELK

>PA14_66420 | Max Lysine: 3

ILSKAYRHVIKRRAPRHVAK

>PA14_66460 | Max Lysine: 3

PGLSASAVKTVSAMLQLKRK

>PA14_66610 | Max Lysine: 3

GAGKSTIGRLLAKELHLAFK

>PA14_66670 | Max Lysine: 3

GKPIKDLSLAEMAMIAGLPK

>PA14_66680 | Max Lysine: 3

GNLGPLASKPVMEGKGVLFK

>PA14_66700 | Max Lysine: 3

MDGPLVLRIEPKVLKAFDAK

>PA14_66710 | Max Lysine: 3

KPIHLDVCSECHPFYTGKQK

>PA14_66720 | Max Lysine: 3

DRSEVPEDKLKPALRVLDAK

>PA14_66750 | Max Lysine: 3

KKRFDESPEFADRARELVVK

>PA14_66770 | Max Lysine: 3

MGGDGQVSLGNTVMKGNAKK

>PA14_66820 | Max Lysine: 3

KRFFETGGKSLLDGLGHLAK

>PA14_66840 | Max Lysine: 3

NKYYIFDLSPEKSFVQYALK

>PA14_66890 | Max Lysine: 3

SLGLDAARAKAEKLAERLAK

>PA14_66910 | Max Lysine: 3

LKLALDRLDARVGRLSQKTK

>PA14_66940 | Max Lysine: 3

WSRSRGKLWRKGEESGHVQK

>PA14_66950 | Max Lysine: 3

KNAAPDSSYVASLYHKGLNK

>PA14_67020 | Max Lysine: 3

HAYATLANALQSRKPFKAFK

>PA14_67040 | Max Lysine: 3

MTKKKRSVWPWHLLTGLILL

>PA14_67100 | Max Lysine: 3

AAKMLHKLLNYRVFGDDEGK

>PA14_67200 | Max Lysine: 3

TGWPGGVEKDPQKALDLYQK

>PA14_67220 | Max Lysine: 3

AKNTGEDDKPYQLAEAPGLK

>PA14_67230 | Max Lysine: 3

IHFKAGDKVVIEAGMELTLK

>PA14_67250 | Max Lysine: 3

MKRLWQHCHAATLKGGKYSI

>PA14_67270 | Max Lysine: 3

MSGAQMNKIEVKNVFKIFGP

>PA14_67300 | Max Lysine: 3

VPRYVIEGDPQRKLEAKAPK

>PA14_67320 | Max Lysine: 3

REGLKSSPKLEQARRLLRDK

>PA14_67350 | Max Lysine: 3

RWAALSGDPQDIYKTDAKVK

>PA14_67490 | Max Lysine: 3

APVKRYVEELLAGKEGPLGK

>PA14_67510 | Max Lysine: 3

LDYDDLKRKFALGGGERSEK

>PA14_67670 | Max Lysine: 3

QLSKQETTKLLVRGLAHEIK

>PA14_67680 | Max Lysine: 3

AQELAVEPKLLKAETEEYLK

>PA14_67740 | Max Lysine: 3

AKQLLQRKGVDFQEIACDGK

>PA14_67770 | Max Lysine: 3

LGEYLAKHGKTQLRIAETEK

>PA14_67860 | Max Lysine: 3

KEVKVVQRFHTAELFLALNK

>PA14_67880 | Max Lysine: 3

AKRIIPCLDVDNGRVVKGVK

>PA14_67890 | Max Lysine: 3

TKAVKQPEFVGEACRAFPGK

>PA14_67930 | Max Lysine: 3

VEDIGITLGQAFAKAIGDKK

>PA14_67990 | Max Lysine: 3

LLGREADYPQPKPRKALPQK

>PA14_68000 | Max Lysine: 3

AKGEDIYNNVSRKAWDEWQK

>PA14_68060 | Max Lysine: 3

NPHQGQILVSGEELRLKKSK

>PA14_68110 | Max Lysine: 3

KDRILEKHLAALDITAAQFK

>PA14_68140 | Max Lysine: 3

LFAAFVVYQQLKARPVVIKK

>PA14_68210 | Max Lysine: 3

KIDWPLQDAPLLSEKDRQGK

>PA14_68250 | Max Lysine: 3

RSVAEALGIPRKTLHDKLRK

>PA14_68300 | Max Lysine: 3

ILFAKAKHEVGQPIFTGIEK

>PA14_68350 | Max Lysine: 3

PSPRPKRIFEIRPVKWLLEK

>PA14_68360 | Max Lysine: 3

KVLELSPAEGQALTFKASPK

>PA14_68400 | Max Lysine: 3

VEGDKVIASGEVASQEEKEK

>PA14_68440 | Max Lysine: 3

VAHFWLDSKQPFKNLRALLK

>PA14_68500 | Max Lysine: 3

ATGIDAMVHAIESYTSKLKK

>PA14_68560 | Max Lysine: 3

IGLMVAVGKKAVEPWPRSGK

>PA14_68660 | Max Lysine: 3

IIIQYLEKNGGPHLARTKGK

>PA14_68680 | Max Lysine: 3

IRGDKARGGKGTGLGLAIVK

>PA14_68700 | Max Lysine: 3

ELKKGDEVHMLTTGEFAVLK

>PA14_68730 | Max Lysine: 3

SLIGRLKYVYRKGLALRYGK

>PA14_68800 | Max Lysine: 3

VRLEQEADRLKKNVRMHLPK

>PA14_68860 | Max Lysine: 3

DPYASWFFKLKPSNPAELDK

>PA14_69020 | Max Lysine: 3

DLQLLTAKFDQLLQKLEQLK

>PA14_69040 | Max Lysine: 3

TRRKNGHKPTLIGLAHECQK

>PA14_69070 | Max Lysine: 3

GLAPFAERPAGKLSGGMKQK

>PA14_69090 | Max Lysine: 3

AAKLPGRVAEIKVDEGDFVK

>PA14_69100 | Max Lysine: 3

LQYYKADIALQVADKDAATK

>PA14_69130 | Max Lysine: 3

GVLDWAPTYLKEAKHFNVDK

>PA14_69250 | Max Lysine: 3

RRHGRRLLARKPRWQKLGDK

>PA14_69300 | Max Lysine: 3

ETQVAQQLELAKGKLAEAAK

>PA14_69320 | Max Lysine: 3

IRLRGAMARKRGEEPVKLRK

>PA14_69330 | Max Lysine: 3

QKAVRRWRGPGFSTPTKNGK

>PA14_69470 | Max Lysine: 3

AVGYLVKPVRSEDLAEALKK

>PA14_69500 | Max Lysine: 3

GSSIMPQKKNPDVPELVRGK

>PA14_69510 | Max Lysine: 3

LFGFLILLSAWWSNHIKYKK

>PA14_69550 | Max Lysine: 3

KVVLEAGAELTLKGGGSFLK

>PA14_69560 | Max Lysine: 3

GQPTGQRVHKPVVITKVFDK

>PA14_69570 | Max Lysine: 3

FAESTAQTRKPKTDLQQIIK

>PA14_69600 | Max Lysine: 3

KVHHTADGKAVAAARRLHDK

>PA14_69610 | Max Lysine: 3

GAGLWQLSKAIDSPYKSLLK

>PA14_69710 | Max Lysine: 3

DLEWLDLKEGLVRVRGKGNK

>PA14_69810 | Max Lysine: 3

MKLVTAIIKPFKLDDVRESL

>PA14_69910 | Max Lysine: 3

KAAREMKERVGTLLRGGEGK

>PA14_69925 | Max Lysine: 3

CLKHRERSLRTLEKEEQAGK

>PA14_69940 | Max Lysine: 3

DILKDKIRSEGIVLSEHVLK

>PA14_69980 | Max Lysine: 3

GARLQTIRKLKGLSQRELAK

>PA14_70060 | Max Lysine: 3

PININLNVKIEHEIYIKVDK

>PA14_70080 | Max Lysine: 3

KARISEMASYRKLLGDILLK

>PA14_70100 | Max Lysine: 3

KLLRSPLLALGMAWYRLKEK

>PA14_70110 | Max Lysine: 3

GELESARRKLQRYLRKESVK

>PA14_70170 | Max Lysine: 3

NSAPEHYSPAPEKILKGDPK

>PA14_70190 | Max Lysine: 3

SEKRFVRLRVSAKGMRIIDK

>PA14_70200 | Max Lysine: 3

EKLNTAPIGSGPFVFKRFQK

>PA14_70240 | Max Lysine: 3

ASAAVADYRPEVVAAHKLKK

>PA14_70280 | Max Lysine: 3

VGKTLVIKYGGNAMESEELK

>PA14_70300 | Max Lysine: 3

HYEARKLNISSFGGKVSDVK

>PA14_70360 | Max Lysine: 3

LDDLKQEEKRLQGEIGRFQK

>PA14_70400 | Max Lysine: 3

GIKANEGKAYRYPFCLRLIK

>PA14_70450 | Max Lysine: 3

LVMLATKRARQLATGGKEPK

>PA14_70490 | Max Lysine: 3

SKDGKLREALLAYGPNLEWK

>PA14_70560 | Max Lysine: 3

SVGVKKLEDELGVLIFERSK

>PA14_70580 | Max Lysine: 3

AALYERLGITPEKAGDAVKK

>PA14_70620 | Max Lysine: 3

ADDGRSYSKPMLSTGFSKNK

>PA14_70640 | Max Lysine: 3

MKKWQCVVCGLIYDEAKGWP

>PA14_70780 | Max Lysine: 3

VFRDRMVEHLEREVKLETKK

>PA14_70810 | Max Lysine: 3

PKSIYENVVYGLRIQGINKK

>PA14_70850 | Max Lysine: 3

NGQVLVFQHTYKVTYPDNKK

>PA14_70950 | Max Lysine: 3

TEHPLIEKISFTGGTSTGKK

>PA14_71020 | Max Lysine: 3

IVLLAILLDRVCKQRAVKGK

>PA14_71060 | Max Lysine: 3

LDKVIRTMRDTGADMHDKYK

>PA14_71150 | Max Lysine: 3

KAELEIFDTGHLWFAKQMLK

>PA14_71180 | Max Lysine: 3

KPLLCIIGEPTELKPVLGHK

>PA14_71200 | Max Lysine: 3

MKNVKQLLEEAGSDLSHIVK

>PA14_71210 | Max Lysine: 3

PDGFPGKDQVAAYFEAYAKK

>PA14_71240 | Max Lysine: 3

DLVIPVRSTADIRKAKEQGK

>PA14_71250 | Max Lysine: 3

AYAEILYKAGYKSAWHWCEK

>PA14_71260 | Max Lysine: 3

ATDGGMTTERYVKYYEEKAK

>PA14_71280 | Max Lysine: 3

GKCEAMCPAFAAGQPLNPKK

>PA14_71420 | Max Lysine: 3

AKLGLHIPKACGMGICGTCK

>PA14_71460 | Max Lysine: 3

KYAEGYPGKRYYGGCEHVDK

>PA14_71500 | Max Lysine: 3

LMGIFGKVGGKLMPPGFYYK

>PA14_71560 | Max Lysine: 3

NPTRLAHAKKQGFEIADLSK

>PA14_71600 | Max Lysine: 3

IGLPAVLKTRTLGYDGKGQK

>PA14_71650 | Max Lysine: 3

MGAFVLFSGMLKRTAVKLSK

>PA14_71720 | Max Lysine: 3

FDACVRFLKEDPWERLRKLK

>PA14_71740 | Max Lysine: 3

ATKAFGSAEVFLEKCIVNPK

>PA14_71750 | Max Lysine: 3

LFEYVGKKLYLTDAAEALLK

>PA14_71820 | Max Lysine: 3

ALGKRFYVVVNIAPHNAKLK

>PA14_71840 | Max Lysine: 3

ERKQWFRTLKSTYADSSWAK

>PA14_71870 | Max Lysine: 3

QTVIEQSGLISYHKEEKGEK

>PA14_71910 | Max Lysine: 3

QYREKTRVITYGLDKDGYPK

>PA14_71930 | Max Lysine: 3

RMFKAKAPVFLMVSTIEPRK

>PA14_71990 | Max Lysine: 3

GIEFVTRKVTDAVARIKLGK

>PA14_72030 | Max Lysine: 3

EIKHLLDHFMPKSQVIIARK

>PA14_72050 | Max Lysine: 3

AIKQALDAVVAKSQVIIARK

>PA14_72080 | Max Lysine: 3

FTAKSLYVTSKLTSTPFENK

>PA14_72170 | Max Lysine: 3

GLFDPAVRKILRLVKGDPLK

>PA14_72180 | Max Lysine: 3

IRMRGAAARKRGEEPVKLRK

>PA14_72210 | Max Lysine: 3

MALWETGLAPGKLSADKLEK

>PA14_72230 | Max Lysine: 3

LGKDIRKLDDDVDALYTAIK

>PA14_72320 | Max Lysine: 3

LIVGALKKVPVYESFVEGAK

>PA14_72340 | Max Lysine: 3

LVVGIAGVGDAKKLGRIGLK

>PA14_72360 | Max Lysine: 3

VDDQGDKYLVGGLCEKSDCK

>PA14_72390 | Max Lysine: 3

PFVQVGRKKGGAGLGLALCK

>PA14_72420 | Max Lysine: 3

DHFKRINDDFGHLAGDKVLK

>PA14_72460 | Max Lysine: 3

FPKLAGQGERYLLKQMHDIK

>PA14_72470 | Max Lysine: 3

QLPMAPKKGDHAAWEPRLAK

>PA14_72500 | Max Lysine: 3

KTIQEYRVNGFLYAIKVVPK

>PA14_72550 | Max Lysine: 3

KAFEERLGGLDGKLRERLGK

>PA14_72600 | Max Lysine: 3

VKKLSPDFDLHLSHVVQADK

>PA14_72720 | Max Lysine: 3

LEKRRLVLENRRLKEQASLK

>PA14_72760 | Max Lysine: 3

GKPQRFKAWEHDMSLRDAIK

>PA14_72840 | Max Lysine: 3

GMGNPDGYKRALLKDYPQGK

>PA14_72850 | Max Lysine: 3

KRLVKGVANRHGLQACFMAK

>PA14_72900 | Max Lysine: 3

KYNDYYEFEQLNGDTILIKK

>PA14_72990 | Max Lysine: 3

SQAKNLDVKVCQQNRSIPAK

>PA14_73020 | Max Lysine: 3

EEQRQWQLRLLEREKKLLDK

>PA14_73040 | Max Lysine: 3

DAGHGGKDPGAVGSKGEREK

>PA14_73090 | Max Lysine: 3

DTYRNMDKVYPTRVIHKGTK

>PA14_73140 | Max Lysine: 3

TGKEDLATAQLAKLRHLCRK

>PA14_73170 | Max Lysine: 3

LHHKLQSIGDLTLALKDAVK

>PA14_73230 | Max Lysine: 3

LDEAAAQEALKAAEKALQGK

>PA14_73240 | Max Lysine: 3

LLETGIKVIDLVCPFAKGGK

>PA14_73280 | Max Lysine: 3

KAEQEKSVEVEVTSAFTLSK

>PA14_73290 | Max Lysine: 3

FCMKFVWPPVIAALQERQKK

>PA14_73310 | Max Lysine: 3

KVKGIGGFLGELTLHPFSSK

>PA14_73330 | Max Lysine: 3

KKRGLGRGLDALLGGSSPAK

>PA14_73350 | Max Lysine: 3

MAKVFAIANQKGGVGKTTTC

>PA14_73390 | Max Lysine: 3

MHARQPGKRFTHKPGRDSAK
